# Transcriptomic Analyses Reveal That *Coffea arabica* and *Coffea canephora* Have More Complex Responses under Combined Heat and Drought than under Individual Stressors

**DOI:** 10.3390/ijms25147995

**Published:** 2024-07-22

**Authors:** Isabel Marques, Isabel Fernandes, Octávio S. Paulo, Dora Batista, Fernando C. Lidon, Ana P. Rodrigues, Fábio L. Partelli, Fábio M. DaMatta, Ana I. Ribeiro-Barros, José C. Ramalho

**Affiliations:** 1 Plant-Environment Interactions and Biodiversity Lab, Forest Research Centre (CEF), Associate Laboratory TERRA, School of Agriculture (ISA), University of Lisbon, 1349-017 Lisboa, Portugal; anadr@isa.ulisboa.pt (A.P.R.); cochichor@mail.telepac.pt (J.C.R.); 2 cE3c—Center for Ecology, Evolution and Environmental Changes and CHANGE—Global Change and Sustainability Institute, Faculdade de Ciências, Universidade de Lisboa, 1749-016 Lisboa, Portugal; isabelcmoniz@gmail.com (I.F.); octavio.paulo@fc.ul.pt (O.S.P.); dorabatista@isa.ulisboa.pt (D.B.); 3 Linking Landscape, Environment, Agriculture and Food (LEAF), School of Agriculture (ISA), University of Lisbon, 1349-017 Lisboa, Portugal; 4 Unidade de Geobiociências, Geoengenharias e Geotecnologias (GeoBioTec), Faculdade de Ciências e Tecnologia (FCT), Universidade NOVA de Lisboa (UNL), 2829-516 Caparica, Portugal; fjl@fct.unl.pt; 5 Centro Universitário do Norte do Espírito Santo (CEUNES), Departmento Ciências Agrárias e Biológicas (DCAB), Universidade Federal Espírito Santo (UFES), São Mateus 29932-540, ES, Brazil; partelli@yahoo.com.br; 6 Departamento de Biologia Vegetal, Universidade Federal Viçosa (UFV), Viçosa 36570-900, MG, Brazil; fdamatta@ufv.br

**Keywords:** climate changes, coffee, drought, functional analysis, heat, stress responses

## Abstract

Increasing exposure to unfavorable temperatures and water deficit imposes major constraints on most crops worldwide. Despite several studies regarding coffee responses to abiotic stresses, transcriptome modulation due to simultaneous stresses remains poorly understood. This study unravels transcriptomic responses under the combined action of drought and temperature in leaves from the two most traded species: *Coffea canephora* cv. Conilon Clone 153 (CL153) and *C. arabica* cv. Icatu. Substantial transcriptomic changes were found, especially in response to the combination of stresses that cannot be explained by an additive effect. A large number of genes were involved in stress responses, with photosynthesis and other physiologically related genes usually being negatively affected. In both genotypes, genes encoding for protective proteins, such as dehydrins and heat shock proteins, were positively regulated. Transcription factors (TFs), including MADS-box genes, were down-regulated, although responses were genotype-dependent. In contrast to Icatu, only a few drought- and heat-responsive DEGs were recorded in CL153, which also reacted more significantly in terms of the number of DEGs and enriched GO terms, suggesting a high ability to cope with stresses. This research provides novel insights into the molecular mechanisms underlying leaf *Coffea* responses to drought and heat, revealing their influence on gene expression.

## 1. Introduction

Water deficit and high temperatures are major abiotic factors limiting crop production worldwide. Global climate change is expected to further increase the frequency and severity of drought spells, as well as high temperatures and heat waves [1,2,3]. Therefore, understanding the molecular mechanisms supporting plants’ acclimation capabilities (and their limits) to such aggravating environmental constraints has become a key research topic to cope with the impacts of climate change [4,5].

High temperatures can lead to greater stomatal conductance (g_s_) due to a greater need to increase the dissipation of the latent heat occurring in the leaf. Still, despite increases in transpiration in most species, g_s_ is also frequently found to decline under a high vapor pressure deficit (VPD), leading to subsequent impacts that include reduced photosynthesis and growth, as well as C-starvation and hydraulic failure [6,7,8]. In fact, heat might impose constraints on physiological, biochemical, and molecular processes, causing protein denaturation and aggregation, altering hormones and primary and secondary metabolite balance, and inhibiting transcription and translation [2,9,10]. Regarding the photosynthetic pathway, heat alters gas diffusion through the leaf mesophyll, and even below heat-damage levels, it stimulates respiration and photorespiration more than photosynthesis. Additionally, for the latter pathway, harsher heat conditions have been reported to promote extensive impacts on pigments, membrane fluidity, thylakoid electron transport, ribulose-1,5-bisphosphate carboxylase/oxygenase (RuBisCO) activity, and the overproduction of ROS [2,11,12], altogether depressing plant growth and crop yields [13,14].

Drought is also a major threat to agriculture sustainability and can lead to the loss of cell turgor and water content, limiting plant growth and dry mass accumulation. Additionally, it compromises photosynthesis and C-assimilate partitioning and reduces nutrient uptake, contributing to important negative impacts on crop yields [10,15,16]. Under mild drought, C-assimilation is mostly limited by the supply of CO_2_ to RuBisCO due to stomatal closure. However, under severe drought, non-stomatal limitations associated with photochemical and biochemical impacts have growing importance due to impairments in the components of the photosynthetic apparatus, including the activity of photosystems and key enzymes such as RuBisCO [15,17,18,19,20]. The triggering of ROS overproduction can further prompt multiple forms of damage to cell structures/molecules, namely, through lipid peroxidation and the further inactivation of the Calvin cycle enzymes [2,21,22].

Under climate change, the combination of high temperatures and drought is becoming an increasingly recurrent event [10,23,24]. Extreme high temperatures can accelerate soil evaporation and increase plant transpiration due to a greater vapor pressure deficit between the leaf and the surrounding air, thus aggravating the degree of drought stress [6]. On the other hand, considering that drought promotes stomatal closure, at high temperatures (and irradiance), plants will face transpiration restrictions, which will greatly reduce latent heat dissipation in leaves. The leaf’s evaporative cooling process through transpiration is an important heat avoidance mechanism, optimizing leaf temperature to its biochemical functioning [9]. It significantly reduces leaf/canopy temperature by as much as 5 °C [25,26] or even 9 °C in poplar at high temperatures [27] in comparison with the temperature in the surrounding air. Yet, such performance can be greatly reduced to 1 °C with the co-occurrence of drought (and stomatal closure) in poplar [27]. Therefore, this restriction will likely exacerbate the negative impacts imposed by exposure to a single stressor [11,28,29], since photosynthetic and metabolic thermal tolerance might be overcome, with significant impacts on crop yield and quality as well [30]. This could be harsher in plants (such as some woody tropical Amazonian ones) that display a low ability to maintain stable leaf thermal safety margins, thus overcoming photosynthetic thermal tolerance and increasing their vulnerability as air temperature rises [31].

Coffee (*Coffea* L.) is among the most valued agricultural commodities in international trade, estimated to support the livelihoods of 25 million smallholder farmers in approx. 80 tropical coffee-producing countries and about 100–125 million people considering the entire worldwide chain of value [32]. The coffee trade is dominated by *C. arabica* L. and *C. canephora* Pierre ex A. Froehner species, which are responsible for nearly 99% of global coffee production. Since elevated temperature and water scarcity are known as the major abiotic constraints for the *Coffea* crop, negative impacts on bean yield and quality are expected to occur in the near future in many coffee-producing regions due to the exacerbation of climatic changes and global warming. In addition, coffee is extensively cropped under full sunlight exposure in many countries, which can further exacerbate the impacts of high temperatures and drought and reduce the suitability of certain regions for this crop during the current century [33]. This demands the implementation of new management strategies, such as agro-forestry systems that provide shade to *Coffea* plants [34] and promote favorable microclimate modifications close to plants, such as lower air temperature fluctuations, increased air relative humidity, and lower wind speed while minimizing water loss by reducing soil evaporation and crop transpiration [35]. This was perceived as particularly important for *C. arabica*, which is globally considered inherently more sensitive to heat, drought, pests, and diseases than *C. canephora* [36]. Yet, recent studies claim that *C. canephora* bean productivity and quality are also highly sensitive to high temperatures and low rainfall availability [37,38], making these issues more important than initially assumed regarding the two main producing species.

Some drought-tolerant *Coffea* genotypes can reduce g_s_ to avoid excessive transpiration, although triggering antioxidant molecules [19]. Also, the photosynthetic machinery can cope with temperatures up to 37 °C, even with co-occurring drought [11,32,39]. These findings point to the intrinsic resilience of some cultivars to severe drought and/or heat conditions, higher than traditionally assumed, even considering the exposure to moderate/low irradiance in these experiments, which is usually not found in *Coffea* plantations grown under mono-crop management. Since the predicted scenarios of climate changes point to a future greater exposure to single and combined heat and drought stresses, understanding the underlying molecular mechanisms by which *Coffea* plants can deal with these harsher environmental conditions is of utmost importance for the future sustainability of this crop. In this context, this study deals with the effects of high temperature and/or drought on the transcriptomic machinery of two genotypes from the most important coffee-traded species: *Coffea canephora* Pierre ex A. Froehner cv. Conilon Clone 153 (CL153) and *C. arabica* L. cv. Icatu Vermelho (Icatu). Plants grown at 25 °C were progressively subjected to severe water deficit (SWD) conditions and compared with their well-watered (WW) counterparts, and thereafter, they were gradually exposed to temperature increases to 37 °C and further to 42 °C. We aimed to explore the transcriptomic machinery responses, contributing to explaining the relevant physiological and biochemical resilience to up to 37 °C and SWD and the sensitivity to 42 °C (regardless of water availability level) reported in Icatu and CL153. Understanding the molecular mechanisms that ultimately determine the response limits of the main producing *Coffea* species to climatic changes is crucial to mitigating their harmful effects and establishing better scenarios for the future sustainability of this crop and its value chain.

## 2. Results

### 2.1. Overall Transcriptome Profiling and Mapping Statistics

RNA sequencing generated an average of 22.4 and 21.1 million high-quality reads from Icatu and CL153 plants, respectively. Overall, an average of 91% and 89% of reads were mapped to the corresponding reference genome of Icatu and CL153, with 73% and 85% being mapped uniquely, respectively (Table S1).

### 2.2. Number of Expressed Genes in Response to Severe Drought and High Temperatures

**Figure 1:**
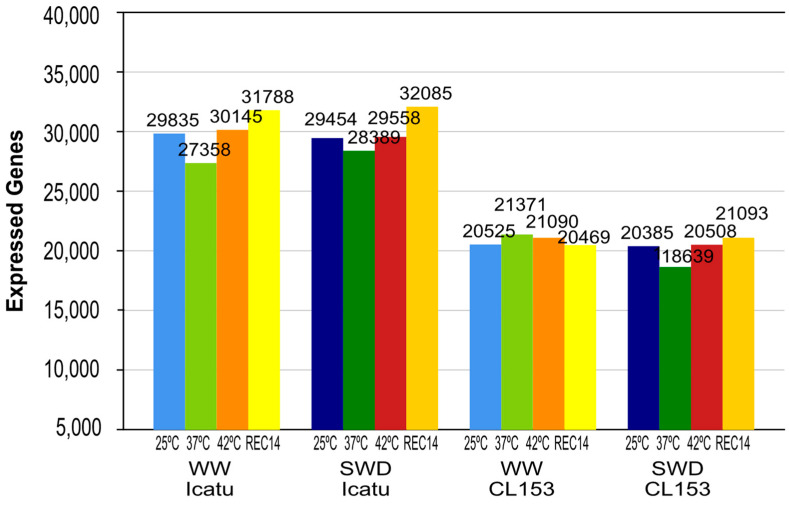
Total number of expressed genes in *Coffea arabica* cv. Icatu and *C. canephora* cv. Conilon Clone 153 (CL153) plants grown under well-watered (WW; light colors) and control temperature (25 °C; blue) conditions before gradual exposure to severe water deficit (SWD; dark colors). Afterward, WW and SWD plants were additionally exposed to increased temperatures of 37 °C (green) and 42 °C (orange), followed by a 2-week recovery period (REC14, yellow) with full rewatering and a temperature of 25 °C.

Regardless of water availability or temperature increases, CL153 plants expressed a much lower number of genes than Icatu in all treatments (Figure 1). Under WW or SWD conditions, Icatu plants expressed the lowest number of genes at 37 °C and the highest after REC14 (Figure 1; Table S2).

This pattern changed in CL153. Under WW conditions, CL153 plants expressed the lowest number of genes after REC14 and the highest at 37 °C, while under SWD, the lowest number of expressed genes was recorded at 37 °C and the highest after REC14 (Figure 1; Table S2).

### 2.3. Quantitative Changes in the Number of DEGs

**Figure 2:**
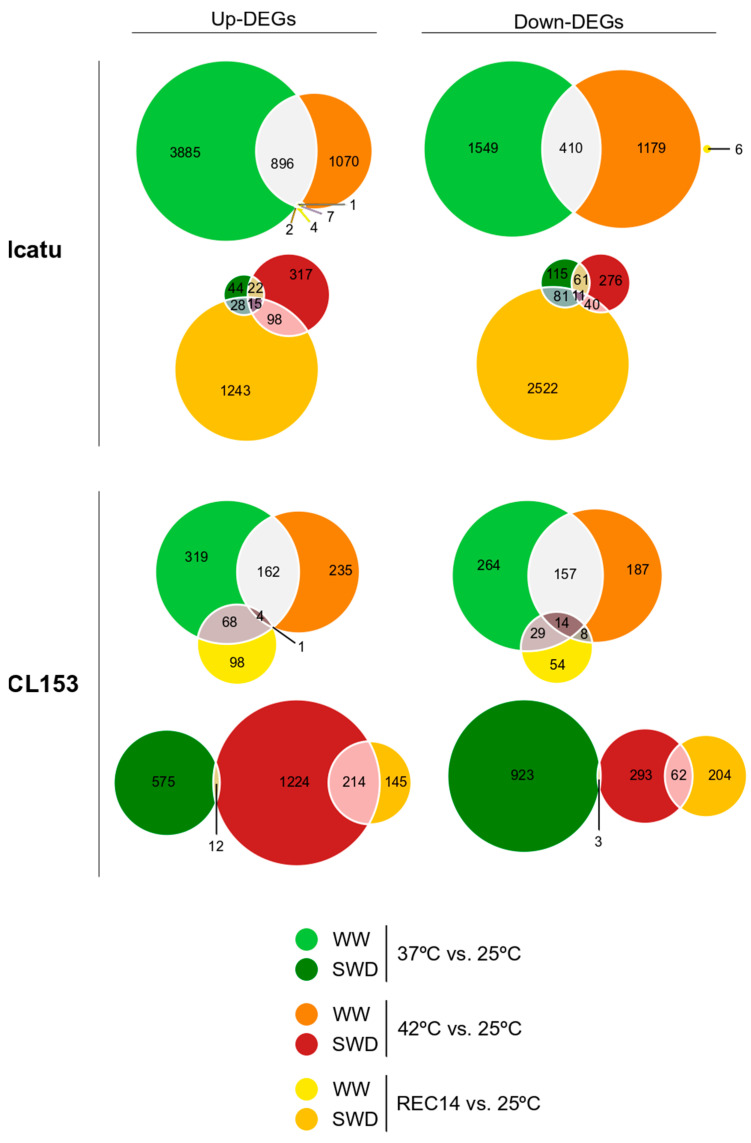
Differentially expressed genes (DEGs) relative to initial control conditions (25 °C, WW, light colors) in *Coffea arabica* cv. Icatu and *C. canephora* cv. Conilon Clone 153 (CL153) plants after gradual exposure to severe water deficit (SWD, dark colors) and to increased temperatures of 37 °C (green) and 42 °C (red), followed by a 2-week recovery period recovery (REC14, yellow) with full rewatering and a temperature of 25 °C. Intersections between 37 °C and 42 °C (gray), 42 °C and REC14 (pink), 37 °C and REC14 (ashy), and 42 °C and REC14 (violet) are shown.

Under WW conditions, DEGs decreased from 37 °C to 42 °C and decreased even further after REC14 (Figure 2). Between genotypes, this decline was particularly found in Icatu, although this genotype always showed a much higher number of DEGs (both up- and down-regulated) than CL153 (Figure 2). Between temperatures, exposure to 37 °C triggered the greatest up- and down-regulation of DEGs in both genotypes (Icatu: 3885 and 1569; CL153: 319 and 264, respectively). At 42 °C, this pattern slightly changed in Icatu (Icatu: 1070 and 1179), but not in CL153 (235 and 187), for up- and down-regulated DEGs, respectively. It is noteworthy that the much lower number of DEGs reported after REC14 in both genotypes is almost zero in Icatu (4 up and 6 down DEGs) when compared to CL153 (98 up and 54 down).

Exposure to the combined stress promoted different quantitative responses in the two genotypes, contrasting with the single exposure (Figure 2). For instance, generally, the two genotypes showed a considerably higher number of DEGs at 42 °C than at 37 °C (mostly up-regulated). However, CL153 plants displayed a higher number of DEGs at 42 °C, both up- and down-regulated (1224 and 293), than their Icatu counterparts (317 and 276). At the end of the recovery period (REC14), the number of DEGs strongly increased in Icatu (1243 and 2522 up- and down-regulated DEGs) but declined in CL153 (145 and 204a up- and down-regulated DEGs).

Finally, it is also worth noting the high number of DEGs commonly expressed (both up- and down) in WW plants at 37 °C and 42 °C, which declined under SWD in both genotypes, but with greater numbers in Icatu (Figure 2).

### 2.4. Responsiveness DEGs

An average of 72% (Icatu) and 81% (CL153) of DEGs were annotated according to their reference genome, while the remaining were uncharacterized proteins or proteins with unknown functions.

Considering all annotated DEGs under WW conditions and 37 °C, Icatu mostly up-regulated those involved in glycosyltransferase activities, namely, the UDP-glycosyltransferase 708C2-like gene, while down-regulated ones were associated with heat shock protein (HSP) genes (e.g., 22.0 kDa class IV HSP-like) and the caffeic acid 3-O-methyltransferase-like gene (Table S3). With a further temperature rise to 42 °C, Icatu mostly up-regulated enzyme inhibitor activities (such as the 21 kDa protein-like gene), while its most down-regulated genes included several HSPs (such as 22.0 kDa class IV, 17.3 kDa class II, and 18.5 KDa class I, as well as 70 kDa protein-like) (Table S4).

Under heat and drought combined, the DEGs changed. For instance, Icatu-SWD plants at 37 °C mostly up-regulated genes involved in glycosyltransferase (like WW plants at this temperature) and defense activities (such as galactinol synthase) or involved in plant morphogenesis (such as the exocyst gene *EXO70H1*). Among down-regulated DEGs, the caffeic acid 3-O-methyltransferase-like gene stood out (as in the case of the single 37 °C exposure) (Table S3). At 42 °C, Icatu-SWD plants up-regulated lipid binding genes but mostly down-regulated several HSPs (which was also found under WW conditions at this temperature) (Table S4).

During REC14, Icatu-WW plants mostly up-regulated DNA binding DEGs (such as NAC domain-containing protein 72), while down-regulated genes were involved in hydrolase activities (such as carboxylesterase 15) (Table S5). On the other hand, in Icatu-SWD plants, genes involved in glycosyltransferase activities were both up- and down-regulated (Table S5).

The transcriptomic responses of CL153 plants exposed only to 37 °C (thus, CL153-WW plants) involved the up-regulation of heat-responsive DEGs, namely, several HSP genes (such as 18.5 kDa class I, 22.7 kDa class IV, 18.2 kDa class II, and 70 kDa proteins), while ion binding genes were down-regulated (Table S6). Upon exposure to the harsher 42 °C condition, the up-regulation of several HSPs was also observed (in contrast with Icatu-WW plants at both 37 and 42 °C), together with the down-regulation of DEGs involved in ATP-binding and kinase activity (Table S7).

Clear differences from CL153-WW plants were observed in the ones exposed to the combination of drought (SWD) and heat (37 °C). At this temperature, these CL153-SWD plants mostly up-regulated genes involved in oxidoreductase activities, such as reticuline oxidase genes, while down-regulating ATP binding and ion channel activity genes (Table S6). At 42 °C, SWD plants showed an up-regulation of HSPs (as in CL153-WW plants at the same temperature and contrasting with Icatu-SWD plants) and lipid metabolism genes (similar to the up-regulation of lipid binding genes in Icatu-SWD plants at 42 °C). Down-regulated DEGs included those linked to reticuline oxidase-like proteins, peroxidases, and cytochrome P450 genes (Table S7).

After the recovery period (REC14), CL153-WW plants up-regulated several DEGs involved in glycosyltransferase activities but also down-regulated binding and oxidase genes like the NAD(P)-binding Rossmann-fold superfamily and cytochrome P450 (Table S8). In contrast, plants that were previously subjected to the stress combination (CL153-SWD) up-regulated genes involved in defense (Laccase-4) and the likely recovery of chloroplast structures (Chlorophyll a-b binding protein 1) but also down-regulated DNA binding and oxidoreductase genes such as the ABC transporter or glutaredoxin (Table S8).

### 2.5. Regulation of Water Deprivation and Desiccation DEGs

A search for genes involved in water deprivation and desiccation responses found 128 responsive DEGs in Icatu (Table S9). Overall, in Icatu-WW plants, DEGs were mostly up-regulated at both 37 °C and 42 °C (45 and 32, respectively) than down-regulated (7 and 20, respectively). Under the combination of drought and heat, a very low number of water-responsive DEGs were found in Icatu, with only 4 DEGs found at 37 °C (3 up- and 1 down-regulated) and 14 at 42 °C (13 up- and 1 down-regulated), while a strong down-regulation of DEGs was recorded after REC14 (57 DEGs; Table S9).

Table 1 shows the five most up- and down-regulated drought-responsive DEGs in Icatu plants, which are involved in general defense mechanisms but also in specific responses, such as the transport of water and small molecules through aquaporins. The non-specific lipid-transfer protein 1-like gene was the most up-regulated DEG at 37 °C, while annexin D4-like, followed by the aquaporin *PIP1-2*, was highly up-regulated at 42 °C. It is worth mentioning that several aspartic proteases in guard-cell 1 genes were also up-regulated at both temperatures (Table S9), while genes involved in general functions such as transport and binding were down-regulated at both temperatures (Table 1). After REC14, only two DEGs were identified, both up-regulated and related to NAC domain-containing protein 72-like.

Under the combination of drought and heat, protein phosphatase 2C 51-like was the most regulated DEG at 37 °C, and at 42 °C, it was the non-specific lipid-transfer protein. Chlorophyll a-b binding protein 36 and the endoplasmin homolog were the only down-regulated DEGs found at 37 °C and 42 °C, respectively. During REC14, Icatu-SWD plants mostly up-regulated DEGs involved in galactinol synthase while strongly down-regulating genes involved in general biological functions (Table 1).

In contrast with Icatu, only 19 DEGs associated with water responses were recorded in CL153, considering all conditions (Table S10). The combined action of heat and drought led to an even lower number of DEGs in CL153-SWD plants (four at 37 °C and six at 42 °C) (Table S10).

Table 2 shows the five most up- and down-regulated drought-responsive DEGs in CL153 plants, which are mostly involved in general biological functions but also in defense mechanisms. Under WW conditions, at 37 °C, all responsive DEGs (only five) were up-regulated (e.g., Laccase-2, Cellulose synthase A catalytic subunit 8, and Protein aspartic protease in guard-cell 1 genes), whereas at 42 °C, only two were detected as responsive, both down-regulated (Histidine kinase 4 and Mitogen-activated protein kinase 3). After REC14, CL153 plants showed only one DEG (up-regulated, the cellulose synthase A gene).

Under the combined action of heat and drought, the most up-regulated DEG at 37 °C was a putative aspartic protease in guard-cell 1, while at 42 °C, it was the Cellulose synthase A catalytic subunit 8 gene. At these temperatures, two general DEGs were down-regulated at 37 °C, while it is worth mentioning the down-regulation of aspartic proteases at 43 °C (Table 2). Several DEGs were triggered after stress ended (REC14), including the simultaneous up- and down-regulation of aspartic protease in guard-cell 1 genes (Table 2).

### 2.6. Regulation of Heat-Responsive DEGs

A search for genes involved in heat responses found a total of 165 DEGs in Icatu (Table S11) but only 29 DEGs in CL153 (Table S12). Under WW conditions, Icatu plants at 37 °C mostly up-regulated DEGs (52 vs. 29, up vs. down DEGs), while the opposite was found at 42 °C (2 vs. 18, up vs. down DEGs) and after REC14 (43 vs. 45, up vs. down DEGs; Table S11). Under the stress combination, DEGs were mostly down-regulated at 37 °C in Icatu-SWD plants (10 vs. 28, up vs. down DEGs), while at 42 °C, only 1 was found (Table S11).

Table 3 shows the five most up- and down-regulated heat-responsive DEGs in Icatu plants. Several genes involved in general processes, such as the auxin-binding *ABP20* and an acidic endochitinase, were up-regulated at 37 °C and 42 °C, respectively (Table 3). In contrast, several HSPs were already down-regulated at 37 °C, but also at 42 °C, and even after REC14. During REC14, Icatu plants up-regulated many general genes, but several HSPs were found to be strongly down-regulated (Table 3).

The stress combination triggered the up- and down-regulation of DEGs involved in general functions, but it is worth mentioning the down-regulation of several HSPs at 37 °C. At 42 °C, only one up-regulated DEG was found (xyloglucan endotransglucosylase/hydrolase protein 22-like; Table 3). During REC14, plants up-regulated general metabolic genes, namely, several galactinol synthase genes, as well as one HSP (Table 3).

In CL153 plants, a small proportion of DEGs were detected. These were mostly up-regulated under WW conditions (37 °C: 12 vs. 1; 42 °C: 13 vs. 1; up vs. down DEGs), while under the combination of heat and drought, most were down-regulated at 37 °C (2 vs. 14, up vs. down DEGs) but again up-regulated at 42 °C (8 vs. 2, up vs. down DEGs) (Table S12).

Table 4 shows the most up- and down-regulated heat-responsive DEGs in CL153 plants. Under WW conditions, most up-regulated DEGs were involved in general functions but also in heat responses, such as HSPs, which were found at 37 °C and 42 °C.

Under the combination of heat and drought, most DEGs were down-regulated at 37 °C (including RuBisCO genes) but up-regulated at 42 °C, namely, HSPs (Table 4).

In contrast with Icatu, a very low number of heat-responsive DEGs were found after REC14, either under WW (one up-regulated DEG) or under SWD conditions (four vs. one, up vs. down DEGs), and most involved binding regulatory genes (Table 4).

### 2.7. Regulation of Transcription Factors

A total of 145 DEGs were annotated as transcription factors (TFs) in Icatu (Table S13), compared to 65 in CL153 (Table S14).

In Icatu-WW plants, TFs were mostly up-regulated and increased as the temperature increased (37 °C: 42 vs. 5; 42 °C: 45 vs. 5; up vs. down TFs), dropping during REC14 (2 up TFs). Under the combination of drought and temperature, a very low number of TFs (all up-regulated) were recorded in Icatu-SDW plants (37 °C: 3; 42 °C: 8 TFs), except after REC14, where a high number of TFs were regulated (42 vs. 57, up vs. down TFs). TFs included several ethylene-responsive, NAC domain, and MYB TFs genes that were up-regulated, especially at 37 °C and under WW conditions. In contrast, down-regulated TFs included MADS-box, auxin response, and general regulatory genes, mainly identified under SWD and REC14 conditions (Table S13).

CL153-WW plants showed a much lower number of TF-DEGs at high temperatures than Icatu-WW plants (Table S14). TFs were mostly down-regulated under WW conditions (37 °C: 8 vs. 11; 42 °C: 6 vs. 7; up vs. down TFs), even after REC14 (1 vs. 5, up vs. down TFs). Several of the up-regulated TFs in CL153-WW plants (at 37 and/or 42 °C) were involved in stress responses, such as heat stress or dehydration-responsive element-binding TFs, while down-regulated TFs included regulatory factors and MADS-box genes (Table S14). It is also noteworthy to find that, after REC14, only AP2/ERF domain-containing TFs were up-regulated in CL153-WW plants.

Under the stress combination, CL153-SWD plants did not show great differences in numbers at either 37 °C (13 up- vs. 10 down-regulated) or 42 °C (5 up- vs. 5 down-regulated), and DEGs were mostly down-regulated after REC14 (13 vs. 19, up vs. down TFs; Table S14). Up-regulated TFs were mostly involved in stress responses, while down-regulated ones involved regulatory factors and MADS-box genes (Table S14). For instance, at 37 °C, CL153 plants mostly up-regulated the heat shock factor protein HSF30 under WW conditions and the ethylene-responsive transcription factor 1B gene under SWD. In contrast, at 42 °C, the heat stress transcription factor A-6b was the most up-regulated DEG under WW conditions, while under SWD, it was the ethylene-responsive transcription factor TINY-like gene. During REC14, CL153-SWD plants mostly activated the myb-like HTH transcriptional regulator family protein (Table S14).

### 2.8. Impacts on Specific Biochemical and Photosynthetic Pathways

**Figure 3:**
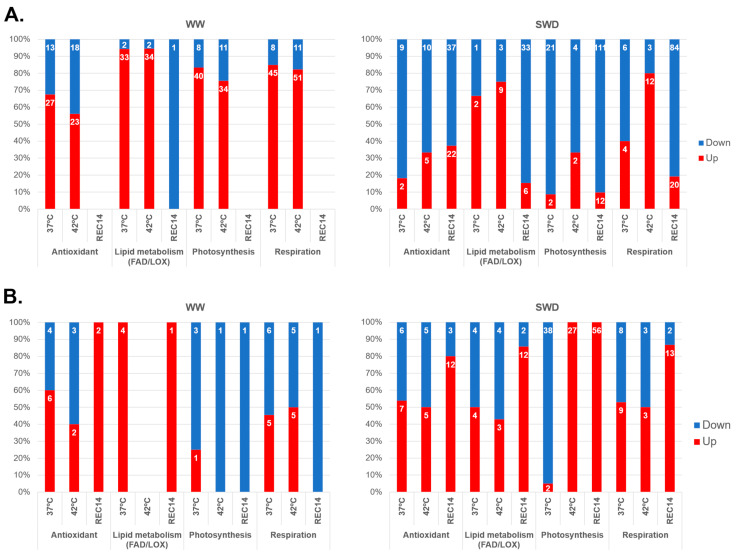
Proportion of significantly up- (red) and down-regulated (blue) DEGs associated with antioxidant activities, lipid metabolism, photosynthesis, and respiration in Icatu (**A**) and CL153 (**B**). Treatments are as explained in Figure 2.

Under WW conditions, heat had a relevant impact on a high number of DEGs associated with antioxidant, lipid metabolism, and cellular energy (photosynthesis and respiration) pathways, affecting a total of 450 DEGs (Figure 3; Table S15) in Icatu, in comparison with the 210 DEGs found in CL153 (Figure 3; Table S16).

In Icatu plants under WW conditions, DEGs associated with these biochemical pathways were mostly up-regulated at 37 °C and 42 °C, but after REC14, no DEGs were found (Figure 3). In contrast, the combination of drought and heat led to the down-regulation of most of these DEGs at both temperatures, except lipid metabolism at 37 °C and 42 °C and respiration at 42 °C (Figure 3).

In CL153 plants under WW conditions, the increase in temperature also led to the up-regulation of most DEGs, except those related to photosynthesis at 42 °C and during REC14 and those related to respiration during REC14 (Figure 3). Under SWD, a high number of down-regulated DEGs were found with a clear differentiation of up-regulated photosynthetic DEGs at 42 °C (Figure 3). A high number of up-regulated DEGs were reported after REC14 in relation to antioxidants, lipid metabolism, photosynthesis, and respiration (Figure 3).

### 2.9. Enriched Gene Ontology (GO) Terms

**Figure 4:**
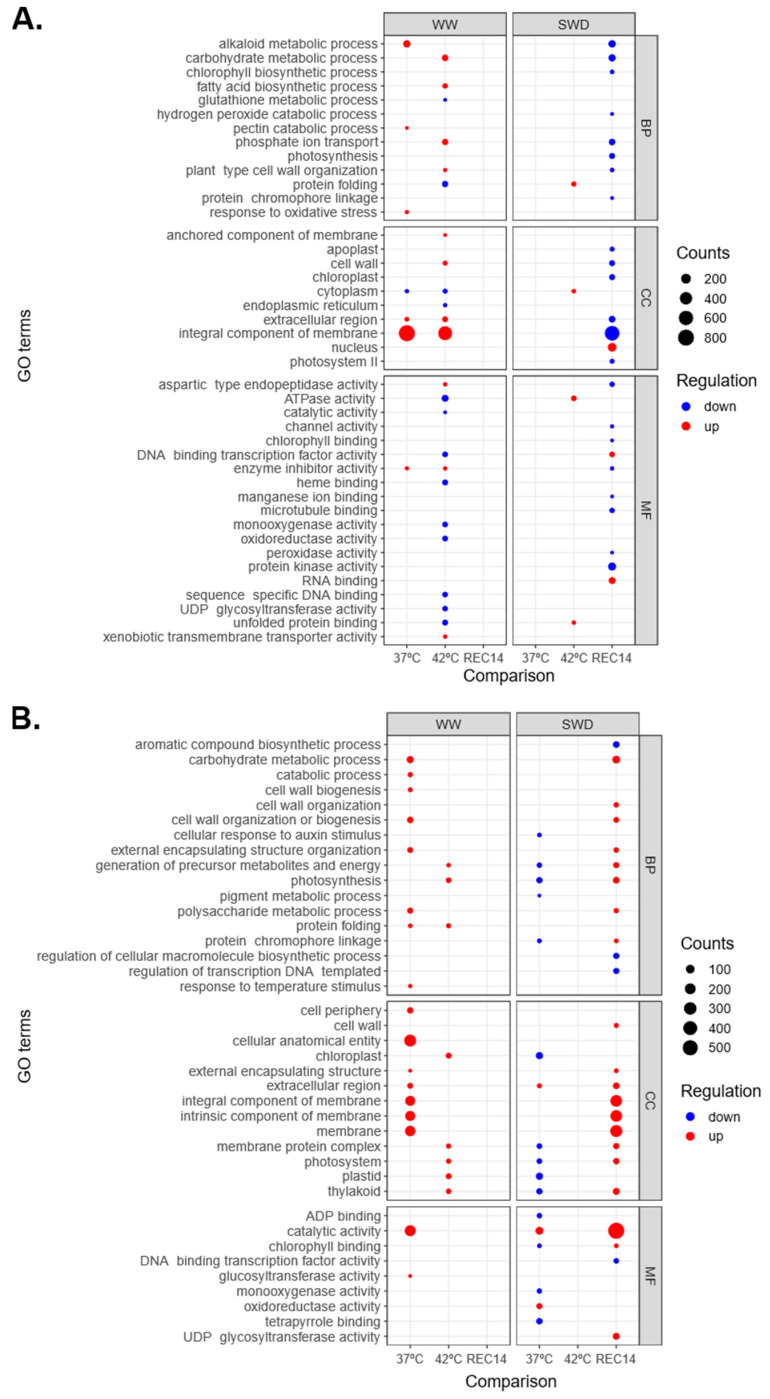
Over-representation analysis of Gene Ontology (GO) terms performed with gProfiler against functional annotations in Icatu (**A**) and CL153 (**B**). GO terms are grouped by main category—Biological Process (BP), Molecular Function (MF), and Cellular Component (CC). Counts (size) indicate the number of DEGs annotated with each GO term, and dots are colored by the adjusted *p*-value (red: up-regulated DEGs; blue: down-regulated DEG). Treatments are as explained in Figure 2.

Under WW conditions, Icatu plants at 37 °C showed a slight enrichment in a few GO terms, with the “Integral component of membrane” being highly enriched (Figure 4). The same significant GO term was reported at 42 °C, although most other enriched GO terms were down-regulated. No significant GO terms were recorded after REC14 or under SWD at 37 °C. At 42 °C and under SWD and heat, there was almost an absence of enriched GOs (only four). After REC14, there was a large number of down-regulated enriched GO terms, especially the “Integral component of membrane” (Figure 4).

Clear differences were found in CL153 plants where, contrary to Icatu, all enriched GO terms were up-regulated under WW conditions, either at 37 °C or 42 °C (Figure 4). In REC14, no significant pathways were found, as in Icatu plants. Under SWD, most enriched GO terms were down-regulated at 37 °C, while none was detected at 42 °C (Figure 4). REC14 plants up-regulated most GO terms, namely, the “Catalytic activity” (Figure 4). Among these, “Catalytic activity” and those associated with membranes (“Integral component of membrane”, “Intrinsic component of membrane”, “membrane”) showed the highest values (Figure 4). This contrasts with Icatu-SWD plants, which showed a large majority of down-regulated GOs terms, including “Integral component of membrane”.

### 2.10. Validation of RNA-Seq Results by qRT-PCR

**Figure 5:**
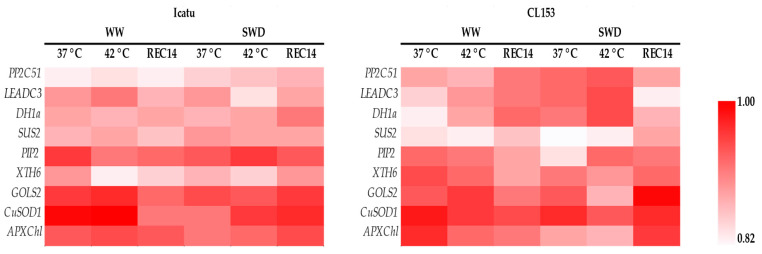
Correlation results between RNA-seq data and qRT–PCR expression levels considering the following selected genes: *PP2C51*, protein phosphatase 2C 51-like; *LEADC3*, late embryogenesis abundant protein Dc3-like; *DH1a*, dehydrin DH1a; *SUS2*, sucrose synthase 2-like; *PIP2-2*, aquaporin PIP2-2-like; *XTH6*, xyloglucan endotransglucosylase/hydrolase protein 6; *GOLS2*, galactinol synthase 2-like; *CuSOD1*, superoxide dismutase [Cu-Zn]; *APXChl*, chloroplast ascorbate peroxidase.

The results showed a high correlation between the expression levels of all genes identified by the two technologies, indicating the validity of the RNA-seq results (Figure 5).

## 3. Discussion

### 3.1. Gene Expression under Stress: Resilience and Divergent Genotype Responses Are Supported by Previous Physiological Findings

*Coffea* genotypes usually have a limited optimal temperature range, with most *C. arabica* genotypes having an ideal annual mean temperature between 18 and 23 °C, while in *C. canephora*, this range is higher (22–30 °C) [36]. Recent studies suggest that high temperatures might have strong impacts on *C. arabica* production [37,40], especially under full sunlight exposure [34]. Likewise, *C. canephora* was recently suggested to be more sensitive to global warming than previously thought, with reports that every 1 °C increase in mean minimum/maximum temperatures above approx. 24 °C can lead to yield declines of ~14% or 350–460 kg ha^−1^ [37]. However, despite the relevant sensitivity of most *Coffea* genotypes to environmental constraints, the study of elite cultivars has been quite overlooked, although some of them (as the ones studied here) can present strong intrinsic resilience to harsh conditions of water restriction and high temperatures, well above commonly believed [11,39,41]. In fact, the genotypes studied here showed reinforced resilience under elevated air [CO_2_] as well (e.g., [19,42]), which is quite relevant when envisaging this crop’s sustainability from the perspective of future climate changes.

Previously published physiological studies of these genotypes found a strong photosynthetic tolerance in WW plants of both genotypes to temperatures well above those considered adequate for the *Coffea* crop (e.g., 37 °C), demonstrating an overall high tolerance to heat, especially when the water level is not limited [11,39,43]. Physiological and biochemical studies revealed a strong heat tolerance in these genotypes, with significant increases in the activities of RuBisCO (up to almost 50% in CL153 and Icatu at 37 °C, relative to 25 °C), although with strong impairments at 42 °C [11,42]. Indeed, at this harsh temperature, potential photosynthetic values (Amax, PS activities) were markedly reduced (minimum 65% reduction in their maximum activities), indicating that a clear thermal threshold was overcome in both genotypes [11], in agreement with the present transcriptomic results reported herein.

The transcriptomic results found in this study support these physiological findings by several factors. For instance, under WW conditions, transcripts were scarcely affected by the temperature increase up to 37 °C, with numbers even declining in Icatu and slightly expanding in CL153. Additionally, under WW conditions, both genotypes showed an increase in the number of transcripts at 42 °C when compared to 25 °C, although quantitative changes were not substantially high and even less in the case of CL153. This genotype expressed fewer genes than Icatu in all treatments (Figure 1). Finally, the combination of SWD and heat did not trigger relevant quantitative changes since, except for Icatu plants at 37 °C and after REC14, the scarcity of water together with temperature increases triggered a lower number of transcripts in plants (Figure 1).

The different transcriptomic responses found between the two genotypes, independently of the imposed stress, also suggest that they respond differently (Figure 1). The minor transcriptomic changes triggered by heat in CL153 plants were also reported in a previous study, where it was suggested that this genotype might be less affected than Icatu, at least under ambient levels of CO_2_ [44].

### 3.2. High-Temperature and Drought Impacts on the Transcriptomic Profile

The combination of drought and heat triggered a significant transcriptional response/reprogramming in the two genotypes (Figure 2), suggesting that the action of combined stresses is not just a simple additive effect but is rather perceived as a new threat by *Coffea*. The combined effects of heat and stress have been shown to trigger strong transcriptomic responses in other plants as well [45,46,47]. In nature, plants are commonly exposed to concomitant stresses that activate distinct, and often antagonistic, defense signaling pathways. For example, a transcriptome study of 10 *Arabidopsis thaliana* ecotypes subjected to single and combined cold, heat, high-light, salt, and flagellin treatments found that 61% of the transcriptome changes in response to the combined stresses could not be extrapolated from individual stresses [24]. Despite this evidence, the molecular mechanisms underlying such changes are scarcely understood in most species (including *Coffea*) due to the limitation of studies (and experimental facilities) dealing with multiple stresses [48].

Our study showed that Icatu sharply reduced the number of DEGs under combined stresses (as compared with heat alone), whereas CL153 showed the opposite pattern (Figure 2). This observation suggests that CL153 may have a “primed” transcriptome that is generally active and is further triggered by combined stresses. For instance, impacts on the transcripts linked to selected physiological and metabolic processes showed that CL153 was less reactive than Icatu (Figure 3). A clear example was the fact that, in Icatu, the GO term “Integral component of membrane” contained the largest number of up-regulated DEGs after exposure to heat alone (that is, in WW conditions and at both temperatures), although none was reported under the combined action of drought and heat, and they were even down-regulated after REC14 (Figure 4). This seems of utmost importance, knowing that under biotic or abiotic stresses, the cellular membranes act as primary sites for perceiving these environmental cues and play crucial roles in acclimation responses [49].

Icatu-SWD plants at high temperatures showed a significant down-regulation of DEGs (Figure 2), including ones related to photosynthetic and biochemical processes (Figure 3). In addition, there was an under-representation of GO terms that persisted in REC14 plants, contrary to CL153, where GO terms were highly enriched (Figure 4). Thus, the reposition of control water and temperature conditions activated the differential expression of genes in the two genotypes in the recovery period, although all were involved in stress responses. For instance, under WW conditions, Icatu plants up-regulated many DNA binding genes, namely, the NAC domain-containing protein 72 (Table S2), particularly in REC14 (Table 1 and Table S9), or genes involved in glycosyltransferase activities under the combination of drought with heat (Table S5). These last ones were up-regulated in CL153 plants under WW conditions, while heat and drought activated defense and metabolic genes such as Laccase-4 or Chlorophyll a-b binding protein 1 (Table S8). NAC proteins make up one of the largest families of plant-specific transcription factors (TFs) and play essential roles in stress responses and plant development, acting together with ABA-dependent or ABA-independent pathways ([50,51]; see also below for information on other TFs). Laccase plant enzymes also have essential functions during growth and development, and their expression has been related to environmental stresses [52]. Light-harvesting chlorophyll (*LHC*) a/b binding genes also play a vital role in photosynthetic processes and are involved in the regulation of plant growth, development, and responses to abiotic stress [53]. It is also worth mentioning the down-regulation of the caffeic acid 3-O-methyltransferase-like gene (*COMT*) under heat stress. Our results show that *COMT* is down-regulated in Icatu plants already at 37 °C and even under WW conditions (Table S2), but mostly under the combination of heat and drought (Table S3). *COMT* catalyzes the multi-step methylation reactions of hydroxylated monomeric lignin precursors and is a critical gene in the regulation of lignin production in cell walls and plant growth and development [54]. As one of the key components of the cell wall, lignin metabolism has a variety of roles, most notably against numerous environmental stressors, as the plant cell wall serves as the first line of defense against external threats. In this context, their down-regulation suggests that stress might have a negative effect on the growth of these *C. arabica* plants.

### 3.3. Expression of Specific Genes Shaping Heat and Drought Responses in Coffea

Despite the substantial transcriptomic changes reported in the two genotypes, some classes of genes seem to be more relevant to stress tolerance/response in *Coffea* spp., as they were predominantly regulated, namely, those involved in general glycosyltransferase, lipid binding/transfer, and oxidoreductase activities (Tables S3–S8). Nevertheless, we also detected differences in some known stress-specific genes involved mostly in water deficit responses (such as aquaporins and aspartic proteases) or heat responsiveness (such as HSPs, although the level of protein was found to also moderately rise under drought alone [39]; please see below).

Under WW conditions, the increase in temperature to 37 °C triggered the high expression of genes involved in glycosyltransferase activities in Icatu plants, such as the UDP-glycosyltransferase 708C2-like gene, which can facilitate efficient ROS detoxification [55]. An increase in the expression of these genes was further observed in Icatu under the combined action of heat and drought, with the up-regulation of genes involved in glycosyltransferase activities, such as galactinol synthase (*GolS*). *GolS* is the first committed enzyme in the raffinose family oligosaccharide (RFO) synthesis pathway (which acts as an osmoprotectant), and the expression of *GolS* genes has long been implicated in abiotic stress responses, either directly or through combined transcriptional and post-transcriptional control [56]. Likewise, in *Coffea*, other studies have reported that GolS genes are up-regulated under water deficit, salinity, and heat stress in *C. arabica* cv. IAPAR-59, although with some dissimilarities between transcription levels and the accumulation of metabolites [57].

Considering the top annotated DEGs (Tables S3–S8) and the genes with GO terms associated with drought and heat (Table 1, Table 2, Table 3 and Table 4), it is notable that genes associated with lipid binding (WW, 42 °C) and lipid transfer (WW, 37 °C; SWD, 42 °C) (Tables S3, S4 and S9), as well as two acyl-lipid (9-3)-desaturase-like genes (37 °C and REC14, both in WW plants) (Table S11), were up-regulated exclusively in Icatu plants, except for a non-specific lipid-transfer protein gene that was up-regulated in CL153-SWD plants at 42 °C (Table S7). Drought and temperature stresses usually trigger lipid-dependent signaling cascades that affect membrane conformation and the activities of intracellular proteins and metabolites to cope with stress [58]. Lipids act as signal mediators driving stress responses and activating plant defense systems and are intrinsic components of all cellular membranes; thus, they are essential for stabilizing chloroplast membranes under stress [59]. Accordingly, previous studies found that the lipid dynamics of chloroplast membranes, including quantitative and qualitative changes in lipid classes, fatty acids (FAs), and their unsaturation degrees, through de novo synthesis or the modification of pre-existing FAs, are a key feature of the acclimation of *Coffea* spp. plants to stress conditions, namely, as regards temperature changes. This should also include lipid trafficking (associated with lipid-transfer proteins) to allow the transport and integration of newly synthesized lipids into membranes. A great ability to perform such lipid adjustments and mitigate stress impacts stood out in Icatu [60]. In this genotype, with rising temperatures up to 37 °C, relevant membrane remodeling was found, including a great number of newly synthesized FAs and a significant rise in their unsaturation degree, reflecting increases in the preferential synthesis of highly unsaturated C18:3 (linolenic acid) in relation to saturated C16:0 (palmitic acid). This was reverted at 42 °C when a tolerance threshold was crossed [60]. These reports agree, for instance, with the up-regulation of lipid-transfer genes (37 °C in WW conditions; 42 °C under SWD) (Table S9) as well as that of acyl-lipid (9-3)-desaturase-like genes (the latter at 37 °C, but not at 42 °C) in Icatu (Table S11). This genotype was considered to have a great lipid remodeling capability, associated with a reinforced antioxidative system (in response to cold, heat, and drought) that can better protect the double bonds of unsaturated FAs (that increased), which are preferential targets of reactive oxygen species (ROS) [21,42,43,61]. This further agrees with the absence of such gene up-regulation in CL153, which did not show significant changes in the FA level or unsaturation degree at 37 °C (as compared with 25 °C), always under WW conditions [60].

In Icatu plants, an acidic endochitinase was also found to be up-regulated at 42 °C (Table 3 and Table S11). Endochitinase genes are important regulators of abiotic stress, namely, heat, as shown in *Arabidopsis* plants [62]. In *Coffea*, an immunodetection study revealed that class I chitinases are particularly relevant and might participate in the defense responses of plants, at least to coffee rust [63]. Genes involved in oxidoreductase activities were also up-regulated, namely, with the combination of heat and drought, especially in CL153 plants (already at 37 °C), where a high expression of reticuline oxidase genes (Table S6) was reported. ROS-scavenging enzymes such as catalase, thioredoxin, and reticuline oxidase are important for coping with the overproduction and presence of elevated levels of ROS triggered by stress conditions. These (and other) antioxidative molecules would therefore contribute to controlling oxidative damage to DNA, RNA, and a large number of proteins, namely, those associated with photosynthetic functioning [21]. Likewise, in *Coffea* spp., the up-regulation of reticuline oxidase genes has been reported in both CL153 and Icatu plants, even under moderate levels of drought [64].

Cellulose synthase A genes were also found to be up-regulated in CL153 (under WW conditions at 37 °C and REC14 and under SWD at 42 °C). The cellulose synthase superfamily synthesizes cellulose in plant cell walls, an activity highly affected by abiotic factors [65]. At extreme temperatures, the accumulation of cellulose is usually altered in several plants to cope with stress [66], as seemed to also occur here.

Water deprivation and desiccation genes are also major components of stress acclimation in *Coffea* spp. [64,67,68,69,70]. Among them, aquaporins encompass a family of membrane water channels, essential in controlling water transfer into and out of plant cells and, therefore, in maintaining water homeostasis in plants. These molecules are usually over-expressed under drought and were reported to have an important role in controlling the water status in *Coffea* species and genotypes, including *C. arabica* cvs. Catuaí and Mundo Novo, *C. canephora* cv. Apoatã, a graft of Mundo Novo on Apoatã [70], *C. arabica* cv. IAC 44 [71], and *C. arabica* cv. Pacamara [67]. This is also the case in our study since aquaporin DEGs were only recorded in Icatu, but not in CL153. The up-regulation of some aquaporin genes was found in Icatu, even under WW conditions, in response to 37 °C (*PIP2-2*) but especially at 42 °C (*PIP1-1*, *PIP1-2* and *TIP1-3*, *TIP4-1*) (Table 1 and Table S9). Only two aquaporins (*PIP1-2* and *PIP2-2*) were up-regulated in Icatu-SWD plants, but only at 42 °C. This highlights a signal for aquaporin synthesis under these stressful conditions but also shows the stronger impact of the stress combination. Notably, upon the reestablishment of control conditions (REC14), these *PIP* DEGs were no longer detected (under WW) or were shown to be down-regulated (under SWD). Therefore, these findings suggest that timely aquaporin gene expression is involved in the ability to conserve water in Icatu (WW and SWD) plants under heat alone and under heat and drought combined.

Aspartic proteases also play an important role in stress responses in *Coffea*, namely, under drought conditions [64]. For instance, plant proteases are involved in the crosstalk among phytohormones and the adjustment of the stomatal aperture, which is also related to the scavenging of ROS [72]. *ASPG1* is preferentially expressed in the guard cells of *Arabidopsis*, probably functioning under drought through the action of abscisic acid (ABA) signaling in guard cells [73]. In the current study, several aspartic proteases in guard-cell 1 genes (*ASPG1*) were up-regulated in Icatu-WW plants (at both temperatures, Table 1 and Table S9) and were down-regulated in Icatu-SWD plants after stress ended (REC14). They were also up-regulated in CL153-WW plants at 37 °C (but down-regulated with the superimposition of SWD; Table 2 and Table S10), thus likely contributing to overcoming and preventing stress impacts, especially in Icatu-WW plants.

HSPs act as chaperones, ensuring the proper folding of proteins, and are involved in several signaling stress pathways while also playing functional roles in plant development [74]. For instance, HSP21 plays a critical role in the development of the *Arabidopsis* chloroplast under heat stress [75]. During stress, plants often decrease the synthesis of several proteins while transcribing and translating heat shock proteins (HSPs) [76]. Even though the expression of HSPs varies considerably between plants and is genotype-specific, some are up-regulated in resistant cultivars, such as foxtail millet, and down-regulated in sensitive genotypes [76]. CL153 plants reacted to high temperatures (even under WW conditions), up-regulating several heat-responsive genes. These included genes encoding for proteins with known protective roles, namely, chaperone proteins from mitochondria and, particularly, chaperone/chaperonin proteins from chloroplasts (along with the RuBisCO large subunit) and HSPs (such as 18.5 kDa class I, 22.7 kDa class IV, or 18.2 kDa class II gene), at both high temperatures but with some expression rising from 37 to 42 °C (Tables S6, S7 and S12). In contrast, Icatu showed the down-regulation of HSP genes at 37 °C and especially at 42 °C (under either WW or SWD conditions; Table S4). These findings are in disagreement with the substantial up-regulation of the HSP70 gene in other *C. arabica* genotypes (Geisha 3, Marsellesa, and their hybrid), both at 37 °C and especially at 42 °C, when maximum transcript accumulation was observed, decreasing afterward during a recovery period [77]. In this way, species and genotype (within *C. arabica*) dependencies exist regarding the triggering of the HSP gene. Thus, the up-regulation of HSPs in CL153 plants, already at the lower temperature, may indicate a fast response to overcome the effects of heat, while Icatu could have a negative impact associated with the down-regulation of these genes. However, this is not what happens as regards the important HSP70. In fact, Icatu-WW plants showed a similar presence of HSP70 at 37 °C and about twice the amount at 42 °C as compared with CL153-WW plants [39], confirming earlier findings that both genotypes show a response and a large presence of HSP70 from 31 °C and onwards (as compared with 25 °C) [43]. Furthermore, both genotypes also showed a relevant increase in HSP70 content with the single exposure to moderate (MWD) and severe (SWD) water deficits at 25 °C, but with a much greater presence in Icatu-MWD and SWD than in their CL153 counterparts [78]. Additionally, these SWD plants more than doubled their amounts at 31 °C, which cannot be considered a stress (heat) temperature [39], clearly showing that HSP70 synthesis is triggered quite early by heat and/or drought in these two genotypes (greater for Icatu under a single exposure to either of these environmental variables), despite some contrasting gene expression patterns. On the other hand, previous physiological/biochemical studies reported that CL153-SWD plants were unable to show a full recovery of several photosynthetic parameters by REC14, in contrast to Icatu [11,39], with the latter genotype showing even better performance under elevated air [CO_2_] [42,43]. Still, both genotypes clearly showed a decline in physiological/metabolic performance when exposed to 42 °C (regardless of water availability), although Icatu plants displayed greater coordinated protection, membrane lipid profile dynamics, and antioxidant response under these stresses, also reflected in a quite minor change in membrane selectivity under drought and/or heat imposition, even at 42 °C [39]. Thus, since the regulation of some transcriptomic changes does not fully agree with all physiological results, we hypothesize the existence of post-transcriptomic changes in these genotypes, as also highlighted in a previous *Coffea* study [68].

### 3.4. The Role of Transcription Factors (TFs) in Stress Responses

The observed reprogramming of the transcriptome in response to heat and drought involved the differential expression of 145 TFs in Icatu and 65 in CL153. TFs act in the regulation of stress-responsive genes and are responsible for inducing tolerance to several stresses [79]. Accordingly, the TFs reported in this study included many ethylene-responsive, NAC-domain, and MYB TFs, which were up-regulated in Icatu with the increase in temperature and under WW conditions but mostly down-regulated under heat and drought, including MADS-box, auxin response, and general regulatory TF genes (Table S13). By contrast, in CL153, TFs were mostly down-regulated under WW conditions, decreasing in number as the temperature increased, but were up-regulated under the combination of both heat and drought (Table S14). While up-regulated TFs were mostly involved in general responses, down-regulated ones involved regulatory factors and MADS-box genes (Table S14). Previous studies also suggested an important role for TFs in general responses in *Coffea*. For instance, in *C. arabica*, TFs such as *MYB*, *bHLH*, and *NAC* negatively regulate carotenoid biosynthesis [80]. *NAC* TFs also seem to act in different stages of fruit development [81] in several *Coffea* species [51]. ERF TFs were another over-represented family that was identified in this study under single and combined stresses. For instance, DREB, a well-studied ERF in *Coffea* species [64,82], was also found to be over-expressed in this study in response to heat and drought. As the activation of DREB genes induces a set of other abiotic stress-related genes, they are usually important for stress tolerance in plants. Usually, Icatu-WW plants seem to be quite responsive to temperature stress alone, whereas CL153 plants present a greater response to the stress combination. Still, a particularly important finding was associated with the response of MADS-box genes. Although in Icatu-WW plants, there was up-regulation at 37 °C, in CL153 plants (regardless of water availability), we found a down-regulation of most MADS-box genes (except for one up-regulated at 42 °C and REC-SWD plants), suggesting that heat and/or drought might have strong effects on the development of *Coffea* flowers from this *C. canephora* genotype. MADS-box genes are a family of TFs essential for the development of many plants, including *Coffea* and its flowers [83]. For instance, in *C. arabica*, the MADS-box APETALA1 (*AP1*) is expressed in later stages of flower development, being restricted to the perianth, as well as APETALA3 and TM6 orthologs [84]. On the contrary, the PISTILLATA ortholog is widely expressed in the early stages and restricted to stamens and carpels in the later stages of flower development, while the AGAMOUS ortholog is always restricted to fertile organs [84]. MADS-box genes are also widely involved in regulatory networks that modulate plant responses to stress, and changes in their expression due to abiotic changes can affect flower development [85]. Thus, changes in MADS-box genes should be evaluated in future studies focused on the transcriptome of flowers.

## 4. Materials and Methods

### 4.1. Plant Materials and Growth Conditions

The plant material and the experimental design followed previously published procedures [11,39]. A total of 32 plants from the two main produced *Coffea* species, *C. canephora* cv. Conilon Clone 153 (CL153) and *C. arabica* cv. Icatu Vermelho (Icatu), were used. CL153 is a late-maturation/ripening diploid clonal variety created from Emcapa 8131 (also known as Vitória 13) by Incaper (Vitória, ES, Brazil) that shows some relevant drought tolerance, while Icatu is an introgressed tetraploid variety originated from a cross between *C. canephora* and *C. arabica* cv. Bourbon Vermelho that was further crossed with *C. arabica* cv. Mundo Novo in IAC, Brazil. These plants were grown from the seedling stage, for seven years, in 80 L pots in walk-in growth chambers (EHHF 10000, ARALAB, Albarraque, Portugal) under controlled conditions of temperature (25/20 °C, day/night, ±1 °C), irradiance (max. approx. 750 μmol m^−2^ s^−1^ at the upper part of the plant, using a combination of fluorescent, metal halide, and halogen lamps to provide a balanced light spectrum), relative humidity (70 ± 2%), photoperiod (12 h), and ambient [CO_2_] (380 ± 5 μL L^−1^). Plants were maintained without restrictions on nutrients (with fertilization following [86]) or water availability before water deficit and high-temperature conditions were gradually imposed (to allow plant acclimation).

### 4.2. Drought and Temperature Stress Implementation

At the control temperature (25/20 °C, day/night), for well-watered (WW) plants, adequate soil moisture was maintained by watering them every two days, whereas a second set of plants were subjected to partial irrigation withholding to impose severe water deficit (SWD) conditions as an individual stressor; these conditions represented approx. 80% (WW) or 10% (SWD) of maximum pot water availability. SWD status was reached after two weeks, when predawn water potential (Ψpd) values were below −3.7 MPa. The WW plants maintained Ψpd values above −0.35 MPa. After an additional period of 5 days (to stabilize these water availability conditions), the gradual temperature rise started (see below).

The temperature rise was imposed on WW and SWD plants at a rate of 0.5 °C day^−1^ (diurnal temperature), with 5 days of stabilization at 31/25 °C, 37/28 °C (37 °C plants), and 42/30 °C (42 °C plants), when sampling on recently matured leaves was performed. Afterward, control conditions were reestablished (25/20 °C; WW), and the plants were monitored over a recovery period of 14 days (REC14).

It is noteworthy to mention that SWD plants reached the desired water status within 14 days, followed by a 5-day Ψpd stabilization (always under 25/20 °C), before a gradual rise in temperature to 42/30 °C (which lasted for 49 days), facing water shortage for 68 days in an experiment that lasted in total 82 days.

### 4.3. RNA Extraction and Illumina Sequencing

Newly matured leaves from plagiotropic and orthotropic branches from well-illuminated plants (upper third part of the canopy) were collected under photosynthetic steady-state conditions after 2 h of illumination, flash-frozen in liquid N2, and stored at −80 °C. Total RNA was extracted following [44] using the Analytik-Jena InnuSPEED Plant RNA Kit (Analytik Jena Innuscreen GmbH, Jena, Germany). A total of 48 samples [2 genotypes × 2 water treatments × 4 temperatures (25 °C, 37 °C, 42 °C, and REC14 plants) × 3 individual plants] were assessed. RNA quantity and quality were determined using a BioDrop Cuvette (BioDrop, Nottingham, UK) and an Agilent 2100 Bioanalyzer (Agilent Technologies, Santa Clara, CA, USA). The RNA integrity number (RIN) for the samples ranged from 8.99 to 9.15. The mRNA libraries were constructed with the Illumina TruSeq Stranded mRNA Sample Preparation kit (Illumina, San Diego, CA, USA) and sequenced on an Illumina NovaSeq6000 at Macrogen facilities (Macrogen, Geumcheongu, Seoul, Republic of Korea).

### 4.4. Quality Control Analysis of Sequencing Data

Raw reads’ quality was assessed using FastQC version 0.11.9 [87] to check for low-quality reads. FastQ Screen version 0.14.0 [88] was used to check for contaminants against the genome of the most common model organisms (e.g., *Homo sapiens*, *Mus musculus*, *Rattus norvegicus*, *Drosophila melanogaster*, *Caenorhabditis elegans*, *Saccharomyces cerevisiae*, and *Escherichia coli*) and adapter databases (e.g., Mitochondria RNA, PhiX, Vector from UniVec database, FastQ Screen rRNA custom database, and FastQ Screen Adapters database). Due to the overall good quality of the reads, the trimming step was skipped, since trimming can be redundant in differential expression analysis, as RNA-Seq aligners can remove adapter sequences through soft-clipping, which rescues low-sequencing-quality bases and improves the accuracy of the analysis [89].

### 4.5. Reference-Based Mapping and Gene Expression Quantification

STAR version 2.7.9a [90] was used to align reads against the respective reference genomes. Icatu reads were mapped onto the *C. arabica* genome, downloaded from NCBI (https://www.ncbi.nlm.nih.gov/assembly/GCF_003713225.1, accessed on 10 November 2021). CL153 reads were mapped onto the *C. canephora* genome, downloaded from the *Coffea* Genome Hub (http://coffee-genome.org/download, accessed on 10 November 2021) [91]. Then, Htseq-count version 0.13.5 [92] was used to count uniquely mapped genes, determining gene expression. Samtools version 1.14 [93] was used to obtain the general statistics of the genome mapping.

### 4.6. Detection of Differentially Expressed Genes (DEGs)

A Principal Component Analysis (PCA) was performed on the expression data of genes using the DESeq2 median of ratio-normalized gene counts, and rlog transformation, using the ggplot2 version 3.3.5 library [94] of R software version 4.0.2 [95]. To identify outliers, we used the PcaGrid function from the rrcov version 1.6.2 package, which excluded the replicate 7B from downstream analysis. Both DESeq2 version 1.28.1 [96] and edgeR-robust version 3.32.1 [97] were used to identify differentially expressed genes (DEGs) between treatments, with Benjamini and Hochberg’s approach being used to control the false discovery rate (FDR) [98]. DEGs commonly found by DESeq2 and edgeR were defined as genes with a normalized non-zero log2 fold change (FC) expression and an FDR < 0.01. Up- and down-regulated DEGs were reported based on comparisons with control plants (WW, 25 °C), considering both genotypes. Python’s matplotlib library was used to plot Venn diagrams and bar plots [99], while ggplot2 from R was used to plot heatmaps with dendrograms to visualize DEGs based on the differential expression patterns between the different treatments. To prevent highly differentially expressed genes from clustering together without considering their expression patterns, log2 fold change was scaled by gene across treatments (row Z-score).

### 4.7. Functional Annotation of DEGs

The functional annotations obtained from the above-mentioned *C. arabica* and *C. canephora* genomes were used to annotate the responsive DEGs of Icatu and CL153, respectively. Specific searches were also performed targeting water- and temperature-related DEGs, namely, those associated with energy metabolism and acclimation responses, which were previously found to be important for *Coffea* resilience expressed at physiological and biochemical levels [11,19,39,42,43]. The following annotations were therefore considered: “water transport” (GO:0006833), “water homeostasis” (GO:0030104), “response to water” (GO:0009415), “response to water deprivation” (GO:0009414), and “response to desiccation” (GO:0009269) [Drought], “temperature homeostasis” (GO:0001659), “response to temperature stimulus” (GO:0009266), “heat shock protein binding” (GO:0031072), “protein refolding” (GO:0042026), “modulation by symbiont of host response to temperature stimulus“ (GO:0033636), “response to unfolded protein” (GO:0006986), and “temperature-gated ion channel activity” (GO:0097603) [Heat]. To understand the role of transcription factors (TFs), DEGs annotated with the GO terms “DNA-binding transcription factor activity” (GO:0003700) and/or “general transcription initiation factor binding” (GO:0140296) were also filtered, and their regulation patterns were analyzed. Finally, the involvement of DEGs in key physiological and biochemical *Coffea* processes was sought to study their regulation patterns (e.g., antioxidant activity, lipid metabolism, photosynthesis, and respiration) following [44].

### 4.8. Enriched Gene Ontology (GO) Terms

An over-representation analysis (ORA) was performed using gProfiler [100] with an FDR < 0.01 to identify enriched Gene Ontology (GO) terms and the functional classification of DEGs. Afterward, REVIGO [101] was used to remove redundancy with a similarity cutoff = 0.5 and a gene count cutoff = 10, plotting enriched non-redundant data with ggplot2.

### 4.9. Quantitative RT-PCR

Nine transcripts were randomly selected for real-time quantitative PCR (qRT-PCR) to verify the accuracy of the levels of expression obtained by RNA-seq. These genes included *PP2C-51*: protein phosphatase 2C 51-like (LOC113703008); *LEA-DC3*: late embryogenesis abundant protein Dc3-like (LOC113740436); *DH1a*: dehydrin DH1a (Cc07_g10030); *SUS2*: sucrose synthase 2-like (LOC113735267); *PIP2-2*: aquaporin PIP2-2-like (LOC113707992); *XTH6*: xyloglucan endotransglucosylase/hydrolase protein 6 (Cc07_g07560); *GOLS2*: galactinol synthase 2-like (LOC113727829); *CuSOD1*: Superoxide dismutase [Cu-Zn] (LOC113727514); and APX_Chl_: chloroplast ascorbate peroxidase (LOC113728228). All primer sequences are presented in Table S17. Primer3 web version 4.1.0 [102] was used to design the primers with an e-value < 2 × 10^−4^ and a score > 41. cDNA was synthesized from 1 μg of total RNA using the SensiFASTTM cDNA Synthesis kit (Meridian BioScience, Cincinnati, OH, USA), according to the manufacturer’s recommendations. PCR reactions were prepared using the SensiFASTTM SYBR No-ROX kit (Meridian BioScience, USA) following the protocol and the parameters described in [68]. Correlation analyses were performed to understand the level of agreement between expression levels obtained from qRT-PCR and those from RNA sequencing.

## 5. Conclusions and Future Perspectives

This study provides a holistic view of transcriptomic responses triggered by heat with and without drought co-occurrence in two *Coffea* genotypes. The results revealed that transcriptomic differences under the combined action of drought and heat stress are not a simple additive pattern of the two individual stresses. By contrast, new transcripts were triggered by the combination of these factors, revealing harsher effects. In addition, the results were also highly genotype-dependent. For instance, aquaporins that are usually over-expressed under drought in several *Coffea* species were only recorded in Icatu, but not in CL153. Their up-regulation, even under WW conditions, in response to temperature increases suggests a stronger ability of Icatu to deal with water scarcity than CL153. Based on these transcriptomic results and supported by previous physiological findings, the resilience of these elite genotypes can be achieved by several molecular mechanisms since we found the expression of different but important heat- and drought-responsive genes in the two genotypes.

Advances in conventional and molecular breeding and the introduction of tolerant genotypes into existing production systems have the potential to accelerate breeding progress and create resilient varieties that can withstand stress. Thus, the information from this study not only allows us to understand how *Coffea* plants perceive different abiotic stresses but also reveals the importance of incorporating these elite genotypes in targeted breeding to improve heat and drought tolerance in Coffea species.

Finally, we advocate for more genomic studies combining different stress conditions, focusing on different species, genotypes, and organs. Specifically, our results highlight the knockdown of several MADS-box genes under heat and drought, especially in the studied *C. canephora* genotype. For instance, TFs were up-regulated in Icatu under temperature stress (under WW conditions) but down-regulated in CL153 and decreased under heat stress, while under the stress combination, they were down-regulated in Icatu but up-regulated in CL153. This indicates the need for specific studies on *Coffea* flowers since these changes in the gene expression of TFs might have important consequences for flower development and, ultimately, for the quality and quantity of coffee beans.

## Figures and Tables

**Table 1:** Most up- and down- regulated DEGs involved in drought responses under WW conditions or after gradual exposure to SWD and after temperature increases to 37 °C and 42 °C in Icatu plants.

WW	**Protein name**	**37 °C**	**Protein name**	**42 °C**	**Protein name**	**REC14**
	Non-specific lipid-transfer protein 1-like	12.18	Annexin D4-like	10.06	NAC domain-containing protein 72-like	**2.47**
	AAA-atpase ASD	10.60	Probable aquaporin PIP1-2	5.63	NAC domain-containing protein 72-like	**3.08**
	Acidic endochitinase-like	9.38	Squalene monooxygenase-like	4.36		
	Wall-associated receptor kinase 2-like	8.54	Basic endochitinase-like	4.29		
	Protein NLP1-like isoform X1	8.49	Aquaporin TIP4-1-like	4.06		
	Homeobox-leucine zipper protein ATHB-7-like	−3.29	AAA-atpase ASD	−3.30		
	LEA protein Dc3-like	−3.50	Endoplasmin homolog	−3.37		
	UDP-glycosyltransferase 74E2-like	−3.63	WRKY DNA-binding transcription factor 70-like	−3.63		
	Zinc finger protein ZAT10-like	−4.05	F-box/LRR-repeat MAX2 homolog A-like	−3.70		
	F-box/LRR-repeat MAX2 homolog A-like	−4.58	Zinc finger protein ZAT10-like	−4.25		
SWD	**Protein name**	**37 °C**	**Protein name**	**42 °C**	**Protein name**	**REC14**
	Protein phosphatase 2C 51-like	4.66	Non-specific lipid-transfer protein 1-like	8.82	Galactinol synthase 2-like	7.90
	Acidic endochitinase SE2-like	3.40	Annexin D4-like	5.22	Galactinol synthase 2-like	7.23
	Calmodulin-binding protein 25-like	2.70	Probable aquaporin PIP1-2	4.70	Galactinol synthase 2-like	5.52
			Protein Aspartic protease in guard cell 1-like	3.95	Homeobox-leucine zipper protein ATHB-12-like	5.24
			Aquaporin PIP2-2-like	3.47	Protein phosphatase 2C 51-like	5.16
	Chlorophyll a-b binding protein 36	−2.81	Endoplasmin homolog	−1.84	Aspartic proteinase nepenthesin-1-like	−6.86
					Ethylene-responsive transcription factor WIN1-like	−7.45
					Basic endochitinase-like	−8.11
					Protein Aspartic protease in guard cell 1-like	−8.61
					Xyloglucan endotransglucosylase/hydrolase protein 6	−10.98

**Table 2:** Most up- and down-regulated DEGs involved in drought responses under WWSWD conditions and after temperature increases to 37 °C and 42 °C in CL153 plants.

WW	**Protein name**	**37 °C**	**Protein name**	**42 °C**	**Protein name**	**REC14**
	Laccase-2	5.17			Cellulose synthase A catalytic subunit 8 [UDP-forming]	5.60
	Cellulose synthase A catalytic subunit 8 [UDP-forming]	5.12				
	Multiprotein-bridging factor 1c	3.64				
	Protein aspartic protease in guard cell 1	2.08				
	Binding	1.17				
			Histidine kinase 4	−2.34		
			Mitogen-activated protein kinase 3	−1.90		
SWD	**Protein name**	**37 °C**	**Protein name**	**42 °C**	**Protein name**	**REC14**
	Putative Protein aspartic protease in guard cell 1	4.74	Cellulose synthase A catalytic subunit 8 [UDP-forming]	7.11	Xyloglucan endotransglucosylase/hydrolase protein 6	5.30
	Mitogen-activated protein kinase 3	1.72	Multiprotein-bridging factor 1c	5.02	Putative Protein aspartic protease in guard cell 1	4.89
					Protein aspartic protease in guard cell 1	4.74
					Putative Protein aspartic protease in guard cell 1	3.24
					LEA hydroxyproline-rich glycoprotein family	3.01
	Putative overexpressor of cationic peroxidase 3	−2.89	Mitogen-activated protein kinase 3	−2.27	Dehydrin DH1a	−3.59
	Multiprotein-bridging factor 1c	−3.30	Putative Protein aspartic protease in guard cell 1	−3.65	Putative Protein aspartic protease in guard cell 1	−3.72
			Putative Protein aspartic protease in guard cell 1	−3.03	Putative Protein aspartic protease in guard cell 1	−4.31
			18 kDa seed maturation protein	−5.48	Putative Protein abscisic acid-insensitive 5	−4.77

**Table 3:** Most up- and donw-regulated DEGs related to heat stress under WW conditions or after gradual exposure to SWD and after temperature increases to 37 °C and 42 °C in Icatu plants.

WW	**Protein name**	**37 °C**	**Protein name**	**42 °C**	**Protein name**	**REC14**
	Auxin-binding protein ABP20-like	12.41	Uncharacterized protein LOC113695405	8.28	Annexin D4-like	10.06
	AAA-atpase ASD	10.60	Acidic endochitinase SE2-like	3.40	Protein NDL2-like	5.53
	Acidic endochitinase-like	9.38			3-ketoacyl-coa synthase 1	5.24
	14 kda proline-rich protein DC2.15-like	8.61			14 kda proline-rich protein DC2.15-like	5.16
	PLAT domain-containing protein 3-like	8.33			3-ketoacyl-coa synthase 6-like	4.59
	17.5 kDa class I HSP-like	−8.17	18.5 kda class I heat shock protein-like	−5.48	Class I HSP-like	−10.60
	Class I HSP-like	−8.84	18.2 kda class I HSP-like	−6.00	18.5 kda class I heat shock protein-like	−11.11
	Heat shock 70 kda protein-like	−10.37	17.5 kDa class I HSP-like	−6.37	Small HSP	−11.25
	Small HSP	−10.42	Small HSP	−7.68	Heat shock 70 kda protein-like	−11.85
	22.0 kda class IV heat shock protein-like	−14.90	22.0 kda class IV heat shock protein-like	−9.19	22.0 kda class IV heat shock protein-like	−15.50
SWD	**Protein name**	**37 °C**	**Protein name**	**42 °C**	**Protein name**	**REC14**
	Acidic endochitinase-like	2.63	Xyloglucan endotransglucosylase/hydrolase protein 22-like	2.55	Galactinol synthase 2-like	5.52
	3-ketoacyl-coa synthase 6-like	3.83			Peroxidase 3-like	5.03
	MYB-related protein 306-like	2.91			Galactinol synthase 2-like	7.90
	3-ketoacyl-coa synthase 1	4.03			Galactinol synthase 2-like	7.23
	Annexin D4-like	5.22			Heat shock 70 kda protein-like	5.05
	BAG family molecular chaperone regulator 6-like	−7.42			Basic endochitinase-like	−6.79
	17.5 kDa class I HSP-like	−8.17			Auxin-binding protein ABP20-like	−7.42
	18.2 kda class I HSP-like	−8.06			3-ketoacyl-coa synthase 19-like	−7.48
	Small HSP	−10.42			Basic endochitinase-like	−8.11
	22.0 kda class IV heat shock protein-like	−14.90			36.4 kda proline-rich protein-like	−9.06

**Table 4:** Most up- and down-regulated DEGs related to heat stress under WW or after gradual exposure to SWD and after temperature increases to 37 °C and 42 °C in CL153 plants.

WW	**Protein name**	**37 °C**	**Protein name**	**42 °C**	**Protein name**	**REC14**
	Plant protein of unknown function (DUF828)	3.79	DnaJ protein homolog ANJ1	4.55	Pollen-specific protein SF21	2.80
	Multiprotein-bridging factor 1c	3.64	Aldolase-type TIM barrel family protein	4.38		
	Heat shock 70 kDa protein 8	3.55	15.7 kDa heat shock protein	4.45		
	15.7 kDa heat shock protein	3.11	Putative Activator of 90 kDa heat shock protein ATPase homolog	4.93		
	Aldolase-type TIM barrel family protein	2.93	Multiprotein-bridging factor 1c	5.72		
	CBL-interacting serine/threonine-protein kinase 7	−1.13	Mitogen-activated protein kinase 3	−1.90		
SWD	**Protein name**	**37 °C**	**Protein name**	**42 °C**	**Protein name**	**REC14**
	Mitogen-activated protein kinase 3	1.72	15.7 kDa heat shock protein	5.05	ATP synthase delta chain	2.51
	Arginine decarboxylase	1.72	Multiprotein-bridging factor 1c	5.02	Unknown protein DS12 from 2D-PAGE of leaf	2.48
			Heat shock 70 kDa protein 8	3.66	3-ketoacyl-CoA synthase 10	2.44
			Putative Activator of 90 kDa heat shock protein ATPase homolog	3.53	Alpha-glucan water dikinase	1.75
			20 kDa chaperonin	2.76		
	RuBisCO large subunit-binding protein subunit beta	−2.73	Mitogen-activated protein kinase 3	−2.27	Putative Protein abscisic acid-insensitive 5	−4.77
	Ribulose bisphosphate carboxylase/oxygenase activase 1	−2.81	18 kDa seed maturation protein	−5.48		
	Multiprotein-bridging factor 1c	−3.30				
	Heat shock 70 kDa protein 8	−3.95				
	15.7 kDa heat shock protein	−4.99				

## Data Availability

Raw reads have been deposited in the NCBI Sequence Read Archive, BioProject accessions PRJNA787748, PRJNA1087442, PRJNA1087679, PRJNA1088119, and PRJNA1135679.

## Supplementary Materials

The following supporting information can be downloaded at https://www.mdpi.com/article/10.3390/ijms25147995/s1.
