# Supplementary material for: Transcriptomic Analyses Reveal That Coffea arabica and Coffea canephora Have More Complex Responses under Combined Heat and Drought than under Individual Stressors
Source: Int J Mol Sci. 2024 Jul 22;25(14):7995. doi: 10.3390/ijms25147995 (PMC11277005; doi:10.3390/ijms25147995)
Supplement: Supplementary file 1 [file ijms-25-07995-s001.zip › ijms-3108594-supplementary.pdf]

Table S1. Summary of sequencing and mapping of reads from Icatu and CL153 samples.

Genotype	Water	Temperature [ºC]	ID Replicates	Raw reads	Uniquely mapped reads	% Unique	Multiple mapped reads	% Multiple	Unmapped reads	% Unmapped
Icatu	WW	25	**7A**	26528307	19086103	71.95	4741673	17.87	2700531	10.18
			**7B**	22009479	15388127	69.92	3815138	17.34	2806214	12.75
			**7C**	18666431	13878995	74.35	3679294	19.71	1108142	5.93
		37	**31A**	20659232	15785740	76.41	3489521	16.89	1383971	6.70
			**31B**	25908691	20058711	77.42	4143355	15.99	1706625	6.59
			**31C**	22342505	17224336	77.09	3847129	17.22	1271040	5.69
		42	**43A**	20769636	14923493	71.85	4633414	22.31	1212729	5.84
			**43B**	21244486	15470783	72.82	3252059	15.31	2521644	11.87
			**43C**	23595372	16391514	69.47	5098250	21.60	2105608	8.92
		REC14	**55A**	20778352	14697191	70.73	4731740	22.77	1349421	6.50
			**55C**	25434525	17851573	70.19	6173702	24.28	1409250	5.54
			**55D**	21307182	15239722	71.52	4842168	22.73	1225292	5.75
	SWD	25	**9B**	19857280	13851508	69.76	3456848	17.41	2548924	12.84
			**9C**	21199998	15242026	71.90	3068556	14.48	2889416	13.63
			**9E**	34865625	28112421	80.63	4534257	13.00	2218947	6.36
		37	**32D**	22534610	15060176	66.83	4878461	21.65	2595973	11.52
			**33A**	26480603	21042674	79.46	3755743	14.19	1682186	6.35
			**33D**	18185237	12718547	69.94	3769208	20.72	1697482	9.34
		42	**45A**	18395538	12862537	69.92	2942854	15.99	2590147	14.08
			**45C**	20624508	16467333	79.84	2376743	11.52	1780432	8.64
			**45E**	21032009	14121682	67.14	4220110	20.06	2690217	12.79

		**REC14**	**57A**	26882675	18512136	68.86	5937788	22.09	2432751	9.05
			**57D**	17434057	12408543	71.17	3976529	22.81	1048985	6.01
			**57E**	21631206	15405668	71.22	4906447	22.68	1319091	6.10
		**Average**		22431981	16325064	72.52	4177957	18.78	1928959	8.71
**CL153**	**WW**	**25**	**1A**	21337820	18260092	85.58	1039061	4.87	2038667	9.56
			**1B**	19886216	17616010	88.58	552957	2.78	1717249	8.63
			**1C**	23700779	20207509	85.26	930148	3.92	2563122	10.82
		**37**	**25A**	23157593	20662190	89.22	915461	3.95	1579942	6.82
			**25B**	19354075	15802471	81.65	803878	4.15	2747726	14.2
			**25C**	22330166	19597205	87.76	763729	3.42	1969232	8.82
		**42**	**37A**	16635513	12846907	77.23	759913	4.56	3028693	18.2
			**37B**	21151430	18846250	89.10	766360	3.62	1538820	7.27
			**37C**	21166941	18544887	87.61	823810	3.89	1798244	8.50
		**REC14**	**49A**	26566229	21832019	82.18	1627237	6.12	3106973	11.69
			**49B**	19979216	16606002	83.12	1135411	5.69	2237803	11.20
			**49C**	20950821	18603860	88.80	679489	3.24	1667472	7.96
	**SWD**	**25**	**3A**	21944385	20245494	92.26	652290	2.97	1046601	4.77
			**3B**	25523224	22602789	88.56	996514	3.9	1923921	7.54
			**3C**	21988087	17511012	79.64	830372	3.78	3646703	16.58
		**37**	**26D**	21060416	16680351	79.20	1034651	4.92	3345414	15.88
			**27B**	19594738	16996372	86.74	608152	3.1	1990214	10.16
			**27C**	21589460	17197532	79.66	1004204	4.66	3387724	15.69
		**42**	**39A**	16634429	13442636	80.81	701830	4.22	2489963	14.96
			**39B**	17338479	14460570	83.40	874824	5.04	2003085	11.55
			**39D**	22543647	17982642	79.77	1260494	5.60	3300511	14.64

	**51A**	21429606	17106612	79.83	1373145	6.41	2949849	13.76
**REC14**	**51B**	20137340	16901596	83.93	1652355	8.21	1583389	7.86
	**51C**	20591557	18519816	89.94	689944	3.35	1381797	6.71
**Average**		21108007	17878034	84.58	936510	4.43	2293463	10.99

Raw reads: number of reads obtained after sequencing. Uniquely mapped reads: number of reads aligned to a unique position. Multiple mapped reads: number of reads aligned to exons of several overlapping genes. Unmapped reads: number of non-aligning reads [% represents the proportion of the respective number of reads compared to the number of raw reads].

Table S2. Number of DEGs in Icatu and CL153 samples detected by DESeq2 or edgeR-robust.

Genotype	Water	Temperature	DESeq2	edgeR-robust	Total	Up	Down
Icatu	WW	37	7298	8077	6743	4784	1959
		42	4061	4366	3563	1974	1589
		REC14	22	32	20	14	6
	SWD	37	427	1400	377	109	268
		42	908	1118	840	452	388
		REC14	4408	4931	4038	1384	2654
CL153	WW	37	1265	1295	1017	553	464
		42	1099	905	768	366	402
		REC14	331	362	276	171	105
	SWD	37	1604	2421	1513	587	926

42	751	910	625	359	266
REC14	1936	2402	1808	1450	358

Table S3. Top up- and down-regulated annotated DEGs in Icatu plants at 37ºC vs. 25 ºC, under WW or SWD conditions.

**Gene ID**	**Protein Name**	**Molecular Functions**	**Log_2FC**
		**WW**	
		**Up-DEGs**	
LOC113736957	UDP-glycosyltransferase 708C2-like	glycosyltransferase activity	13.15
LOC113688151	GDSL esterase/lipase At2g04570-like	hydrolase activity	12.67
LOC113694491	auxin-binding protein ABP20-like	ion binding	12.41
LOC113701950	leucine-rich repeat extensin-like protein 3		12.34
LOC113737726	UDP-glycosyltransferase 13-like	glycosyltransferase activity	12.24
LOC113731075	non-specific lipid-transfer protein 1-like	lipid binding	12.18
LOC113709624	probable inorganic phosphate transporter 1-9	transporter activity	11.94
LOC113730253	expansin-A4-like		11.72
LOC113711077	nitrate reductase [NADH	oxidoreductase activity; ion binding	11.57
LOC113704390	50S ribosomal protein L19-1, chloroplastic-like	structural constituent of ribosome	11.34
		**Down-DEGs**	

LOC113715627	LOW QUALITY PROTEIN: cell division control protein 48 homolog B-like	ATP binding; hydrolase activity	-10.99
LOC113707349	17.3 kDa class II heat shock protein-like		-11.24
LOC113733718	UDP-glycosyltransferase 74E1-like	glycosyltransferase activity	-11.25
LOC113711848	purine permease 1-like	transporter activity	-11.26
LOC113739782	methylecgonone reductase-like isoform X1	oxidoreductase activity	-11.33
LOC113688954	anthocyanidin 3-O-glucosyltransferase 2-like	glycosyltransferase activity	-11.36
LOC113727653	luminal-binding protein 5-like	ATP hydrolysis activity; ATP binding	-11.52
LOC113706995	17.3 kDa class II heat shock protein-like		-12.68
LOC113706457	caffeic acid 3-O-methyltransferase-like	methyltransferase activity; protein binding	-13.93
LOC113731964	22.0 kDa class IV heat shock protein-like		-14.90
	**SWD**		
	**Up-DEGs**		
LOC113709366	exocyst complex component EXO70H1-like		7.38
LOC113715419	F-box protein At2g02240-like		6.31
LOC113709337	probable WRKY transcription factor 31	transcription regulator activity; DNA binding	6.24
LOC113712224	protein DMP6-like		6.10
LOC113733001	galactinol synthase 2-like isoform X2	glycosyltransferase activity	5.90
LOC113716423	thaumatin-like protein		5.88
LOC113709384	small ubiquitin-related modifier 1-like		5.81

LOC113736835	L-type lectin-domain containing receptor kinase IV.1-like	ATP binding; kinase activity; carbohydrate binding	5.80
LOC113730933	S-linalool synthase-like	ion binding; lyase activity	5.60
LOC113709442	putative 12-oxophytodienoate reductase 11	oxidoreductase activity; ion binding	5.57
	**Down-DEGs**		
LOC113713893	putative 12-oxophytodienoate reductase 11	oxidoreductase activity; ion binding	-9.15
LOC113731964	22.0 kDa class IV heat shock protein-like		-9.19
LOC113690673	acylsugar acyltransferase 3-like	acyltransferase activity	-9.19
LOC113690639	acidic endochitinase-like	hydrolase activity	-9.77
LOC113711525	probable serine/threonine-protein kinase PIX13	kinase activity; ATP binding	-9.79
LOC113739782	methylecgonone reductase-like isoform X1		-10.24
LOC113734830	CYC02 protein-like	oxidoreductase activity	-10.80
LOC113706481	bifunctional pinoresinol-lariciresinol reductase 2-like		-11.83
LOC113707349	17.3 kDa class II heat shock protein-like		-13.54
LOC113706457	caffeic acid 3-O-methyltransferase-like	methyltransferase activity; protein binding	-14.51

Table S4. Top up- and down-regulated annotated DEGs in Icatu plants at 42 ºC vs. 25 ºC, under either WW or SWD conditions.

Gene ID	Protein Name	Molecular Functions	Log_2FC
		**WW**	
		**Up-DEGs**	
LOC113730171	21 kDa protein-like	enzyme inhibitor activity	13.39
LOC113725144	21 kDa protein-like	enzyme inhibitor activity	11.27
LOC113726198	transcription factor MYB80-like isoform X2		11.06
LOC113732511	annexin D4-like	lipid binding; ion binding	10.06
LOC113741845	transcription factor MYB4-like isoform X1		9.96
LOC113736957	UDP-glycosyltransferase 708C2-like	glycosyltransferase activity	9.77
LOC113706592	myb-related protein Zm38-like		9.73
LOC113732826	hyoscyamine 6-dioxygenase-like	oxidoreductase activity; ion binding	9.71
LOC113713847	uncharacterized protein LOC113713847		9.44
LOC113737726	UDP-glycosyltransferase 13-like	glycosyltransferase activity	9.40
		**Down-DEGs**	
LOC113706638	tabersonine 16-hydroxylase 1-like	oxidoreductase activity; ion binding	-11.05
LOC113717780	18.5 kDa class I heat shock protein-like		-11.11
LOC113718871	small heat shock protein, chloroplastic-like	response to heat	-11.25

LOC113739647	heat shock 70 kDa protein-like	ATP binding; hydrolase activity	-11.85
LOC113707349	17.3 kDa class II heat shock protein-like		-12.06
LOC113728093	17.4 kDa class I heat shock protein-like		-12.61
LOC113727653	luminal-binding protein 5-like	ATP binding; hydrolase activity	-13.08
LOC113706995	17.3 kDa class II heat shock protein-like		-13.94
LOC113706996	17.3 kDa class II heat shock protein-like		-14.20
LOC113731964	22.0 kDa class IV heat shock protein-like		-15.50
	**SWD**		
	**Up-DEGs**		
LOC113731075	non-specific lipid-transfer protein 1-like	lipid binding	8.82
LOC113737846	glycine-rich cell wall structural protein-like		7.08
LOC113731024	protein CHLOROPLAST IMPORT APPARATUS 2-like		7.04
LOC113692720	defensin-like protein P322		7.02
LOC113717150	thioredoxin H2-like		6.93
LOC113716940	phosphate transporter PHO1 homolog 1 isoform X2		6.45
LOC113694625	gamma-glutamyl peptidase 5-like		6.45
LOC113734418	chalcone synthase 2-like	acyltransferase activity	6.30
LOC113717245	thioredoxin H2-like	oxidoreductase activity	6.21
LOC113714862	21 kDa seed protein-like	enzyme inhibitor activity	6.17

**Down-DEGs**			
LOC113706995	17.3 kDa class II heat shock protein-like		-7.85
LOC113691110	tyrosine aminotransferase-like	transaminase activity; ion binding	-7.89
LOC113716352	18.5 kDa class I heat shock protein-like		-8.02
LOC113703359	small heat shock protein. chloroplastic-like		-8.18
LOC113687832	probable aminotransferase TAT2	transaminase activity; ion binding	-8.28
LOC113689825	tabersonine 16-hydroxylase 1-like	oxidoreductase activity; ion binding	-8.34
LOC113731964	22.0 kDa class IV heat shock protein-like		-8.72
LOC113706996	17.3 kDa class II heat shock protein-like		-8.87
LOC113728093	17.4 kDa class I heat shock protein-like		-9.14
LOC113707349	17.3 kDa class II heat shock protein-like		-11.85

Table S5. Top up- and down-regulated annotated DEGs in Icatu plants at REC14 vs. 25 ºC, under either WW or SWD conditions.

Gene ID	Protein Name	Main Functions	Log_2FC
		**WW**	
		**Up-DEGs**	
LOC113689826	NAC domain-containing protein 72-like	DNA binding	3.08
LOC113692724	protein STRICTOSIDINE SYNTHASE-LIKE 2-like		2.85
LOC113691978	xyloglucan endotransglucosylase/hydrolase protein 22-like	glycosyltransferase activity; hydrolase activity	2.55
LOC113718214	probable xyloglucan endotransglucosylase/hydrolase protein 33	glycosyltransferase activity; hydrolase activity	2.48
LOC113743599	NAC domain-containing protein 72-like	DNA binding	2.47
LOC113688537	cysteine-rich and transmembrane domain-containing protein WIH1-like		1.76
		**Down-DEGs**	
LOC113717414	two-component response regulator ARR6-like		-1.94
LOC113732962	nicotianamine synthase-like	transferase activity	-2.42
LOC113742222	protein HOTHEAD	FAD binding; oxidoreductase activity	-3.27
LOC113692565	probable carboxylesterase 15	hydrolase activity	-4.14
		**SWD**	
		**Up-DEGs**	
LOC113713504	protein DMP6-like		9.25

LOC113733000	galactinol synthase 2-like	glycosyltransferase activity	7.90
LOC113741877	probable E3 ubiquitin-protein ligase ATL44		7.54
LOC113706185	expansin-like B1		7.49
LOC113727830	galactinol synthase 2-like	glycosyltransferase activity	7.23
LOC113698524	protein IQ-DOMAIN 1-like	calmodulin binding	7.09
LOC113706427	expansin-like B1		7.02
LOC113692130	early nodulin-like protein 2	oxidoreductase activity	6.66
LOC113736293	cellulose synthase-like protein D1	glycosyltransferase activity	6.60
LOC113700219	desiccation-related protein clone PCC6-19-like		6.57
**Down-DEGs**			
LOC113714314	germin-like protein subfamily 1 member 7	ion binding	-9.75
LOC113695211	probable beta-1,4-xylosyltransferase IRX9	glycosyltransferase activity	-9.90
LOC113736409	xyloglucan endotransglucosylase/hydrolase 2-like	glycosyltransferase activity; hydrolase activity	-10.01
LOC113714995	subtilisin-like protease SBT1,9	hydrolase activity	-10.15
LOC113688151	GDSL esterase/lipase At2g04570-like	hydrolase activity	-10.39
LOC113734704	probably inactive leucine-rich repeat receptor-like protein kinase At3g28040	ATP binding; kinase activity	-10.49
LOC113708131	fasciclin-like arabinogalactan protein 12		-10.71
LOC113697821	probable xyloglucan endotransglucosylase/hydrolase protein 6	glycosyltransferase activity; hydrolase activity	-10.98
LOC113690048	trans-resveratrol di-O-methyltransferase-like isoform X1	transferase activity; protein binding	-11.80

LOC113689971	trans-resveratrol di-O-methyltransferase-like	transferase activity; protein binding	-12.20

Table S6. Top up- and down-regulated DEGs in CL153 plants at 37 ºC vs. 25 ºC, under either WW or SWD conditions.

Gene ID	ORF Name	Protein Name	Main Functions	Log_2FC
		**WW**		
		**Up-DEGs**		
Cc00_g34160	GSCOC_T00005086001	18.5 kDa class I heat shock protein		10.21
Cc02_g23670	GSCOC_T00015069001	22.7 kDa class IV heat shock protein		8.96
Cc10_g09350	GSCOC_T00031557001	12-oxophytodienoate reductase 1	ion binding; oxidoreductase activity	8.76
Cc07_g14930	GSCOC_T00036551001	UDP-glycosyltransferase 85A1	glycosyltransferase activity	8.46
Cc11_g16360	GSCOC_T00038174001	Heat shock protein. putative		8.35
Cc08_g14790	GSCOC_T00035388001	Putative Transcription factor GLABRA 3	protein binding	8.23
Cc07_g04930	GSCOC_T00039754001	Putative TRICHOME BIREFRINGENCE-LIKE 19		7.79
Cc02_g07140	GSCOC_T00009477001	18.2 kDa class I heat shock protein		7.70
Cc04_g05250	GSCOC_T00022272001	Heat shock 70 kDa protein	ATP binding; hydrolase activity	7.42
Cc06_g23680	GSCOC_T00040538001	6-phosphofructokinase 3	kinase activity	7.35
		**Down-DEGs**		
Cc04_g14850	GSCOC_T00040767001	dCTP pyrophosphatase 1	ion binding; hydrolase activity	-7.15

Cc06_g16790	GSCOC_T00004130001	Vignain	hydrolase activity	-7.20
Cc00_g06630	GSCOC_T00007484001	Putative Bifunctional dihydroflavonol 4-reductase/flavanone 4-reductase	catalytic activity	-7.43
Cc06_g14270	GSCOC_T00041278001	Putative Late embryogenesis abundant (LEA) hydroxyproline-rich glycoprotein family		-7.60
Cc02_g02650	GSCOC_T00020126001	Short-chain dehydrogenase TIC 32. chloroplastic	oxidoreductase activity	-7.64
Cc01_g08250	GSCOC_T00015525001	Putative Receptor-like protein 12		-7.80
Cc00_g33290	GSCOC_T00010540001	Putative Bifunctional dihydroflavonol 4-reductase/flavanone 4-reductase		-7.97
Cc10_g14320	GSCOC_T00012023001	Putative uncharacterized protein	oxidoreductase activity	-8.16
Cc00_g29810	GSCOC_T00010430001	NAD(P)-binding Rossmann-fold superfamily protein		-9.28
Cc04_g14850	GSCOC_T00040767001	dCTP pyrophosphatase 1	ion binding; hydrolase activity	-7.15
		**SWD**		
		**Up-DEGs**		
Cc09_g07380	GSCOC_T00036269001	Putative Reticuline oxidase-like protein	oxidoreductase activity; FAD binding	10.42
Cc03_g15270	GSCOC_T00026800001	Putative Reticuline oxidase-like protein	oxidoreductase activity; FAD binding	9.58
Cc04_g08040	GSCOC_T00022652001	Tropinone reductase homolog At1g07440	oxidoreductase activity	9.52
Cc09_g07390	GSCOC_T00036268001	Putative Reticuline oxidase-like protein	oxidoreductase activity; FAD binding	9.23
Cc10_g15150	GSCOC_T00033683001	Peroxidase 12	heme binding; ion binding; oxidoreductase activity	8.94

Cc08_g13570	GSCOC_T00030328001	Putative Mavicyanin	oxidoreductase activity	8.64
Cc06_g09490	GSCOC_T00041906001	Probable sulfate transporter 3.5	transporter activity	8.49
Cc11_g06140	GSCOC_T00020608001	Putative Flavanone 7-O-glucoside 2''-O-beta-L-rhamnosyltransferase	glycosyltransferase activity	8.46
Cc03_g04540	GSCOC_T00026176001	3-ketoacyl-CoA synthase 17	acyltransferase activity	8.34
Cc08_g00160	GSCOC_T00005571001	Putative Transmembrane 9 superfamily member 4		8.17
		**Down-DEGs**		
Cc02_g31930	GSCOC_T00027470001	Putative UDP-glycosyltransferase 83A1	glycosyltransferase activity	-8.53
Cc01_g12750	GSCOC_T00028220001	17.6 kDa class I heat shock protein 3		-8.76
Cc00_g00440	GSCOC_T00036129001	Putative Tudor/PWWP/MBT superfamily protein		-8.78
Cc11_g03840	GSCOC_T00033992001	Putative late blight resistance protein homolog R1B-14	ADP binding	-8.84
Cc03_g08140	GSCOC_T00033206001	Putative Reticulon-like protein B13		-9.09
Cc06_g19020	GSCOC_T00028944001	Putative NADP-dependent alkenal double bond reductase P1	oxidoreductase activity	-9.10
Cc02_g26640	GSCOC_T00024964001	Putative 26S proteasome non-ATPase regulatory subunit 1		-9.42
Cc02_g10170	GSCOC_T00029558001	Synaptonemal complex protein 1		-9.57
Cc08_g06230	GSCOC_T00040964001	Putative Glutamate receptor 2.7	ion channel activity	-9.65
Cc06_g23160	GSCOC_T00040611001	Putative disease resistance protein At1g50180	ATP binding; protein binding	-10.53

Table S7. Top 10 up- and down-regulated annotated DEGs in CL153 plants at 42 ºC vs. 25 ºC, under WW or SWD conditions.

Gene ID	ORF Name	Protein Name	Main Functions	Log_2FC
		**WW**		
		**Up-DEGs**		
Cc00_g34160	GSCOC_T00005086001	18,5 kDa class I heat shock protein		11.64
Cc02_g07130	GSCOC_T00009475001	18,5 kDa class I heat shock protein		11.59
Cc02_g07140	GSCOC_T00009477001	18,2 kDa class I heat shock protein		11.06
Cc04_g05250	GSCOC_T00022272001	Heat shock 70 kDa protein	ATP binding; hydrolase activity	10.93
Cc01_g12750	GSCOC_T00028220001	17,6 kDa class I heat shock protein 3		10.32
Cc02_g23670	GSCOC_T00015069001	22,7 kDa class IV heat shock protein		10.26
Cc06_g01960	GSCOC_T00023436001	17,5 kDa class I heat shock protein		9.53
Cc07_g12510	GSCOC_T00036863001	ATP-dependent zinc metalloprotease FTSH 6, chloroplastic	ATP binding; hydrolase activity	9.17
Cc06_g23680	GSCOC_T00040538001	6-phosphofructokinase 3	kinase activity	8.93
Cc11_g16360	GSCOC_T00038174001	Heat shock protein, putative		8.77
		**Down-DEGs**		
Cc10_g10350	GSCOC_T00031425001	Wall-associated receptor kinase 5	ATP binding; kinase activity	-7.55
Cc03_g08650	GSCOC_T00033294001	Putative Probable LRR receptor-like serine/threonine-protein kinase At4g08850	ATP binding; kinase activity	-7.70

Cc11_g07510	GSCOC_T00018046001	Putative LRR receptor-like serine/threonine-protein kinase GSO1	ATP binding; kinase activity	-7.86
Cc04_g16340	GSCOC_T00035723001	Expansin-A15		-7.88
Cc08_g13450	GSCOC_T00030343001	Putative MATE efflux family protein 9	transporter activity	-8.17
Cc03_g14200	GSCOC_T00031814001	Plant basic secretory protein (BSP) family protein		-8.20
Cc07_g09120	GSCOC_T00040308001	Anthocyanidin 3-O-glucosyltransferase 5	glycosyltransferase activity	-8.79
Cc02_g12790	GSCOC_T00029236001	Putative S-linalool synthase	ion binding; lyase activity	-9.80
Cc01_g08330	GSCOC_T00015536001	Putative Receptor-like protein 12		-10.01
Cc03_g12410	GSCOC_T00015223001	Chitinase 2	hydrolase activity	-11.45
		**SWD**		
		**Up-DEGs**		
Cc02_g07140	GSCOC_T00009477001	18,2 kDa class I heat shock protein		11.37
Cc01_g12750	GSCOC_T00028220001	17,6 kDa class I heat shock protein 3		8.93
Cc04_g02380	GSCOC_T00021909001	GDSL esterase/lipase APG	hydrolase activity	8.57
Cc04_g06890	GSCOC_T00022490001	Probable non-specific lipid-transfer protein AKCS9		8.33
Cc06_g14690	GSCOC_T00041223001	Cellulose synthase A catalytic subunit 7 [UDP-forming]	ion binding; glycosyltransferase activity	8.31
Cc00_g22470	GSCOC_T00009593001	COBRA-like protein 4		8.29

Cc03_g06860	GSCOC_T00026510001	Auxin-responsive family protein	ion binding	8.28
Cc00_g18380	GSCOC_T00005502001	Momilactone A synthase	oxidoreductase activity	8.28
Cc05_g02500	GSCOC_T00020945001	GDSL esterase/lipase At2g04570	hydrolase activity	8.04
Cc01_g21320	GSCOC_T00004993001	Probable beta-D-xylosidase 2	hydrolase activity	7.98
		**Down-DEGs**		
Cc11_g06140	GSCOC_T00020608001	Putative Flavanone 7-O-glucoside 2''-O-beta-L-rhamnosyltransferase	glycosyltransferase activity	-7.77
Cc08_g06960	GSCOC_T00025284001	Putative Vetispiradiene synthase 1	ion binding; lyase activity	-7.94
Cc10_g15140	GSCOC_T00033682001	Peroxidase 12	heme binding; ion binding; oxidoreductase activity	-8.07
Cc00_g03750	GSCOC_T00013262001	Putative ABC transporter B family member 9	ATP binding; transporter activity	-8.10
Cc04_g10600	GSCOC_T00042618001	Putative Cytochrome P450 82A3	heme binding; ion binding; oxidoreductase activity	-8.13
Cc04_g10590	GSCOC_T00042615001	Putative Cytochrome P450 82A1 (Fragment)	heme binding; ion binding; oxidoreductase activity	-8.47
Cc10_g15150	GSCOC_T00033683001	Peroxidase 12	heme binding; ion binding; oxidoreductase activity	-8.92
Cc11_g07770	GSCOC_T00018007001	Pathogenesis-related protein R major form		-9.01
Cc06_g09490	GSCOC_T00041906001	Probable sulfate transporter 3.5	transporter activity	-9.23
Cc09_g07380	GSCOC_T00036269001	Putative Reticuline oxidase-like protein	FAD binding; oxidoreductase activity	-9.24

Table S8. Top 10 up- and down-regulated annotated DEGs in CL153 plants at REC14 vs. 25 ºC, under WW or SWD conditions.

Gene ID	ORF Name	Protein Name	Main Functions	Log_2FC
		**WW**		
		**Up-DEGs**		
Cc07_g04930	GSCOC_T00039754001	Putative TRICHOME BIREFRINGENCE-LIKE 19		7.61
Cc05_g08510	GSCOC_T00016875001	Putative RING/FYVE/PHD zinc finger superfamily protein	ion binding	7.33
Cc01_g09640	GSCOC_T00024027001	Family of unknown function (DUF662)		6.63
Cc06_g10840	GSCOC_T00041728001	Inositol oxygenase 2	oxidoreductase activity; ion binding	6.56
Cc02_g36620	GSCOC_T00003900001	LOB domain-containing protein 30		6.39
Cc02_g24560	GSCOC_T00028868001	Putative Metalloendoproteinase 1	hydrolase activity; ion binding	6.20
Cc08_g16900	GSCOC_T00035666001	Putative NAC domain-containing protein 12	DNA binding	5.89
Cc02_g39250	GSCOC_T00027744001	Long chain acyl-CoA synthetase 4	ligase activity	5.89
Cc00_g24100	GSCOC_T00001080001	GDSL esterase/lipase At1g28580	hydrolase activity	5.80
Cc06_g14690	GSCOC_T00041223001	Cellulose synthase A catalytic subunit 7 [UDP-forming]	glycosyltransferase activity; ion binding	5.64
		**Down-DEGs**		
Cc11_g11720	GSCOC_T00032409001	MADS-box protein CMB1	transcription regulator activity; DNA binding; protein binding	-5.95

Cc02_g23880	GSCOC_T00015092001	ABC transporter G family member 11	ATP binding; transporter activity	-6.09
Cc09_g05150	GSCOC_T00007088001	Putative Cytochrome P450 89A2	heme binding; ion binding; oxidoreductase activity	-6.14
Cc00_g06630	GSCOC_T00007484001	Putative Bifunctional dihydroflavonol 4-reductase/flavanone 4-reductase	catalytic activity	-6.37
Cc06_g16330	GSCOC_T00030708001	Cytochrome P450 93A1	heme binding; ion binding; oxidoreductase activity	-7.15
Cc00_g30530	GSCOC_T00007195001	Putative Cytochrome P450 83B1	heme binding; ion binding; oxidoreductase activity	-7.44
Cc03_g14200	GSCOC_T00031814001	Plant basic secretory protein (BSP) family protein		-8.10
Cc06_g22450	GSCOC_T00005214001	Putative Cytochrome P450 83B1	heme binding; ion binding; oxidoreductase activity	-9.04
Cc00_g33290	GSCOC_T00010540001	Putative Bifunctional dihydroflavonol 4-reductase/flavanone 4-reductase		-9.60
Cc00_g29810	GSCOC_T00010430001	NAD(P)-binding Rossmann-fold superfamily protein		-9.74
		**SWD**		
		**Up-DEGs**		
Cc06_g01620	GSCOC_T00023390001	Laccase-4	oxidoreductase activity; ion binding	10.20
Cc05_g09650	GSCOC_T00021556001	Chlorophyll a-b binding protein 1. chloroplastic	chlorophyll binding	9.94
Cc10_g09410	GSCOC_T00031548001	Putative Protein AIG1	GTP binding	9.13
Cc08_g04710	GSCOC_T00042505001	Putative TRAF-like family protein		9.07

Cc10_g01820	GSCOC_T00024670001	Endoglucanase 24	cellulase activity	8.85
Cc10_g09420	GSCOC_T00031547001	Putative Protein AIG1	GTP binding	8.84
Cc11_g08360	GSCOC_T00032886001	Putative MLP-like protein 28		8.77
Cc02_g34390	GSCOC_T00000680001	Putative pectinesterase/pectinesterase inhibitor 22	hydrolase activity; enzyme inhibitor activity	8.66
Cc01_g12960	GSCOC_T00028253001	Expansin-A4		8.59
Cc00_g13760	GSCOC_T00013299001	Pectinesterase/pectinesterase inhibitor PPE8B	hydrolase activity; enzyme inhibitor activity	8.53
		**Down-DEGs**		
Cc04_g07380	GSCOC_T00022554001	Putative Protein ASPARTIC PROTEASE IN GUARD CELL 1	hydrolase activity	-4.31
Cc01_g10130	GSCOC_T00024094001	Histone H2B.2	DNA binding; protein binding	-4.40
Cc06_g02060	GSCOC_T00023446001	Putative Encodes a protein involved in salt tolerance. names SIS (Salt Induced Serine rich).		-4.46
Cc05_g00080	GSCOC_T00017025001	Late embryogenesis abundant protein EMB564		-4.47
Cc01_g14440	GSCOC_T00028443001	Protein kinase domain-containing protein	ATP binding; kinase activity	-4.55
Cc06_g05680	GSCOC_T00043130001	Putative Protein ABSCISIC ACID-INSENSITIVE 5	transcription regulator activity; DNA binding; protein binding	-4.77
Cc08_g13570	GSCOC_T00030328001	Putative Mavicyanin	oxidoreductase activity	-5.19
Cc01_g20380	GSCOC_T00015940001	Glutaredoxin-C11	oxidoreductase activity	-5.21

Cc00_g05510	GSCOC_T00006857001	Putative ABC transporter B family member 9	ATP binding; transporter activity	-6.13
Cc02_g15140	GSCOC_T00013950001	transcription factor-related	DNA binding	-6.33

Table S9. Regulation patterns among DEGs in Icatu plants under WW or after gradual exposure to SWD, and after temperature increase to 37 ºC and 42 ºC. Red: up-regulated DEGs; Blue: down-regulated DEGs.

Gene ID	Protein Name	WW			SWD		
		37 ºC	42 ºC	REC14	37 ºC	42 ºC	REC14
LOC113742299	Aspartic protease in guard cell 1-like	3.99					-5.80
LOC113741996	LEA protein Dc3-like						3.80
LOC113713775	Endoplasmin homolog		-3.37			-1.84	-2.40
LOC113742697	Aspartic protease in guard cell 1-like	5.32	2.78				-4.94
LOC113719150	Acidic endochitinase-like	9.38					
LOC113708853	Aquaporin PIP2-7						-1.56
LOC113717922	Acidic endochitinase-like	5.13	-2.06			2.63	
LOC113742593	Aspartic protease in guard cell 1-like isoform X2	6.53					-2.79
LOC113704200	Homeobox-leucine zipper protein ATHB-12-like						5.24
LOC113734216	Auxin-responsive protein IAA14-like						-2.89
LOC113737208	Basic endochitinase-like		4.29				-6.79
LOC113727829	Galactinol synthase 2-like						5.52
LOC113716416	Acidic endochitinase-like	7.42					
LOC113696040	Aquaporin PIP2-2-like	4.70				3.47	-1.99
LOC113708828	Aquaporin PIP2-4-like						-3.03
LOC113695179	Transcription factor DIVARICATA-like						-2.38
LOC113738429	Transcription factor MYBS1-like						
LOC113732068	Protein eceriferum 1-like	6.82					-3.55
LOC113733849	Galactinol synthase 2-like isoform X3						1.97

LOC113699014	MYB-related protein 306-like	5.89					-4.16
LOC113740410	HVA22-like protein e						3.52
LOC113740563	HVA22-like protein e						4.49
LOC113691066	Peroxidase 3-like						5.03
LOC113740436	LEA protein Dc3-like	-3.50				1.93	3.10
LOC113742753	Aquaporin TIP2-1						-6.46
LOC113733440	WRKY DNA-binding transcription factor 70-like	3.93	-3.63				
LOC113716691	Aspartic protease in guard cell 1-like	2.60	2.99				-3.50
LOC113738017	Galactinol synthase 2-like	3.59					
LOC113695827	Pathogenesis-related protein PR-1-like						-4.78
LOC113742898	Transcription factor MYBS1-like	3.66					-1.80
LOC113735055	Basic endochitinase-like		2.44				-8.11
LOC113743471	Chlorophyll a-b binding protein 36	6.78					-2.56
LOC113689822	Chlorophyll a-b binding protein 36				-2.81		-2.39
LOC113710329	Aquaporin PIP2-4-like						-3.31
LOC113733000	Galactinol synthase 2-like						7.90
LOC113727571	Phenylalanine ammonia-lyase G4-like	3.19	3.60			2.60	-2.31
LOC113703008	Protein phosphatase 2C 51-like				4.66	2.40	4.49
LOC113743330	F-box/LRR-repeat MAX2 homolog A-like		-3.70				
LOC113702920	Abscisic acid receptor PYR1-like						-2.09
LOC113727830	Galactinol synthase 2-like		-2.43				7.23
LOC113706865	Chitinase-like protein 2						-2.29
LOC113711139	Zinc finger protein ZAT10-like						4.01
LOC113691849	Protein NLP1-like isoform X1	8.49					
LOC113732870	Stress-related protein-like	6.55					0.86
LOC113736371	Glycine-rich RNA-binding protein 4	5.99					1.65
LOC113731075	Non-specific lipid-transfer protein 1-like	12.18				8.82	

LOC113732919	Probable protein phosphatase 2C 34	5.91	2.03				-1.41
LOC113702113	Serine/threonine-protein kinase SAPK1-like isoform X3	4.33	3.16				-3.78
LOC113700985	AAA-atpase ASD, mitochondrial-like	10.60					-2.37
LOC113719011	Protein Aspartic protease in guard cell 1-like	7.05	3.28			3.95	
LOC113718757	Histidine kinase 4-like	6.38	2.30				-2.27
LOC113712121	Wall-associated receptor kinase 2-like	8.54					
LOC113731572	Squalene monooxygenase-like	5.63	4.36				
LOC113738233	Sugar transport protein 13-like	4.05					
LOC113689755	F-box/LRR-repeat MAX2 homolog A-like	-4.58	-3.28				2.42
LOC113726525	Zinc finger protein ZAT10-like	-4.05	-4.25				4.19
LOC113736251	Beta-amylase 1	4.37					
LOC113718216	Protein senescence-associated gene 21	3.87					
LOC113735267	Sucrose synthase 2-like	-2.48	-2.70				
LOC113707447	Pleiotropic drug resistance protein 1-like	3.88	2.76				-1.26
LOC113743599	NAC domain-containing protein 72-like	3.93	-1.80	2.47			4.10
LOC113694515	Calmodulin-binding protein 25-like	2.99			2.70		1.55
LOC113732513	Annexin D3-like isoform X4	7.29	2.31				
LOC113701789	Zeaxanthin epoxidase	2.73					-1.99
LOC113732515	Annexin D3-like	7.17					
LOC113729974	Acidic endochitinase SE2-like	4.06			3.40		
LOC113707625	Phenylalanine ammonia-lyase G4-like	4.60	3.02				-2.68
LOC113699229	UDP-glycosyltransferase 74E2-like	-3.63					
LOC113740136	Protein Aspartic protease in guard cell 1-like	3.21	2.72				-4.57
LOC113717283	Uncharacterized protein LOC113717283 isoform X2	2.52	1.27				
LOC113701327	Probable hexokinase-like 2 protein isoform X1	4.86	3.18				-2.85
LOC113720826	Homeobox-leucine zipper protein ATHB-7-like	-3.29	-1.91				
LOC113699192	RNA-binding protein 24-like isoform X6	-2.05					2.37

LOC113701593	PLAT domain-containing protein 3-like	6.33					
LOC113714561	Squalene monooxygenase-like	3.35	2.25				-2.31
LOC113701491	Protein cellulose synthase interactive 1-like	1.73	1.75				
LOC113717599	Protein early-responsive to dehydration 7	3.94					1.83
LOC113702816	Plastidal glycolate/glycerate translocator 1	1.73					
LOC113714504	Bzip transcription factor 46-like		-1.32				3.09
LOC113709223	Aquaporin TIP1-3-like		3.54				-6.20
LOC113732999	Galactinol synthase 2-like		-2.81				2.44
LOC113725645	Transcription factor DIVARICATA-like						3.74
LOC113699393	Homeobox-leucine zipper protein HAT22-like						3.11
LOC113706564	Protein phosphatase 2C 51-like					3.25	5.16
LOC113697821	Xyloglucan endotransglucosylase/hydrolase protein 6						-10.98
LOC113712167	Phosphoprotein ECPP44-like						1.85
LOC113695700	Protein LE25-like						3.87
LOC113713327	Annexin D1-like						2.36
LOC113726435	Abscisic acid-insensitive 5-like protein 7 isoform X2						1.41
LOC113693754	Non-specific lipid-transfer protein 1-like						2.10
LOC113707187	Aquaporin TIP4-1-like		4.06				-6.49
LOC113689826	NAC domain-containing protein 72-like		-1.64	3.08			4.05
LOC113708818	Glycerate dehydrogenase-like		1.71				-1.54
LOC113690342	Acidic endochitinase-like						-2.87
LOC113700062	Serine/threonine-protein kinase SAPK1-like isoform X3		3.27				-3.61
LOC113731163	WRKY transcription factor WRKY24-like		-2.01				2.14
LOC113688101	Protein thylakoid formation1						-1.51
LOC113692069	MYB-related protein 306-like		2.68			2.38	-1.77
LOC113697378	Temperature-induced lipocalin-1-like		-3.04				1.55
LOC113707439	LRR receptor-like serine/threonine-protein kinase		3.26				-3.04

	ERECTA isoform X1				
LOC113716433	Transcription factor MYB124	1.31			-1.78
LOC113733193	Aquaporin PIP1-2				-4.14
LOC113733488	LEA protein-like				4.23
LOC113739398	Ethylene-responsive transcription factor WIN1-like				-7.45
LOC113698209	Aspartic proteinase nepenthesin-1-like				-6.86
LOC113716753	Protein early-responsive to dehydration 7				1.30
LOC113706149	Probable LRR receptor-like serine/threonine-protein kinase At1g34110				-6.65
LOC113692189	LEA protein 46-like				4.25
LOC113692484	PHD finger protein ALFIN-LIKE 4-like				-2.17
LOC113691904	Myb-related protein 306-like	2.48		2.91	-1.49
LOC113703384	Chitinase-like protein 2				-2.96
LOC113742441	Protein Aspartic protease in guard cell 1-like				-8.61
LOC113728077	Probable aquaporin PIP1-2				-2.59
LOC113691442	CBL-interacting serine/threonine-protein kinase 6-like				0.96
LOC113732575	Protein zinc induced facilitator-like 1-like isoform X4				-1.43
LOC113701531	AAA-atpase ASD	-3.30			-3.30
LOC113707285	Plastidal glycolate/glycerate translocator 1				-0.96
LOC113702672	LRR receptor-like serine/threonine-protein kinase	2.69			-2.32
LOC113726161	Non-specific lipid-transfer protein 1-like				-3.50
LOC113730387	Probable aquaporin PIP1-2	5.63		4.70	
LOC113725206	Ethylene-responsive transcription factor ERF106	2.73		2.99	
LOC113732511	Annexin D4-like	10.06		5.22	
LOC113742144	Pleiotropic drug resistance protein 1-like	1.84			
LOC113718268	Sucrose synthase 2-like	-2.62			
LOC113729880	Homeobox-leucine zipper protein ATHB-12-like	-1.96			

LOC113716326	Alpha-1,4 glucan phosphorylase L-2 isozyme, chloroplastic/amyloplastic-like isoform X2	3.18
LOC113730173	Beta-fructofuranosidase, soluble isoenzyme I-like isoform X2	-2.34
LOC113707992	Aquaporin PIP2-2-like	-2.16

Table S10. Regulation pattern among DEGs related to drought in CL153 plants under WW or after gradual exposure to SWD, and after temperature increase to 37 ºC and 42 ºC. Red: up-regulated DEGs; Blue: down-regulated DEGs.

Gene ID	Protein Name	WW			SWD		
		37 ºC	42 ºC	REC14	37 ºC	42 ºC	REC14
Cc11_g06260	Histidine kinase 4		-2.34				
Cc01_g11790	Mitogen-activated protein kinase 3		-1.90		1.72	-2.27	
Cc06_g09540	Multiprotein-bridging factor 1c	3.64			-3.30	5.02	
Cc02_g15480	Cellulose synthase A catalytic subunit 8 [UDP-forming]	5.12		5.60		7.11	
Cc01_g13190	Laccase-2	5.17					
Cc04_g09640	Protein aspartic protease in guard cell 1	2.08					4.74
Cc07_g15660	Binding	1.17					1.71
Cc04_g07380	Putative Protein aspartic protease in guard cell 1					-3.65	-4.31
Cc04_g07360	Putative Protein aspartic protease in guard cell 1					-3.03	-3.72
Cc06_g15980	18 kDa seed maturation protein					-5.48	
Cc04_g09630	Putative Protein aspartic protease in guard cell 1				4.74		
Cc08_g00950	Putative overexpressor of cationic peroxidase 3				-2.89		
Cc07_g07560	Xyloglucan endotransglucosylase/hydrolase protein 6						5.30
Cc04_g07350	Putative Protein aspartic protease in guard cell 1						4.89
Cc07_g10030	Dehydrin DH1a						-3.59
Cc04_g08280	Putative movement protein binding protein 2C						2.79
Cc04_g07370	Putative Protein aspartic protease in guard cell 1						3.24
Cc01_g08980	LEA hydroxyproline-rich glycoprotein family						3.01
Cc06_g05680	Putative Protein abscisic acid-insensitive 5						-4.77

Table S11. Regulation pattern among DEGs related to heat stress, in Icatu plants under WW or after gradual exposure to SWD, and after temperature increase to 37 ºC and 42 ºC. Red: up-regulated DEGs; Blue: down-regulated DEGs.

Gene ID	Protein Name	WW			SWD		
		37 ºC	42 ºC	REC14	37 ºC	42 ºC	REC14
LOC113741996	LEA protein Dc3-like						3.80
LOC113713775	Low quality protein: endoplasmin homolog			-3.37	-1.84		-2.40
LOC113729908	Luminal-binding protein 5-like			-3.46			-2.56
LOC113703837	14 kda proline-rich protein DC2.15-like	8.61		5.16	1.98		-4.36
LOC113719150	Acidic endochitinase-like	9.38					
LOC113717922	Acidic endochitinase-like	5.13		-2.06	2.63		
LOC113716652	Uncharacterized protein LOC113716652			3.42	2.21		3.14
LOC113718867	Uncharacterized protein LOC113718867	3.66		2.52	2.07		2.71
LOC113700194	Carbonic anhydrase 2-like			2.36			-3.36
LOC113698717	3-ketoacyl-coa synthase 6-like			3.73	3.83		-3.85
LOC113737208	Basic endochitinase-like			4.29			-6.79
LOC113694491	Auxin-binding protein ABP20-like	12.41					-5.50
LOC113727829	Galactinol synthase 2-like						5.52
LOC113699255	MADS-box protein SVP-like						-2.92
LOC113716416	Acidic endochitinase-like	7.42					
LOC113701649	3-ketoacyl-coa synthase 6	6.29		2.93			-5.22
LOC113691612	Auxin-binding protein ABP20-like	4.13					-7.42
LOC113733849	Galactinol synthase 2-like isoform X3						1.97
LOC113740410	HVA22-like protein e						3.52
LOC113740563	HVA22-like protein e						4.49
LOC113691066	Peroxidase 3-like						5.03

LOC113740436	LEA protein Dc3-like	-3.50			1.93		3.10
LOC113734200	3-ketoacyl-coa synthase 11-like			4.44			-5.28
LOC113713310	E3 ubiquitin-protein ligase CHIP-like			-2.50	-2.39		-1.99
LOC113740295	Chlorophyll a-b binding protein P4						-2.88
LOC113738017	Galactinol synthase 2-like	3.59					
LOC113713768	Small ubiquitin-related modifier 1-like						2.10
LOC113704068	Oleosin 1-like isoform X1	4.40					3.13
LOC113731964	22.0 kda class IV heat shock protein-like	-14.90	-9.19	-15.50	-8.72		
LOC113735055	Basic endochitinase-like			2.44			-8.11
LOC113711424	BAG family molecular chaperone regulator 4-like	3.58					-3.05
LOC113692232	Heat stress transcription factor C-1						2.34
LOC113730421	18.2 kda class I HSP-like	-8.06	-6.00	-10.14	-6.88		
LOC113743471	Chlorophyll a-b binding protein 36	6.78					-2.56
LOC113717780	18.5 kda class I heat shock protein-like	-6.58	-5.48	-11.11	-6.06		2.90
LOC113689822	Chlorophyll a-b binding protein 36		-2.81				-2.39
LOC113703784	BAG family molecular chaperone regulator 1-like	3.31					-4.39
LOC113707858	Calreticulin-3-like			-2.33			-2.47
LOC113733000	Galactinol synthase 2-like						7.90
LOC113706935	BAG family molecular chaperone regulator 3-like						-5.10
LOC113725542	3-ketoacyl-coa synthase 19-like	4.91		4.55			-7.48
LOC113727830	Galactinol synthase 2-like			-2.43			7.23
LOC113706865	Chitinase-like protein 2						-2.29
LOC113717777	Glutathione S-transferase-like						-1.58
LOC113711139	Zinc finger protein ZAT10-like						4.01
LOC113716752	Dnaj homolog subfamily B member 1-like	2.64		1.68			

LOC113718871	Small HSP	-10.42	-7.68	-11.25	-7.65		
LOC113729577	17.5 kDa class I HSP-like	-8.17	-6.37	-9.31	-6.51		
LOC113710359	17.3 kDa class I HSP -like	-5.24	-5.15	-10.54	-6.88		1.25
LOC113692361	Class I HSP-like	-7.78	-5.24	-8.75	-5.76		
LOC113736371	Glycine-rich RNA-binding protein 4	5.99					1.65
LOC113739647	Heat shock 70 kda protein-like	-10.37	-2.90	-11.85	-4.18		5.05
LOC113730422	17.4 kda class I HSP-like	-4.59	-2.82	-6.63	-4.11		1.77
LOC113695644	Class I HSP-like	-8.84	-4.61	-10.60	-5.30		3.33
LOC113727176	Ribulose bisphosphate carboxylase/oxygenase activase	-4.80		-5.68			4.30
LOC113701851	Serine hydroxymethyltransferase	3.72					
LOC113728149	BAG family molecular chaperone regulator 6-like	-7.42	-4.66	-9.24	-6.91		
LOC113718711	3-ketoacyl-coa synthase 6-like	7.12		4.59			-6.58
LOC113728228	L-ascorbate peroxidase 2	-2.76					
LOC113700985	AAA-atpase ASD	10.60					-2.37
LOC113724829	Small HSP	-6.63		-5.80	-2.26		2.27
LOC113724957	HSP 83-like	-6.65	-4.50	-7.62	-5.10		1.30
LOC113706907	3-ketoacyl-coa synthase 10	2.94		2.32			
LOC113725865	Protein heat-stress-associated 32-like	-4.90	-2.67	-5.13	-2.66		
LOC113703695	Acyl-lipid (9-3)-desaturase-like	5.68		3.50			-2.12
LOC113743529	Uncharacterized protein LOC113743529	5.02					
LOC113704386	HSP 83-like	-4.10		-3.80			1.22
LOC113729868	Small HSP	-6.01		-5.87	-2.67		2.35
LOC113742137	Chlorophyll a-b binding protein 4	6.80					-2.73
LOC113736588	Early light-induced protein 2	4.32					

LOC113731692	Chlorophyll a-b binding protein CP24 10A	6.52					-2.57
LOC113703907	Protein NDL2-like	5.26		5.53			-2.73
LOC113732386	Ribulose bisphosphate carboxylase/oxygenase activase	-3.52		-4.39			2.46
LOC113697052	Histone H2A.V-like isoform X1	3.88					1.92
LOC113743195	Grpe protein homolog 1	8.21		-2.41	-1.41		
LOC113734176	Early light-induced protein 2	-2.64		-2.19			
LOC113730852	Protein heat-stress-associated 32-like	-3.25		-5.28	-2.51		
LOC113740171	Chaperone protein clpb1-like	-4.62	-3.16	-6.62	-4.08		
LOC113710048	BAG family molecular chaperone regulator 3-like	2.01		2.35			
LOC113726525	Zinc finger protein ZAT10-like	-4.05		-4.25			4.19
LOC113701947	MADS-box protein SVP-like isoform X2	7.77					
LOC113733368	3-ketoacyl-coa synthase 1-like	4.53		4.32			-1.84
LOC113718216	Protein senescence-associated gene 21	3.87					
LOC113707447	Pleiotropic drug resistance protein 1-like	3.88		2.76			-1.26
LOC113730226	Serine/threonine-protein kinase atpk2/atpk19-like	4.01					
LOC113695980	VIN3-like protein 2	6.54		3.18			-3.13
LOC113704294	3-ketoacyl-coa synthase 11-like	2.89		1.95			
LOC113715331	Uncharacterized protein LOC113715331	-3.76		-3.44	-2.63		
LOC113703627	Organic cation/carnitine transporter 3-like	7.99		4.04			
LOC113706843	Acyl-lipid (9-3)-desaturase-like	3.15		4.34			-2.46
LOC113716426	3-ketoacyl-coa synthase 6-like	4.04		3.32			-5.06
LOC113739654	17.4 kda class III heat shock protein-like	-2.85		-4.66	-3.10		1.20
LOC113732513	Annexin D3-like isoform X4	7.29		2.31			
LOC113725195	Derlin-1.2-like	2.70		1.51			
LOC113701789	Zeaxanthin epoxidase	2.73					-1.99

LOC113732515	Annexin D3-like	7.17					
LOC113690412	60S acidic ribosomal protein P0-like	-5.19					
LOC113695236	Xyloglucan endotransglucosylase/hydrolase protein 22-like	4.36					4.37
LOC113729974	Acidic endochitinase SE2-like	4.06	3.40				
LOC113742776	Ribulose-phosphate 3-epimerase	1.86					-1.04
LOC113736734	Calreticulin-3-like	2.65					-1.50
LOC113742703	Transcription factor ICE1-like	4.79					
LOC113701327	Probable hexokinase-like 2 protein isoform X1	4.86		3.18			-2.85
LOC113730010	HSP 83	-3.20		-8.32	-5.49		1.29
LOC113705049	Dnaj protein homolog ANJ1-like	-2.33		-4.42	-3.22		
LOC113733363	CBL-interacting serine/threonine-protein kinase 9-like isoform X3	3.14		1.81			-1.51
LOC113701593	PLAT domain-containing protein 3-like	8.33					
LOC113717599	Protein early-responsive to dehydration 7	3.94					1.83
LOC113691978	Xyloglucan endotransglucosylase/hydrolase protein 22-like					2.55	4.41
LOC113732999	Galactinol synthase 2-like			-2.81			2.44
LOC113701240	Ribulose bisphosphate carboxylase small chain SSU11A, chloroplastic-like isoform X2			2.40			-1.83
LOC113693755	Fructose-1,6-bisphosphatase						-2.21
LOC113712167	Phosphoprotein ECPP44-like						1.85
LOC113697169	Fructose-1,6-bisphosphatase		-2.90				-2.62
LOC113695700	Protein LE25-like						3.87
LOC113713327	Annexin D1-like						2.36
LOC113705247	Oleosin 1-like						3.22

LOC113726667	Chlorophyll a-b binding protein CP24 10A	-3.37				-2.69
LOC113698165	Ribulose bisphosphate carboxylase small chain SSU8		2.05			-1.51
LOC113703720	Phosphoribulokinase					-1.41
LOC113690342	Acidic endochitinase-like					-2.87
LOC113706872	Phosphoribulokinase					-1.35
LOC113712439	Glyceraldehyde-3-phosphate dehydrogenase B					-1.62
LOC113731163	WRKY transcription factor WRKY24-like		-2.01			2.14
LOC113692069	MYB-related protein 306-like		2.68	2.38		-1.77
LOC113706908	Cellulose synthase-like protein D3		3.02			-5.02
LOC113742866	ACT domain-containing protein ACR11-like					-1.55
LOC113697378	Temperature-induced lipocalin-1-like		-3.04			1.55
LOC113703892	Cellulose synthase-like protein D3		2.49			-4.87
LOC113743506	Small ubiquitin-related modifier 1-like		-1.24			1.51
LOC113734640	Ras-related protein RABA5c-like					1.40
LOC113707439	LRR receptor-like serine/threonine-protein kinase		3.26			-3.04
LOC113716585	Chlorophyll a-b binding protein	-2.81				-1.40
LOC113733488	LEA protein-like					4.23
LOC113716753	Protein early-responsive to dehydration 7					1.30
LOC113692189	LEA protein 46-like					4.25
LOC113691904	MYB-related protein 306-like		2.48	2.91		-1.49
LOC113732295	Cinnamoyl-coa reductase 1-like					3.63
LOC113728213	CBL-interacting serine/threonine-protein kinase 9					-2.05
LOC113695405	Uncharacterized protein LOC113695405	8.28				4.54
LOC113715927	Heat stress transcription factor A-6b-like isoform X1		-3.65			2.59
LOC113703384	Chitinase-like protein 2					-2.96
LOC113731213	B-box zinc finger protein 18-like					1.29

LOC113726009	Dnaj protein homolog ANJ1	-5.00	-3.57		0.90
LOC113692188	36.4 kda proline-rich protein-like				-9.06
LOC113727543	Uncharacterized protein LOC113727543	-2.19	-2.62		-1.86
LOC113692744	VIN3-like protein 2				-2.73
LOC113726036	30S ribosomal protein S5				-1.31
LOC113701531	AAA-atpase ASD	-3.30			-3.30
LOC113692147	Tubulin beta-5 chain				-1.79
LOC113702672	LRR receptor-like serine/threonine-protein kinase	2.69			-2.32
LOC113741763	17.4 kda class III heat shock protein-like	-5.27	-3.93		
LOC113728215	3-ketoacyl-coa synthase 1	5.24	4.03		
LOC113718927	Uncharacterized protein LOC113718927	-4.14	-2.52		
LOC113732511	Annexin D4-like	10.06	5.22		
LOC113742616	Chaperone protein clpb1	-6.51			
LOC113742144	Pleiotropic drug resistance protein 1-like	1.84			
LOC113710407	BAG family molecular chaperone regulator 2-like	3.28			
LOC113730505	Heat shock cognate 70 kda protein 2-like	-2.46			
LOC113707789	BAG family molecular chaperone regulator 3-like	3.71			
LOC113716326	Alpha-1,4 glucan phosphorylase L-2 isozyme	3.18			
LOC113716310	Chaperonin 60 subunit beta 4	2.41			
LOC113713366	2-methylene-furan-3-one reductase-like	2.68			

Table S12. Regulation pattern among DEGs related to heat stress in CL153 plants under WW or after gradual exposure to SWD, and after temperature increase to 37 ºC and 42 ºC. Red: up-regulated DEGs; Blue: down-regulated DEGs.

Gene ID	Protein Name	WW			SWD		
		37 ºC	42ºC	REC14	37 ºC	42 ºC	REC14
Cc01_g07750	DnaJ protein homolog ANJ1	2.77	4.55		-2.36		
Cc02_g11810	Aldolase-type TIM barrel family protein	2.93	4.38				
Cc07_g13330	15.7 kDa heat shock protein	3.11	4.45		-4.99	5.05	
Cc07_g04080	Chaperone protein ClpB3	2.49	3.88		-2.66	2.74	
Cc01_g10720	RuBisCO large subunit-binding protein subunit alpha	2.05	2.99		-2.16		
Cc06_g15710	Putative Activator of 90 kDa heat shock protein ATPase homolog	2.14	4.93		-1.83	3.53	
Cc06_g09540	Multiprotein-bridging factor 1c	3.64	5.72		-3.30	5.02	
Cc03_g07040	Chaperonin CPN60-2	2.08	3.28				
Cc10_g15410	Chaperone protein dnaJ 1		2.48		-1.25		
Cc01_g00200	RuBisCO large subunit-binding protein subunit beta		2.25		-2.73		
Cc01_g11790	Mitogen-activated protein kinase 3		-1.90		1.72	-2.27	
Cc06_g23520	chaperone binding;ATPase activators		1.66		-2.23	2.76	
Cc02_g27440	Putative Chaperone protein dnaJ 1		1.62				
Cc10_g00420	Putative inositol requiring 1-1		2.54				
Cc03_g03680	Heat shock 70 kDa protein 8	3.55			-3.95	3.66	
Cc06_g12530	20 kDa chaperonin	1.80			-2.47	2.76	
Cc08_g10430	Pollen-specific protein SF21	2.60		2.80			
Cc02_g06330	Plant protein of unknown function (DUF828)	3.79					
Cc03_g04600	CBL-interacting serine/threonine-protein kinase 7	-1.13					
Cc01_g05820	Putative Protein grpE				-2.01	1.77	
Cc06_g15980	18 kDa seed maturation protein					-5.48	
Cc02_g07500	Ribulose bisphosphate carboxylase/oxygenase activase 1				-2.81		
Cc02_g16860	Arginine decarboxylase				1.72		
Cc10_g00150	Hypothetical protein				-2.14		
Cc08_g07790	3-ketoacyl-CoA synthase 10						2.44
Cc10_g06080	ATP synthase delta chain, chloroplastic						2.51

Cc06_g05680	Putative Protein abscisic acid-insensitive 5	-4.77
Cc04_g05520	Unknown protein DS12 from 2D-PAGE of leaf	2.48
Cc11_g15490	Alpha-glucan water dikinase	1.75

Table S13. Regulation pattern of transcription factors among DEGs in plants of Icatu at 37 ºC, 42 ºC or REC14 temperatures, relative to the control 25 ºC, and submitted to either WW or SWD.

**Gene ID**	**Protein Name**	**WW**			**SWD**		
		**37ºC**	**42ºC**	**REC14**	**37ºC**	**42ºC**	**REC14**
LOC113716818	AP2/ERF and B3 domain-containing transcription factor RAV1-like	5.90					
LOC113695145	uncharacterized protein LOC113695145 isoform X1		4.92			2.36	3.71
LOC113716803	AP2/ERF and B3 domain-containing transcription factor RAV1-like		-1.47				
LOC113704200	homeobox-leucine zipper protein ATHB-12-like						5.24
LOC113734216	auxin-responsive protein IAA14-like						-2.89
LOC113713445	protein RADIALIS-like 3						-2.02
LOC113699255	MADS-box protein SVP-like						-2.92
LOC113711904	protein RADIALIS-like 3						-1.95
LOC113694109	zinc finger CCCH domain-containing protein 20-like						3.24
LOC113695179	transcription factor DIVARICATA-like						-2.38
LOC113692309	uncharacterized protein LOC113692309		-1.61				
LOC113725809	auxin response factor 18						-2.26
LOC113699014	myb-related protein 306-like	5.89					-4.16

LOC113697518	transcription repressor MYB5-like isoform X2	5.54		
LOC113741789	ethylene-responsive transcription factor TINY-like	8.05		
LOC113716791	transcription factor BHLH089-like isoform X2			-3.58
LOC113737953	transcription factor MYB48-like			6.11
LOC113700657	NAC domain-containing protein 104-like			3.43
LOC113733440	WRKY DNA-binding transcription factor 70-like	3.93	-3.63	
LOC113735417	transcription factor MYB48-like			6.30
LOC113713768	small ubiquitin-related modifier 1-like			2.10
LOC113693711	auxin response factor 19-like isoform X2			-2.18
LOC113742898	transcription factor MYBS1-like	3.66		-1.80
LOC113717219	myb family transcription factor PHL8-like isoform X3			-3.12
LOC113692232	heat stress transcription factor C-1			2.34
LOC113695479	zinc finger protein 8-like		1.88	-1.66
LOC113732985	transcription factor bHLH149-like			1.85
LOC113706192	probable transcription factor MYB58			
LOC113717457	zinc finger protein CONSTANS-LIKE 13-like	3.35		1.36
LOC113731745	transcription factor UNE10-like	3.58	2.40	-3.22
LOC113740599	transcription factor bHLH137 isoform X2			-3.73
LOC113691476	ethylene-responsive transcription factor ERF011-like			4.76
LOC113711139	Low quality protein: zinc finger protein ZAT10-like			4.01

LOC113691849	protein NLP1-like isoform X1	8.49					
LOC113697153	transcription factor bHLH66-like		2.75				
LOC113709332	homeobox-leucine zipper protein ATHB-14-like isoform X2						-2.27
LOC113703593	zinc finger protein CONSTANS-LIKE 1-like isoform X1						-6.86
LOC113694146	protein NLP7-like isoform X1	7.79	5.08				-2.85
LOC113718795	transcription factor MYB3-like	7.95	1.80				
LOC113743486	ethylene-responsive transcription factor 4-like	8.23	-1.46				2.35
LOC113731730	ethylene-responsive transcription factor ERF038-like	8.88	5.36				
LOC113702309	NAC domain-containing protein 22-like	10.36					-2.40
LOC113695626	transcription factor bHLH48-like	5.24	2.26				-2.49
LOC113701251	protein indeterminate-domain 16-like isoform X1	8.52	3.54				-2.11
LOC113700432	WUSCHEL-related homeobox 8-like isoform X2	8.39					-3.00
LOC113702506	GATA transcription factor 7-like	3.63	3.33				-1.99
LOC113733288	probable WRKY transcription factor 53	7.92					
LOC113706592	myb-related protein Zm38-like	9.26	9.73				
LOC113698942	basic leucine zipper 25-like	-4.75					5.52
LOC113732874	transcription factor bHLH25-like	5.05	1.78				-3.04
LOC113734633	transcription factor bHLH77-like	7.74	2.70				-6.50
LOC113726525	zinc finger protein ZAT10-like	-4.05	-4.25				4.19
LOC113701947	MADS-box protein SVP-like isoform X2	7.77					

LOC113707336	probable WRKY transcription factor 50	4.32			
LOC113687744	squamosa promoter-binding-like protein 3	6.31			-3.25
LOC113743599	NAC domain-containing protein 72-like	3.93	-1.80	2.47	4.10
LOC113718870	transcription repressor MYB6-like	3.58			
LOC113696993	auxin response factor 19-like isoform X2	2.69			-2.57
LOC113712218	Low quality protein: high mobility group B protein 9	4.29	4.05		-2.96
LOC113735669	bZIP transcription factor 53-like	7.34			
LOC113726199	transcription factor MYB8-like	5.85	8.02		
LOC113703953	trichome differentiation protein GL1-like	2.80	3.69		
LOC113692474	transcription factor bHLH48-like	2.34	2.97		-2.35
LOC113702960	ethylene-responsive transcription factor WIN1-like	7.01			-4.80
LOC113712753	transcription factor TCP23-like	2.55			
LOC113708251	auxin response factor 4-like isoform X3	3.87	2.05		-3.92
LOC113716523	probable WRKY transcription factor 75	-4.38	-3.34		
LOC113705443	ethylene-responsive transcription factor WIN1-like	5.63	6.05		-7.45
LOC113742703	transcription factor ICE1-like	4.79			
LOC113714105	protein sulfur deficiency-induced 1	2.88	2.49		
LOC113731518	zinc finger protein ZAT10-like	-3.84			4.21
LOC113720826	homeobox-leucine zipper protein ATHB-7-like	-3.29	-1.91		
LOC113687905	NAC domain-containing protein 21/22-like isoform X1	6.42			

LOC113733322	ethylene-responsive transcription factor ERF023-like	4.58	7.78			
LOC113737039	protein PHR1-LIKE 3-like isoform X2	3.01				-2.57
LOC113714504	bZIP transcription factor 46-like		-1.32			3.09
LOC113728161	transcriptional activator TAF-1-like isoform X4					2.10
LOC113725645	transcription factor DIVARICATA-like					3.74
LOC113699393	homeobox-leucine zipper protein HAT22-like					3.11
LOC113701443	basic leucine zipper 25-like		-3.48			6.13
LOC113726050	ethylene-responsive transcription factor 4-like					3.05
LOC113738212	nuclear transcription factor Y subunit C-9-like isoform X2					2.49
LOC113698743	ethylene-responsive transcription factor 1B-like				2.16	4.39
LOC113703305	NAC domain-containing protein 71-like		5.31			-6.77
LOC113691985	nuclear transcription factor Y subunit A-4-like isoform X5		3.77			4.24
LOC113689800	ethylene-responsive transcription factor 4-like		-1.70			2.37
LOC113742449	auxin response factor 4-like					-4.76
LOC113701486	transcription factor bHLH96-like					-8.31
LOC113726435	Abscisic acid-insensitive 5-like protein 7 isoform X2					1.41
LOC113706326	transcription factor bHLH71-like isoform X3		4.13			-8.48
LOC113724891	zinc finger protein CO3 isoform X2		1.73			-2.96
LOC113689826	NAC domain-containing protein 72-like		-1.64	3.08		4.05
LOC113731163	WRKY transcription factor WRKY24-like		-2.01			2.14

LOC113692069	myb-related protein 306-like	2.68	2.38	-1.77
LOC113698999	cyclic dof factor 2-like isoform X2			2.22
LOC113734084	nuclear transcription factor Y subunit C-9-like isoform X2			1.76
LOC113698417	auxin-responsive protein IAA9-like			-4.57
LOC113734588	transcription factor bHLH77-like	2.53		-7.60
LOC113690090	squamosa promoter-binding-like protein 3	2.12		-2.54
LOC113743506	small ubiquitin-related modifier 1-like	-1.24		1.51
LOC113703212	transcription factor bHLH71-like isoform X3	5.99		-7.51
LOC113732873	transcription factor bHLH25-like			-6.04
LOC113698462	transcription factor bHLH96-like			-5.82
LOC113703642	scarecrow-like protein 27			-2.06
LOC113716433	transcription factor MYB124	1.31		-1.78
LOC113691573	transcription factor bHLH35-like			-2.31
LOC113711052	transcription factor bHLH74-like isoform X2			-3.89
LOC113698772	transcription factor HBI1 isoform X2			-2.41
LOC113739398	ethylene-responsive transcription factor WIN1-like			-7.45
LOC113696385	nuclear transcription factor Y subunit B-5-like			4.25
LOC113742607	trihelix transcription factor ASIL2-like			1.40
LOC113715533	ethylene-responsive transcription factor 12-like			3.11
LOC113726843	ethylene-responsive transcription factor ERF038-like	6.49		-3.55

LOC113691904	myb-related protein 306-like	2.48		2.91	-1.49
LOC113738715	auxin-induced protein 22A-like				-3.40
LOC113715927	heat stress transcription factor A-6b-like isoform X1	-3.65			2.59
LOC113741490	zinc finger protein CONSTANS-LIKE 16-like				-2.67
LOC113727812	transcription factor bHLH149-like				1.66
LOC113698467	scarecrow-like protein 32				-5.60
LOC113709337	probable WRKY transcription factor 31		6.24		3.88
LOC113705444	heat stress transcription factor B-2b-like	-2.47			1.43
LOC113726042	squamosa promoter-binding protein 1				-2.52
LOC113714662	zinc finger protein GAI-ASSOCIATED FACTOR 1-like		2.18	1.66	1.33
LOC113731213	B-box zinc finger protein 18-like				1.29
LOC113741511	nuclear transcription factor Y subunit A-6				1.52
LOC113726198	transcription factor MYB80-like isoform X2	11.06			-5.02
LOC113725206	Low quality protein: ethylene-responsive transcription factor ERF106	2.73		2.99	
LOC113700172	GATA transcription factor 5-like	2.46		1.93	
LOC113691565	ethylene-responsive transcription factor TINY-like	3.61		3.89	
LOC113718755	probable WRKY transcription factor 75	-3.72	4.69		
LOC113703561	probable WRKY transcription factor 40	-4.60			
LOC113725264	WRKY DNA-binding transcription factor 70-like	-4.09			
LOC113706489	transcription factor MYB4-like	6.15			

LOC113716505	NAC domain-containing protein 2-like	-2.49
LOC113706677	probable WRKY transcription factor 40	-5.11
LOC113704079	ethylene-responsive transcription factor RAP2-12-like	-3.25
LOC113729845	protein NTM1-like 9 isoform X2	-1.89
LOC113734626	transcription factor bHLH128-like isoform X2	2.30
LOC113725808	GATA transcription factor 8-like	1.56
LOC113693566	transcription factor bHLH66-like	2.26
LOC113729880	homeobox-leucine zipper protein ATHB-12-like	-1.96
LOC113695070	ethylene-responsive transcription factor TINY-like	4.00
LOC113713046	transcription factor IBH1-like	1.35
LOC113741845	transcription factor MYB4-like isoform X1	9.96
LOC113709456	WRKY transcription factor 28-like	3.34
LOC113725625	basic leucine zipper 34-like	3.74

Table S14. Regulation pattern of transcription factors among DEGs in CL153 plants at 37 ºC, 42 ºC or REC14 temperatures, relative to the control 25ºC, and submitted to either WW or SWD.

Gene ID	Protein Name	WW			SWD		
		37ºC	42ºC	REC14	37ºC	42ºC	REC14
Cc08_g14180	Heat stress transcription factor B-2b	2.19	3.79				
Cc01_g19020	Heat stress transcription factor A-6b	3.41	5.92				
Cc00_g28360	Putative Heat stress transcription factor B-2a	1.89	3.04		-2.53	2.59	
Cc06_g17660	Heat stress transcription factor B-3	-2.23	-3.84		3.83	-3.78	-1.66
Cc07_g09170	G-box-binding factor 1		2.32		-2.13		
Cc11_g12210	Putative Probable WRKY transcription factor 57	-2.16	-2.90				
Cc07_g16400	Probable WRKY transcription factor 51		-6.61				
Cc02_g28510	Putative RNA polymerase sigma factor rpoD	-2.89	-3.66				
Cc11_g11720	MADS-box protein CMB1	-3.54	-6.94	-5.95			
Cc00_g21560	Putative Probable WRKY transcription factor 72	-3.84	-4.06				
Cc02_g38910	Common plant regulatory factor 1	1.19	1.79				-1.40
Cc07_g13110	Putative Ethylene-responsive transcription factor 15		-6.28		2.62	-3.58	-4.14
Cc02_g05970	Putative Dehydration-responsive element-binding protein 2C		1.83				-1.91
Cc02_g03570	MADS-box protein SOC1		-1.58				
Cc08_g10810	Heat shock factor protein HSF30	5.42			-2.62	3.24	
Cc06_g06710	Putative Nuclear transcription factor Y subunit A-2	-2.76		-1.66			

Cc04_g05860	Hypothetical protein	-4.50				3.19
Cc08_g11060	Putative Probable WRKY transcription factor 50	-2.41	-2.08			
Cc00_g02800	Agamous-like MADS-box protein AGL8 homolog	-3.45				
Cc06_g10260	Dehydration-responsive element-binding protein 3	2.93				
Cc01_g21420	Putative Ocs element-binding factor 1	-1.31				
Cc11_g13150	Putative BHLH (Fragment)	1.77		-1.74		
Cc04_g04250	Ethylene-responsive transcription factor WIN1	2.05			7.58	
Cc10_g06050	Transcription factor TGA7	-1.96				
Cc02_g24810	Putative uncharacterized protein					4.98
Cc02_g39490	AP2/ERF domain-containing transcription factor		2.56			3.40
Cc07_g14150	MADS-box protein SVP				4.12	4.11
Cc02_g05270	Putative WRKY1				3.78	
Cc06_g12520	Ethylene-responsive transcription factor ERF017			4.22	-5.80	-3.23
Cc11_g07710	Two-component response regulator ARR2				-5.10	
Cc02_g03430	Dehydration-responsive element-binding protein 1D				-5.07	
Cc03_g05410	Ethylene-responsive transcription factor 1B			4.80		-3.04
Cc09_g01430	WRKY transcription factor 6			3.03		
Cc05_g11430	Transcription factor TCP4			-2.19		
Cc01_g18730	Probable WRKY transcription factor 21			-2.44		1.74
Cc05_g07590	Ethylene-responsive transcription factor 4			2.47		-1.93

Cc10_g10280	Putative Uncharacterized protein At4g06598		4.18	
Cc02_g00040	Putative RNA polymerase sigma-B factor		-1.96	
Cc02_g13700	Putative Ethylene-responsive transcription factor 4		2.56	-1.83
Cc00_g13890	Double WRKY type transfactor		2.22	
Cc02_g13600	Squamosa promoter-binding protein 1		-1.82	3.06
Cc10_g15850	MADS-box protein SVP		-1.79	
Cc02_g37070	Putative Transcription factor TCP19		-4.28	3.34
Cc02_g13680	Putative Ethylene-responsive transcription factor 12		2.76	
Cc08_g06420	Probable WRKY transcription factor 11		1.42	
Cc06_g17760	Transcription initiation factor IIB-2		1.20	
Cc08_g13960	Putative Dehydration-responsive element-binding protein 1D		3.42	
Cc08_g00740	Ethylene-responsive transcription factor SHINE 3			5.59
Cc04_g02760	Ethylene-responsive transcription factor TINY			2.64
Cc06_g09660	Putative uncharacterized protein			-2.40
Cc04_g01480	Putative Nuclear transcription factor Y subunit A-3			-1.75
Cc06_g16930	Transcription factor			-3.02
Cc10_g02160	Floral homeotic protein DEFICIENS	-4.95		-2.43
Cc01_g09290	Transcription factor TGA2	-3.90		
Cc02_g03420	Putative Ethylene-responsive transcription factor ERF027			-3.30
Cc01_g18390	Ethylene-responsive transcription factor 1B			-3.99

Cc10_g02460	Putative Ocs element-binding factor 1	1.60
Cc00_g21780	Putative Transcription factor VIP1	-1.50
Cc08_g12100	myb-like transcription factor family protein	-1.45
Cc01_g14950	Putative Probable WRKY transcription factor 3	-1.70
Cc02_g05410	Transcription factor RF2b	3.26
Cc08_g09520	Dehydration-responsive element-binding protein 3	-2.52
Cc02_g24840	myb-like HTH transcriptional regulator family protein	6.04
Cc06_g05680	Putative Protein ABSCISIC ACID-INSENSITIVE 5	-4.77
Cc11_g11290	basic leucine-zipper 42	4.78

Table S15. Regulation pattern among DEGs in Icatu plants at 37 ºC, 42 ºC or REC14 temperatures, relative to the control 25 ºC, and submitted to either WW or SWD.

Gene ID	Protein Name	WW			SWD		
		37ºC	42ºC	REC14	37ºC	42ºC	REC14
**Antioxidant activity**							
LOC113701179	peroxidase 51-like	4.40					-6.36
LOC113741493	purple acid phosphatase 17-like		-1.84				
LOC113727829	galactinol synthase 2-like						5.52
LOC113694109	zinc finger CCCH domain-containing protein 20-like						3.24
LOC113733849	galactinol synthase 2-like isoform X3						1.97
LOC113711983	peroxidase 4-like	9.41					
LOC113691066	peroxidase 3-like						5.03
LOC113734308	plastid-lipid-associated protein 6, chloroplastic						-1.39
LOC113733440	WRKY DNA-binding transcription factor 70-like	3.93	-3.63				
LOC113716953	protein PLANT CADMIUM RESISTANCE 2-like	3.14					-2.01
LOC113697429	peroxidase 25-like	9.05					-3.21
LOC113738017	galactinol synthase 2-like	3.59					
LOC113731964	22.0 kDa class IV heat shock protein-like	-14.90	-15.50		-9.19	-8.72	
LOC113735307	alkaline/neutral invertase A, mitochondrial-like						1.50

LOC113695350	NADPH-dependent oxidoreductase 2-alkenal reductase-like					-7.50
LOC113730421	18.2 kDa class I heat shock protein-like	-8.06	-10.14	-6.00	-6.88	
LOC113717780	18.5 kDa class I heat shock protein-like	-6.58	-11.11	-5.48	-6.06	2.90
LOC113715210	thylakoid lumenal 29 kDa protein, chloroplastic-like isoform X2	6.45				
LOC113700787	isoflavone reductase homolog TP7-like					-8.99
LOC113733000	galactinol synthase 2-like					7.90
LOC113727571	phenylalanine ammonia-lyase G4-like	3.19	3.60		2.60	-2.31
LOC113716952	protein PLANT CADMIUM RESISTANCE 2-like					-2.00
LOC113727830	galactinol synthase 2-like		-2.43			7.23
LOC113713509	LOW QUALITY PROTEIN: isoflavone reductase homolog TP7-like					-3.67
LOC113717777	glutathione S-transferase-like					-1.58
LOC113707670	transcription termination factor MTEF1, chloroplastic-like					-2.81
LOC113711139	LOW QUALITY PROTEIN: zinc finger protein ZAT10-like					4.01
LOC113715777	cell number regulator 9-like isoform X2		2.60			-4.25
LOC113729577	17.5 kDa class I heat shock protein-like	-8.17	-9.31	-6.37	-6.51	
LOC113710359	17.3 kDa class I heat shock protein-like	-5.24	-10.54	-5.15	-6.88	1.25
LOC113717790	cell number regulator 7-like	7.13	5.95			-6.94
LOC113692361	LOW QUALITY PROTEIN: class I heat shock protein-like	-7.78	-8.75	-5.24	-5.76	
LOC113730422	17.4 kDa class I heat shock protein-like	-4.59	-6.63	-2.82	-4.11	1.77

LOC113695644	class I heat shock protein-like	-8.84	-10.60		-4.61	-5.30	3.33
LOC113736281	chalcone synthase 2-like	10.26	2.82				-1.78
LOC113710318	peroxidase 42-like	7.11					-4.14
LOC113728228	LOW QUALITY PROTEIN: L-ascorbate peroxidase 2, cytosolic-like	-2.76					
LOC113697049	2-alkenal reductase (NADP(+)-dependent)-like	7.29					-1.79
LOC113706342	glutathione S-transferase T1-like	-3.80	-3.60				1.85
LOC113730287	wound-induced protein 1-like	3.06					
LOC113726525	zinc finger protein ZAT10-like	-4.05	-4.25				4.19
LOC113734036	peptide methionine sulfoxide reductase B5-like	3.78	1.54				-2.39
LOC113694958	cationic peroxidase 1-like	8.28	7.01				
LOC113717816	probable galactinol--sucrose galactosyltransferase 2	4.41	5.01				-3.16
LOC113704304	protein LOL1 isoform X2	3.58	3.34				-2.48
LOC113718216	protein SENESCENCE-ASSOCIATED GENE 21, mitochondrial-like	3.87					
LOC113707447	pleiotropic drug resistance protein 1-like	3.88	2.76				-1.26
LOC113701398	BTB/POZ and TAZ domain-containing protein 4-like	6.36	5.59				
LOC113739654	17.4 kDa class III heat shock protein-like	-2.85	-4.66			-3.10	1.20
LOC113707625	phenylalanine ammonia-lyase G4-like	4.60	3.02				-2.68
LOC113709253	transcription termination factor MTEF1, chloroplastic-like	2.90					-2.23
LOC113699229	UDP-glycosyltransferase 74E2-like	-3.63					

LOC113736192	plastid-lipid-associated protein 6, chloroplastic-like	6.68					
LOC113701327	probable hexokinase-like 2 protein isoform X1	4.86	3.18				-2.85
LOC113701593	PLAT domain-containing protein 3-like	10.33					
LOC113702948	glutathione S-transferase T1-like	5.11	-3.53				
LOC113690410	peroxidase 4-like	2.17	1.82		-4.23		-3.85
LOC113732999	galactinol synthase 2-like		-2.81				2.44
LOC113697788	protein ACTIVITY OF BC1 COMPLEX KINASE 8, chloroplastic-like						-1.82
LOC113692737	mechanosensitive ion channel protein 10-like						-4.13
LOC113700485	protein ACTIVITY OF BC1 COMPLEX KINASE 8, chloroplastic						-1.41
LOC113713327	annexin D1-like						2.36
LOC113730118	peroxidase 66-like						-8.64
LOC113691470	thioredoxin reductase NTRC-like						-1.92
LOC113708682	peroxidase 42-like						-3.23
LOC113697378	temperature-induced lipocalin-1-like		-3.04				1.55
LOC113712193	thylakoid lumenal 29 kDa protein, chloroplastic-like isoform X2						-1.57
LOC113716754	probable galactinol--sucrose galactosyltransferase 2		3.07				-1.88
LOC113716920	fructose-bisphosphate aldolase 3, chloroplastic						1.29
LOC113718814	protein PLANT CADMIUM RESISTANCE 2-like		5.10				-3.44
LOC113726693	peroxidase 43-like		3.32				-3.17

LOC113701871	peroxidase 64-like isoform X1						-9.01
LOC113695405	uncharacterized protein LOC113695405				8.28		4.54
LOC113698367	glutathione hydrolase 1-like		1.88				-2.12
LOC113715779	protein PLANT CADMIUM RESISTANCE 2-like						-3.45
LOC113692853	probable galactinol--sucrose galactosyltransferase 6						1.70
LOC113706341	glutathione S-transferase T1-like						1.30
LOC113736283	chalcone synthase 2		8.74			4.60	
LOC113741763	17.4 kDa class III heat shock protein-like		-5.27			-3.93	
LOC113692559	peroxidase 16-like		1.29			3.64	
LOC113734418	chalcone synthase 2-like		9.21			6.30	
LOC113734419	chalcone synthase 2-like		9.06			4.25	
LOC113730933	S-linalool synthase-like				5.60	2.78	
LOC113717034	leucoanthocyanidin reductase-like isoform X2		2.98			1.36	
LOC113742144	pleiotropic drug resistance protein 1-like		1.84				
**Lipid metabolism (FAD/LOX)**							
LOC113698717	3-ketoacyl-CoA synthase 6-like		3.73			3.83	-3.85
LOC113732840	probable linoleate 9S-lipoxygenase 5	10.90	2.36				-3.60
LOC113714316	allene oxide synthase-like	7.10					
LOC113701649	3-ketoacyl-CoA synthase 6	6.29	2.93				-5.22

LOC113710008	delta(12)-acyl-lipid-desaturase-like	4.34				-3.89
LOC113688131	fatty acid hydroperoxide lyase, chloroplastic-like	3.97			3.37	
LOC113725022	oxalate--CoA ligase-like					3.06
LOC113688924	two pore calcium channel protein 1B-like isoform X2		2.26		1.49	2.12
LOC113734200	3-ketoacyl-CoA synthase 11-like		4.44			-5.28
LOC113725699	cytochrome P450 77A2-like	3.71	-2.08		-3.55	-4.13
LOC113692029	monogalactosyldiacylglycerol synthase 2, chloroplastic-like					
LOC113739952	GDSL esterase/lipase At3g48460	4.52	4.50		4.48	-2.72
LOC113718708	acyl carrier protein 1, chloroplastic-like isoform X2		1.29	-3.65		-2.80
LOC113718905	long chain acyl-CoA synthetase 1-like	4.53	2.92			-3.75
LOC113707236	4-coumarate--CoA ligase-like 7	2.32				
LOC113730063	oxalate--CoA ligase-like	4.38		3.64		2.83
LOC113725542	3-ketoacyl-CoA synthase 19-like	4.91	4.55			-7.48
LOC113732766	oleoyl-acyl carrier protein thioesterase, chloroplastic-like		1.33			-2.89
LOC113718711	3-ketoacyl-CoA synthase 6-like	7.12	4.59			-6.58
LOC113702113	serine/threonine-protein kinase SAPK1-like isoform X3	4.33	3.16			-3.78
LOC113738042	ATP-citrate synthase beta chain protein 2	10.27	1.61			
LOC113743184	two pore calcium channel protein 1B-like isoform X2	6.68	2.80		2.64	2.38
LOC113706907	3-ketoacyl-CoA synthase 10	2.94	2.32			

LOC113730704	cytochrome P450 77A2-like	6.46		-5.83
LOC113700875	linoleate 13S-lipoxygenase 2-1, chloroplastic-like	4.13		
LOC113692843	benzyl alcohol O-benzoyltransferase-like	-4.38		
LOC113713648	oleoyl-acyl carrier protein thioesterase, chloroplastic-like	3.48		-2.33
LOC113702461	probable acyl-activating enzyme 6	8.25	6.91	-8.12
LOC113705072	anthranilate N-benzoyltransferase protein 2-like	8.15	7.65	-6.38
LOC113733368	3-ketoacyl-CoA synthase 1-like	4.53	4.32	-1.84
LOC113714420	jasmonate O-methyltransferase-like isoform X2	5.30	5.59	-5.19
LOC113704294	3-ketoacyl-CoA synthase 11-like	2.89	1.95	
LOC113716426	3-ketoacyl-CoA synthase 6-like	4.04	3.32	-5.06
LOC113703864	cytochrome P450 86A22	5.80		
LOC113697896	probable acyl-activating enzyme 6	5.24	8.17	-7.74
LOC113727628	probable linoleate 9S-lipoxygenase 5	4.18	6.76	
LOC113741991	palmitoyl-monogalactosyldiacylglycerol delta-7 desaturase, chloroplastic-like	2.38		-1.63
LOC113726544	malate dehydrogenase, glyoxysomal-like isoform X1			-2.60
LOC113731577	malate dehydrogenase, glyoxysomal-like			-2.22
LOC113700062	serine/threonine-protein kinase SAPK1-like isoform X3		3.27	-3.61
LOC113706796	protein HOTHEAD-like		2.50	-4.14

LOC113703052	protein HOTHEAD-like						-6.32
LOC113729600	non-symbiotic hemoglobin 2-like						3.09
LOC113730631	omega-3 fatty acid desaturase, endoplasmic reticulum-like		3.34				-2.64
LOC113712916	putative 12-oxophytodienoate reductase 11 isoform X1						-1.93
LOC113705303	uncharacterized protein LOC113705303						-5.32
LOC113710871	jasmonate O-methyltransferase-like						-3.58
LOC113708785	putative 12-oxophytodienoate reductase 11					5.67	
LOC113727527	mediator of RNA polymerase II transcription subunit 15a-like isoform X1					-4.40	
LOC113728215	3-ketoacyl-CoA synthase 1		5.24			4.03	
LOC113732838	probable linoleate 9S-lipoxygenase 5		3.24			5.39	
LOC113695991	benzyl alcohol O-benzoyltransferase-like					-1.27	
LOC113742222	protein HOTHEAD			-3.27			
LOC113709442	putative 12-oxophytodienoate reductase 11				5.57		
LOC113699651	probable acyl-activating enzyme 18, peroxisomal isoform X2		2.84				
LOC113693992	delta(12)-fatty-acid desaturase FAD2-like		2.51				
LOC113707209	LOW QUALITY PROTEIN: cytochrome P450 86A22-like		4.46				
LOC113690394	homogentisate 1,2-dioxygenase	-2.92	-1.88				1.74
LOC113732840	probable linoleate 9S-lipoxygenase 5	10.90	2.36				-3.60
LOC113706389	probable carotenoid cleavage dioxygenase 4, chloroplastic						-1.61

LOC113700875	linoleate 13S-lipoxygenase 2-1, chloroplastic-like	4.13					
LOC113703222	probable carotenoid cleavage dioxygenase 4, chloroplastic	3.51					
LOC113727628	probable linoleate 9S-lipoxygenase 5	4.18	6.76				
LOC113732838	probable linoleate 9S-lipoxygenase 5		3.24			5.39	
**Photosynthesis**							
LOC113723858	LOW QUALITY PROTEIN: transketolase, chloroplastic-like						3.25
LOC113700194	carbonic anhydrase 2-like		2.36				-3.36
LOC113711104	protein STAY-GREEN LIKE, chloroplastic-like		-2.97				
LOC113713402	chloroplast envelope quinone oxidoreductase homolog	2.16					-1.58
LOC113742311	protein CURVATURE THYLAKOID 1C, chloroplastic isoform X1						-1.48
LOC113714316	allene oxide synthase-like	7.10					
LOC113725864	oxygen-evolving enhancer protein 3-2, chloroplastic-like				-4.82		-3.83
LOC113742146	geranylgeranyl diphosphate reductase, chloroplastic						-2.04
LOC113730850	oxygen-evolving enhancer protein 3-2, chloroplastic-like	6.87					-3.96
LOC113712883	chlorophyll a-b binding protein CP26, chloroplastic						-2.89
LOC113734308	plastid-lipid-associated protein 6, chloroplastic						-1.39
LOC113688257	protochlorophyllide reductase, chloroplastic						-1.96
LOC113739975	magnesium protoporphyrin IX methyltransferase, chloroplastic-like						-3.24
LOC113732568	chloroplast envelope quinone oxidoreductase homolog						-2.04

LOC113740295	chlorophyll a-b binding protein P4, chloroplastic-like isoform X2			-2.88
LOC113710985	glutamyl-tRNA reductase 1, chloroplastic-like		-2.92	-3.50
LOC113713503	protein STAY-GREEN LIKE, chloroplastic-like	7.29		-7.65
LOC113702187	oxygen-evolving enhancer protein 1, chloroplastic			-1.59
LOC113708659	chlorophyll a-b binding protein of LHCII type 1-like			-4.24
LOC113736648	tetrapyrrole-binding protein, chloroplastic-like			-3.31
LOC113734866	tetrapyrrole-binding protein, chloroplastic-like			-2.94
LOC113734000	divinyl chlorophyllide a 8-vinyl-reductase, chloroplastic-like isoform X2			-3.42
LOC113726304	photosystem I subunit O-like		-3.03	-2.60
LOC113743471	chlorophyll a-b binding protein 36, chloroplastic	6.78		-2.56
LOC113709767	photosystem I reaction center subunit III, chloroplastic-like			-2.77
LOC113715210	thylakoid lumenal 29 kDa protein, chloroplastic-like isoform X2	6.45		
LOC113689822	chlorophyll a-b binding protein 36, chloroplastic		-2.81	-2.39
LOC113694112	photosystem II 5 kDa protein, chloroplastic-like		-3.70	-2.25
LOC113688109	chlorophyll a-b binding protein 13, chloroplastic		-3.73	-3.98
LOC113705554	photosystem I subunit O-like			-2.05
LOC113736608	divinyl chlorophyllide a 8-vinyl-reductase, chloroplastic isoform X2			-2.54
LOC113738154	probable 1-deoxy-D-xylulose-5-phosphate synthase, chloroplastic			-1.40
LOC113697312	photosystem II 5 kDa protein, chloroplastic-like	6.16		-2.07

LOC113714677	glutamyl-tRNA reductase 1, chloroplastic-like						-3.28
LOC113708898	uncharacterized protein ycf39-like				-3.35		-2.08
LOC113711139	LOW QUALITY PROTEIN: zinc finger protein ZAT10-like						4.01
LOC113696085	magnesium-protoporphyrin IX monomethyl ester [oxidative				-4.13		-3.13
LOC113740565	glutamyl-tRNA reductase-binding protein, chloroplastic-like	2.86					-1.38
LOC113710161	chlorophyll a-b binding protein of LHCII type 1-like	3.76					-3.46
LOC113729356	LOW QUALITY PROTEIN: protein PLASTID TRANSCRIPTIONALLY ACTIVE 16, chloroplastic-like				-3.22		-2.71
LOC113696761	magnesium-protoporphyrin IX monomethyl ester [oxidative						-2.91
LOC113732998	psbP domain-containing protein 1, chloroplastic-like	6.96	1.55				
LOC113704253	zinc transporter 4, chloroplastic-like	6.00	1.56		4.16		
LOC113732783	UPF0603 protein At1g54780, chloroplastic-like	7.09	1.78				
LOC113688118	phosphoglycerate kinase, chloroplastic	6.16	2.20				-1.78
LOC113727176	ribulose bisphosphate carboxylase/oxygenase activase, chloroplastic-like	-4.80	-5.68				4.30
LOC113701851	serine hydroxymethyltransferase, mitochondrial	3.72					
LOC113712834	photosystem I reaction center subunit XI, chloroplastic-like	6.80					-1.53
LOC113741346	photosynthetic NDH subunit of subcomplex B 4, chloroplastic-like	7.10					
LOC113709298	uncharacterized protein ycf39-like	5.68	1.20				-2.15
LOC113702075	magnesium-chelatase subunit ChlI, chloroplastic-like	3.45					-2.33

LOC113709314	isopentenyl-diphosphate Delta-isomerase I-like	10.08	3.21				
LOC113742137	chlorophyll a-b binding protein 4, chloroplastic-like isoform X2	6.80					-2.73
LOC113688601	uroporphyrinogen decarboxylase 1, chloroplastic-like	7.11					-1.87
LOC113736588	LOW QUALITY PROTEIN: early light-induced protein 2, chloroplastic-like	4.32					
LOC113700875	linoleate 13S-lipoxygenase 2-1, chloroplastic-like	4.13					
LOC113731692	chlorophyll a-b binding protein CP24 10A, chloroplastic-like	6.52					-2.57
LOC113698659	ATP-dependent zinc metalloprotease FTSH 6, chloroplastic-like	-6.45	-6.23			-3.48	3.19
LOC113694841	magnesium-chelatase subunit ChlH, chloroplastic-like isoform X2	4.20					-3.76
LOC113732386	ribulose bisphosphate carboxylase/oxygenase activase, chloroplastic-like	-3.52	-4.39				2.46
LOC113734176	early light-induced protein 2, chloroplastic-like isoform X1	-2.64	-2.19				
LOC113726525	zinc finger protein ZAT10-like	-4.05	-4.25				4.19
LOC113743532	photosystem II 10 kDa polypeptide, chloroplastic-like	3.21					-1.43
LOC113731839	probable plastid-lipid-associated protein 4, chloroplastic isoform X3	2.92	2.18				-1.15
LOC113703734	glyoxylate/hydroxypyruvate reductase HPR3-like	5.79	2.22				-2.86
LOC113743395	photosystem II repair protein PSB27-H1, chloroplastic-like	2.22					
LOC113730004	uncharacterized protein PAM68-like	4.46	6.36			4.30	
LOC113709724	ATP synthase gamma chain, chloroplastic-like	2.39					-2.11
LOC113702702	GDT1-like protein 1, chloroplastic	4.24	1.91				
LOC113743800	glucose-6-phosphate/phosphate translocator 2, chloroplastic-like	4.31	5.78				-7.40

LOC113742776	ribulose-phosphate 3-epimerase, chloroplastic	1.86					-1.04
LOC113736192	plastid-lipid-associated protein 6, chloroplastic-like	6.68					
LOC113732375	ylmG homolog protein 1-2, chloroplastic-like	-1.70					1.13
LOC113701593	PLAT domain-containing protein 3-like	10.33					
LOC113698862	probable plastid-lipid-associated protein 12, chloroplastic isoform X3	1.89	2.16				-1.37
LOC113733266	protein CURVATURE THYLAKOID 1A, chloroplastic-like	2.14					-1.76
LOC113702816	plastidal glycolate/glycerate translocator 1, chloroplastic	1.73					
LOC113741991	palmitoyl-monogalactosyldiacylglycerol delta-7 desaturase, chloroplastic-like	2.38					-1.63
LOC113691905	magnesium-chelatase subunit ChlH, chloroplastic				-3.23		-3.77
LOC113701458	ATP-dependent zinc metalloprotease FTSH 6, chloroplastic-like		-6.01			-3.01	3.92
LOC113688543	phosphoenolpyruvate carboxylase-like						-4.36
LOC113690091	glucose-6-phosphate/phosphate translocator 2, chloroplastic-like		5.40				-7.12
LOC113719543	glyceraldehyde-3-phosphate dehydrogenase A, chloroplastic-like						-1.96
LOC113701240	ribulose bisphosphate carboxylase small chain SSU11A, chloroplastic-like isoform X2		2.40				-1.83
LOC113726544	malate dehydrogenase, glyoxysomal-like isoform X1						-2.60
LOC113693755	fructose-1,6-bisphosphatase, chloroplastic-like						-2.21
LOC113697169	fructose-1,6-bisphosphatase, chloroplastic-like				-2.90		-2.62

LOC113728229	protein CURVATURE THYLAKOID 1B, chloroplastic				-2.70
LOC113732850	psbP domain-containing protein 7, chloroplastic-like isoform X2		-2.99		-2.10
LOC113731577	malate dehydrogenase, glyoxysomal-like				-2.22
LOC113741817	photosystem I reaction center subunit VI, chloroplastic-like				-1.66
LOC113689091	oxygen-evolving enhancer protein 2, chloroplastic-like				-1.78
LOC113690443	phosphoglycerate kinase, chloroplastic-like	1.81			-1.82
LOC113691470	thioredoxin reductase NTRC-like				-1.92
LOC113726667	chlorophyll a-b binding protein CP24 10A, chloroplastic		-3.37		-2.69
LOC113738184	glutamine synthetase leaf isozyme, chloroplastic-like				-1.32
LOC113699115	magnesium-chelatase subunit ChlI, chloroplastic-like		-3.02		-1.76
LOC113698165	ribulose bisphosphate carboxylase small chain SSU8, chloroplastic-like	2.05			-1.51
LOC113728157	protein CURVATURE THYLAKOID 1A, chloroplastic-like				-1.64
LOC113703720	phosphoribulokinase, chloroplastic-like				-1.41
LOC113708818	glycerate dehydrogenase-like	1.71			-1.54
LOC113715085	fructose-1,6-bisphosphatase, cytosolic	1.45			-2.14
LOC113706872	phosphoribulokinase, chloroplastic-like				-1.35
LOC113716833	uncharacterized protein LOC113716833 isoform X1			-1.41	-1.55
LOC113712439	glyceraldehyde-3-phosphate dehydrogenase B, chloroplastic				-1.62
LOC113688101	protein THYLAKOID FORMATION1, chloroplastic				-1.51

LOC113710419	photosystem I reaction center subunit psaK, chloroplastic			-1.82
LOC113716728	LOW QUALITY PROTEIN: glyceraldehyde-3-phosphate dehydrogenase A, chloroplastic-like		-2.73	-1.80
LOC113742866	ACT domain-containing protein ACR11-like isoform X1			-1.55
LOC113732043	photosystem I reaction center subunit XI, chloroplastic-like			-1.49
LOC113716478	phosphoenolpyruvate carboxylase 4			-1.73
LOC113712193	thylakoid lumenal 29 kDa protein, chloroplastic-like isoform X2			-1.57
LOC113698105	photosynthetic NDH subunit of lumenal location 4, chloroplastic-like isoform X1	2.70		-2.00
LOC113719027	LOW QUALITY PROTEIN: protoporphyrinogen oxidase, chloroplastic-like			-1.34
LOC113716585	chlorophyll a-b binding protein, chloroplastic-like		-2.81	-1.40
LOC113693667	rhodanese-like domain-containing protein 9, chloroplastic		-2.68	-1.78
LOC113712788	fructose-1,6-bisphosphatase, cytosolic-like			-1.58
LOC113743360	protochlorophyllide reductase-like			-1.37
LOC113701511	peptidyl-prolyl cis-trans isomerase FKBP17-2, chloroplastic-like		-2.86	-1.79
LOC113701098	photosynthetic NDH subunit of lumenal location 4, chloroplastic-like isoform X1	2.82		-1.81
LOC113726476	uncharacterized protein LOC113726476	2.33		-2.64
LOC113715630	photosystem II reaction center W protein, chloroplastic			-1.57
LOC113716041	chlorophyllase-1-like			-3.19

LOC113694681	rhodanese-like domain-containing protein 9, chloroplastic	1.33			-1.53
LOC113734016	probable 1-deoxy-D-xylulose-5-phosphate synthase, chloroplastic				-1.18
LOC113690713	photosystem II 10 kDa polypeptide, chloroplastic		-3.00		-1.15
LOC113725800	transketolase, chloroplastic-like				3.42
LOC113727575	UPF0603 protein At1g54780, chloroplastic-like	2.02			-0.96
LOC113733189	chlorophyllase-1-like		-5.77		-3.04
LOC113691073	phosphoenolpyruvate carboxylase	1.93			-3.16
LOC113691950	protein CHLORORESPIRATORY REDUCTION 6, chloroplastic-like				-1.00
LOC113739930	psbP-like protein 1, chloroplastic isoform X2	1.21			-1.69
LOC113727543	uncharacterized protein LOC113727543 isoform X2	-2.19		-2.62	-1.86
LOC113688851	protochlorophyllide reductase-like				-1.27
LOC113726036	30S ribosomal protein S5, chloroplastic-like				-1.31
LOC113707285	plastidal glycolate/glycerate translocator 1, chloroplastic-like				-0.96
LOC113703289	protein STAY-GREEN, chloroplastic-like	-2.95	4.35		2.29
LOC113727828	psbP domain-containing protein 1, chloroplastic-like				-1.47
LOC113716969	photosystem II core complex proteins psbY, chloroplastic-like				-1.15
LOC113711936	alanine--glyoxylate aminotransferase 2 homolog 3, mitochondrial-like	2.28		2.56	
LOC113689677	photosystem II repair protein PSB27-H1, chloroplastic-like		-2.66		
LOC113706951	probable plastid-lipid-associated protein 12, chloroplastic isoform X2	3.37			

LOC113692757	probable 1-deoxy-D-xylulose-5-phosphate synthase 2, chloroplastic		4.51				
LOC113712265	psbP domain-containing protein 3, chloroplastic isoform X2		1.89				
LOC113716676	photosynthetic NDH subunit of lumenal location 2, chloroplastic		1.88				
LOC113705656	zinc transporter 4, chloroplastic-like		1.87				
LOC113708766	isopentenyl-diphosphate Delta-isomerase I		2.66				
LOC113698106	uncharacterized protein LOC113698106		1.30				
LOC113732386	ribulose bisphosphate carboxylase/oxygenase activase, chloroplastic-like	-3.52	-4.39				2.46
LOC113727176	ribulose bisphosphate carboxylase/oxygenase activase, chloroplastic-like		-5.68				4.30
**Respiration**							
LOC113726544	malate dehydrogenase, glyoxysomal-like isoform X1						-2.60
LOC113731577	malate dehydrogenase, glyoxysomal-like						-2.22
LOC113731758	malate dehydrogenase, cytoplasmic-like						-4.84
LOC113687901	glucan endo-1,3-beta-glucosidase 12						-2.41
LOC113716652	uncharacterized protein LOC113716652		3.42			2.21	3.14
LOC113741792	uncharacterized protein LOC113741792						3.82
LOC113718867	uncharacterized protein LOC113718867	3.66	2.52			2.07	2.71
LOC113698717	3-ketoacyl-CoA synthase 6-like		3.73			3.83	-3.85
LOC113701137	pathogenesis-related protein 5-like	11.22	2.28				-5.17
LOC113714316	allene oxide synthase-like	7.10					

LOC113701649	3-ketoacyl-CoA synthase 6	6.29	2.93			-5.22
LOC113742146	geranylgeranyl diphosphate reductase, chloroplastic					-2.04
LOC113717117	multicopper oxidase LPR2-like					-2.39
LOC113717726	MLP-like protein 34	6.67			2.12	
LOC113700039	thaumatin-like protein 1	2.88	3.40			-5.87
LOC113703366	EG45-like domain containing protein	2.91				-2.29
LOC113717721	multicopper oxidase LPR2-like	3.08				-1.24
LOC113701029	thaumatin-like protein 1	5.05	2.78			-5.38
LOC113694025	mitochondrial carrier protein CoAc2-like	-1.64				2.18
LOC113714501	30S ribosomal protein S9, chloroplastic-like	2.96				
LOC113735307	alkaline/neutral invertase A, mitochondrial-like					1.50
LOC113743471	chlorophyll a-b binding protein 36, chloroplastic	6.78				-2.56
LOC113708913	lysM domain-containing GPI-anchored protein 2-like					-2.11
LOC113689822	chlorophyll a-b binding protein 36, chloroplastic			-2.81		-2.39
LOC113700787	isoflavone reductase homolog TP7-like					-8.99
LOC113718845	alpha-xylosidase 1-like	9.97				-2.84
LOC113701838	pyridoxine/pyridoxamine 5'-phosphate oxidase 1, chloroplastic-like	-1.82	-1.89			2.25
LOC113731758	malate dehydrogenase, cytoplasmic-like					-4.84
LOC113708902	pterin-4-alpha-carbinolamine dehydratase 2, mitochondrial-like isoform X2	-1.64				2.06

LOC113731154	protein NUCLEAR FUSION DEFECTIVE 4-like						-5.30
LOC113688553	fasciclin-like arabinogalactan protein 7						-5.47
LOC113704043	dynamin-related protein 1E-like isoform X2						-4.36
LOC113713509	LOW QUALITY PROTEIN: isoflavone reductase homolog TP7-like						-3.67
LOC113692063	subtilisin-like protease SBT1.8		2.86				-4.55
LOC113738189	elongation factor Tu, mitochondrial-like	-3.19					
LOC113739943	peptidyl-prolyl cis-trans isomerase CYP26-2, chloroplastic-like isoform X3				-4.04		-3.41
LOC113732340	protein RETICULATA-RELATED 4, chloroplastic-like isoform X2	5.95	1.38				-1.20
LOC113736371	glycine-rich RNA-binding protein 4, mitochondrial-like	5.99					1.65
LOC113739647	heat shock 70 kDa protein-like	-10.37	-11.85		-2.90	-4.18	5.05
LOC113688118	phosphoglycerate kinase, chloroplastic	6.16	2.20				-1.78
LOC113701851	serine hydroxymethyltransferase, mitochondrial	3.72					
LOC113718711	3-ketoacyl-CoA synthase 6-like	7.12	4.59				-6.58
LOC113719667	kirola-like	9.22	3.45				
LOC113700985	AAA-ATPase ASD, mitochondrial-like	10.60					-2.37
LOC113715606	HIPL1 protein-like isoform X2	5.71					-2.83
LOC113743529	uncharacterized protein LOC113743529	5.02					
LOC113702480	glutamate decarboxylase-like	3.75	2.62				
LOC113710291	two-component response regulator-like PRR37 isoform X2	6.43					-2.00

LOC113694841	magnesium-chelatase subunit ChlH, chloroplastic-like isoform X2	4.20					-3.76
LOC113743195	grpE protein homolog 1, mitochondrial-like isoform X2	8.21	-2.41			-1.41	
LOC113733163	AT-hook motif nuclear-localized protein 9-like	8.08	2.53				
LOC113713838	probable beta-D-xylosidase 7	7.54					-5.80
LOC113718296	cytochrome P450 71A1-like	6.95	4.12				-8.21
LOC113710048	LOW QUALITY PROTEIN: BAG family molecular chaperone regulator 3-like	2.01	2.35				
LOC113719591	cysteine-rich receptor-like protein kinase 3 isoform X2	6.31					-1.69
LOC113731027	protein IQ-DOMAIN 1-like	5.66	2.42				
LOC113719669	transmembrane 9 superfamily member 2-like isoform X3	7.81					-2.49
LOC113697465	protein phosphatase 2C and cyclic nucleotide-binding/kinase domain-containing protein-like isoform X2	4.06					
LOC113743532	photosystem II 10 kDa polypeptide, chloroplastic-like	3.21					-1.43
LOC113718216	protein SENESCENCE-ASSOCIATED GENE 21, mitochondrial-like	3.87					
LOC113701085	AAA-ATPase At3g50940-like	4.16	2.46				
LOC113716426	3-ketoacyl-CoA synthase 6-like	4.04	3.32				-5.06
LOC113740490	hydroxymethylglutaryl-CoA lyase, mitochondrial-like	-2.71					
LOC113717763	transmembrane 9 superfamily member 2-like isoform X1	-2.49			-4.05		
LOC113694151	stress-response A/B barrel domain-containing protein UP3-like	3.23	1.83				-1.60

LOC113709724	ATP synthase gamma chain, chloroplastic-like	2.39					-2.11
LOC113689879	elongation factor Ts, mitochondrial isoform X2	-2.60	-2.25				
LOC113695736	actin-1	3.94	2.65				-2.04
LOC113709680	aminomethyltransferase, mitochondrial	2.99	2.59				-3.02
LOC113698415	TMV resistance protein N-like	4.66			4.61		
LOC113732077	NADH dehydrogenase [ubiquinone	1.85					0.84
LOC113701327	probable hexokinase-like 2 protein isoform X1	4.86	3.18				-2.85
LOC113710262	mitochondrial carnitine/acylcarnitine carrier-like protein	1.93	1.19				-2.09
LOC113691905	magnesium-chelatase subunit ChlH, chloroplastic				-3.23		-3.77
LOC113708606	aminomethyltransferase, mitochondrial-like		1.79				-2.14
LOC113688543	phosphoenolpyruvate carboxylase-like						-4.36
LOC113701240	ribulose bisphosphate carboxylase small chain SSU11A, chloroplastic-like isoform X2		2.40				-1.83
LOC113726544	malate dehydrogenase, glyoxysomal-like isoform X1						-2.60
LOC113731577	malate dehydrogenase, glyoxysomal-like						-2.22
LOC113713327	annexin D1-like						2.36
LOC113708959	mitochondrial carnitine/acylcarnitine carrier-like protein		1.14				-1.59
LOC113692720	defensin-like protein P322				3.26	7.02	3.37
LOC113730638	LOW QUALITY PROTEIN: internal alternative NAD(P)H-ubiquinone						-1.51

	oxidoreductase A1, mitochondrial-like				
LOC113690443	phosphoglycerate kinase, chloroplastic-like	1.81			-1.82
LOC113711255	internal alternative NAD(P)H-ubiquinone oxidoreductase A1, mitochondrial-like				-1.62
LOC113709204	pterin-4-alpha-carbinolamine dehydratase 2, mitochondrial-like isoform X2				2.07
LOC113738184	glutamine synthetase leaf isozyme, chloroplastic-like				-1.32
LOC113698165	ribulose bisphosphate carboxylase small chain SSU8, chloroplastic-like	2.05			-1.51
LOC113692324	dynamin-related protein 4C-like				-4.01
LOC113708818	glycerate dehydrogenase-like	1.71			-1.54
LOC113688101	protein THYLAKOID FORMATION1, chloroplastic				-1.51
LOC113690330	multiple organellar RNA editing factor 8, chloroplastic/mitochondrial-like				1.44
LOC113742355	thaumatin-like protein	4.18			-8.50
LOC113713775	LOW QUALITY PROTEIN: endoplasmin homolog	-3.37		-1.84	-2.40
LOC113697378	temperature-induced lipocalin-1-like	-3.04			1.55
LOC113716478	phosphoenolpyruvate carboxylase 4				-1.73
LOC113707439	LRR receptor-like serine/threonine-protein kinase ERECTA isoform X1	3.26			-3.04
LOC113731180	snakin-2	4.55		4.21	4.33
LOC113742829	peptidyl-prolyl cis-trans isomerase CYP26-2, chloroplastic isoform X1				-4.29
LOC113726265	thaumatin-like protein 1	3.05			-7.19

LOC113704105	aspartate aminotransferase, cytoplasmic				1.56
LOC113726261	protein NUCLEAR FUSION DEFECTIVE 4-like	2.70			-2.46
LOC113731226	kinesin-like protein KIN-7D, mitochondrial	2.53			-2.54
LOC113695217	ribosomal protein S2, mitochondrial-like isoform X1	5.48			-1.84
LOC113716920	fructose-bisphosphate aldolase 3, chloroplastic				1.29
LOC113725498	methionine--tRNA ligase, chloroplastic/mitochondrial-like	1.67			-1.69
LOC113717245	thioredoxin H2-like	8.60		6.21	-2.56
LOC113690713	photosystem II 10 kDa polypeptide, chloroplastic		-3.00		-1.15
LOC113712215	probable beta-D-xylosidase 7				-4.46
LOC113707354	EG45-like domain containing protein				-2.72
LOC113726116	protein IQ-DOMAIN 1-like	2.24			-2.18
LOC113688192	kinesin-like protein KIN-14F isoform X3	2.30			-2.95
LOC113691073	phosphoenolpyruvate carboxylase	1.93			-3.16
LOC113699382	protein IQ-DOMAIN 31-like				-1.55
LOC113694288	protein IQ-DOMAIN 14				-1.82
LOC113730185	heat shock 22 kDa protein, mitochondrial-like	-4.61			1.40
LOC113707866	bifunctional dihydrofolate reductase-thymidylate synthase-like isoform X4	1.79			-1.80
LOC113726036	30S ribosomal protein S5, chloroplastic-like				-1.31
LOC113701531	AAA-ATPase ASD, mitochondrial-like	-3.30			-3.30

LOC113698881	leucine-rich repeat receptor-like serine/threonine-protein kinase BAM1					-1,92
LOC113712916	putative 12-oxophytodienoate reductase 11 isoform X1					-1,93
LOC113715194	calcium uniporter protein 4, mitochondrial-like				3.09	1,11
LOC113702672	LRR receptor-like serine/threonine-protein kinase ERECTA	2.69				-2,32
LOC113708785	putative 12-oxophytodienoate reductase 11				5.67	
LOC113711936	alanine--glyoxylate aminotransferase 2 homolog 3, mitochondrial-like	2.28			2.56	
LOC113727738	putative pentatricopeptide repeat-containing protein At1g12700, mitochondrial			4.70	2.91	
LOC113717034	leucoanthocyanidin reductase-like isoform X2	2.98			1.36	
LOC113709442	putative 12-oxophytodienoate reductase 11			5.57		
LOC113697364	probable alpha,alpha-trehalose-phosphate synthase [UDP-forming	-5.85				
LOC113725158	heat shock 22 kDa protein, mitochondrial-like	-4.47				
LOC113703085	glycine cleavage system H protein, mitochondrial-like	1.65				
LOC113712265	psbP domain-containing protein 3, chloroplastic isoform X2	1.89				
LOC113736968	kirola-like	3.46				
LOC113730505	heat shock cognate 70 kDa protein 2-like	-2.46				
LOC113714791	hexokinase-2, chloroplastic	6.81				
LOC113716835	kirola-like	6.64				

Table S16. Regulation pattern among DEGs in CL153 plants at 37 ºC, 42 ºC or REC14 temperatures, relative to the control 25 ºC, and submitted to either WW or SWD.

Gene ID	Protein Name	WW			SWD		
		37ºC	42ºC	REC14	37ºC	42ºC	REC14
	**Antioxidant**						
Cc04_g04320	Putative Tetratricopeptide repeat (TPR)-like superfamily protein	-2.67	-2.35				
Cc06_g03490	L-ascorbate peroxidase 2, cytosolic	4.90	7.85				
Cc10_g00570	Catalase		2.48				-2.13
Cc06_g00570	Cationic peroxidase 1	-3.29	-3.29				
Cc06_g13090	Peroxidase 16		-2.03				2.71
Cc07_g13330	15.7 kDa heat shock protein, peroxisomal	3.11			-4.99	5.05	
Cc09_g01700	Peroxidase 42	3.10		2.13		3.37	3.04
Cc06_g12530	20 kDa chaperonin, chloroplastic	1.80			-2.47	2.76	
Cc02_g21960	Peroxidase 43	3.48					2.96
Cc01_g19740	Probable glutathione peroxidase 4	-1.26					
Cc05_g04630	Aconitate hydratase 2, mitochondrial	-1.76					
Cc07_g11210	Peroxidase 64	3.08					4.66
Cc10_g15150	Peroxidase 12				8.94	-8.92	
Cc07_g14740	Peroxidase 17					-4.70	

Cc10_g15140	Peroxidase 12		5.61	-8.07	
Cc08_g11220	peptidyl-prolyl cis-trans isomerases		-2.41	2.67	2.88
Cc09_g02320	Elongator complex protein 6		2.65	-2.52	
Cc07_g13520	NTA15 protein		-1.60	1.82	
Cc08_g15360	Respiratory burst oxidase homolog protein A			-2.80	
Cc07_g02770	Probable galacturonosyltransferase-like 9		7.04		
Cc04_g09270	Purple acid phosphatase 17		2.60		
Cc01_g15110	Peroxidase 4		4.05		
Cc07_g06870	Peroxidase 73		3.39		2.88
Cc01_g08210	Peroxidase 64		-3.14		
Cc06_g18010	Hypothetical protein		-3.13		
Cc05_g08480	Peroxidase 4				2.28
Cc07_g02590	Peroxidase 63				2.97
Cc07_g11020	L-ascorbate peroxidase 3, peroxisomal				7.27
Cc02_g05640	Peroxidase 4	4.56			
Cc00_g17550	Peroxidase 3				-2.38
Cc03_g02760	Peptide methionine sulfoxide reductase B3, chloroplastic				4.51
Cc06_g12610	Peroxiredoxin Q, chloroplastic				2.75
Cc00_g20670	NAD(P)H dehydrogenase 18				2.56
Cc07_g02500	Peroxidase superfamily protein				-1.89

**Lipid metabolism (FAD/LOX)**							
Cc10_g11850	Long chain acyl-CoA synthetase 9, chloroplastic	1.98					
Cc10_g05850	Acetyl-CoA carboxylase 1	1.29		2.15			
Cc11_g10190	3-ketoacyl-CoA synthase 6	1.99			-2.95		3.72
Cc00_g14020	3-ketoacyl-CoA synthase 21	1.99					5.65
Cc11_g10180	3-ketoacyl-CoA synthase 6					-4.52	
Cc03_g04540	3-ketoacyl-CoA synthase 17				8.34	-7.65	
Cc07_g08840	3-ketoacyl-CoA synthase 6				-4.23	5.45	3.85
Cc01_g14330	Omega-3 fatty acid desaturase, endoplasmic reticulum				3.38	-2.52	
Cc06_g09070	3-ketoacyl-CoA synthase 11				2.47	-2.64	
Cc08_g11520	Putative HXXXD-type acyl-transferase family protein					6.69	8.44
Cc03_g04570	3-ketoacyl-CoA synthase 17						3.87
Cc11_g17510	Acyl carrier protein 1, chloroplastic						3.00
Cc01_g16760	Oleoyl-acyl carrier protein thioesterase, chloroplastic (Fragment)						1.99
Cc08_g07790	3-ketoacyl-CoA synthase 10						2.44
Cc01_g19410	Chalcone-flavanone isomerase family protein						2.18
Cc02_g37010	Putative Lysophosphatidylcholine acyltransferase 2						-1.52
Cc02_g39890	3-ketoacyl-CoA synthase 1						2.05
Cc04_g04380	Palmitoyl-monogalactosyldiacylglycerol delta-7 desaturase, chloroplastic						1.51

Cc08_g05610	Probable carotenoid cleavage dioxygenase 4, chloroplastic			-7.54	6.29	6.14
Cc02_g33790	Probable linoleate 9S-lipoxygenase 5			6.75		
Cc03_g04260	1,2-dihydroxy-3-keto-5-methylthiopentene dioxygenase 1			-1.78		
Cc05_g01440	4-hydroxyphenylpyruvate dioxygenase					-2.16
	**Photosynthesis**					
Cc08_g05240	Protein PAM68, chloroplastic		-2.50			2.70
Cc11_g10360	30S ribosomal protein 3, chloroplastic	-1.78				
Cc06_g12530	20 kDa chaperonin, chloroplastic	1.80		-2.47	2.76	
Cc07_g18480	NAD(P)H	-1.75				
Cc02_g08380	Putative PsbP-like protein 1, chloroplastic	-1.23				
Cc04_g16410	Chlorophyll a-b binding protein 4, chloroplastic			-6.64	6.00	5.97
Cc09_g08490	Photosystem I reaction center subunit psaK, chloroplastic			-4.74	5.11	5.80
Cc01_g08780	photosystem I subunit O			-4.24	4.94	4.83
Cc06_g11950	photosystem II subunit X			-4.87	6.42	6.64
Cc05_g12720	Chlorophyll a-b binding protein 13, chloroplastic			-4.84	4.97	5.79
Cc05_g09930	Chlorophyll a-b binding protein 8, chloroplastic			-3.75	3.38	3.18
Cc11_g16910	Chlorophyll a-b binding protein, chloroplastic			-3.35	3.43	3.17
Cc10_g16210	Chlorophyll a-b binding protein CP26, chloroplastic			-4.54	4.40	4.28
Cc10_g12590	Photosystem I reaction center subunit N, chloroplastic			-3.80	4.04	4.59

Cc06_g22740	Magnesium-protoporphyrin IX monomethyl ester [oxidative] cyclase, chloroplastic		-3.92	3.80	4.27
Cc08_g01300	photosystem II subunit X		-3.33	5.00	5.38
Cc05_g15930	Photosystem II 10 kDa polypeptide, chloroplastic			3.25	2.60
Cc03_g03590	Photosystem I reaction center subunit II, chloroplastic		-2.94	3.46	3.04
Cc02_g11770	Oxygen-evolving enhancer protein 3-2, chloroplastic		-3.54	4.14	4.59
Cc09_g09020	Chlorophyll a-b binding protein 21, chloroplastic		-5.70	5.36	6.01
Cc09_g02010	Chlorophyll a-b binding protein 6A, chloroplastic		-4.63	4.25	3.99
Cc09_g06610	Photosystem I reaction center subunit III, chloroplastic		-3.45	3.32	3.56
Cc06_g17100	Putative Magnesium-chelatase subunit H		-3.63	3.28	3.70
Cc05_g06070	Protein THYLAKOID FORMATION1, chloroplastic		-2.29	2.46	2.32
Cc11_g16230	Photosystem II core complex proteins psbY, chloroplastic		-3.18	3.16	3.58
Cc00_g12110	Protein of unknown function (DUF1118)			3.50	3.25
Cc02_g21720	Chlorophyll a-b binding protein CP24 10A, chloroplastic		-3.85	3.64	4.03
Cc04_g07800	Calcium sensing receptor, chloroplastic		-2.23	2.61	2.42
Cc05_g06850	Protochlorophyllide reductase, chloroplastic		-3.41	2.90	2.31
Cc04_g16560	Geranylgeranyl diphosphate reductase, chloroplastic		-3.00	2.84	3.06
Cc06_g04850	Peptidyl-prolyl cis-trans isomerase FKBP16-4, chloroplastic		-1.62	1.98	2.09
Cc01_g19080	Putative Chlorophyllase-1	-2.46	-4.00		5.13
Cc06_g01460	Chlorophyll a-b binding protein CP29.2, chloroplastic		-3.78		3.06
Cc09_g09500	Chlorophyll a-b binding protein 36, chloroplastic		-3.10		3.43

Cc02_g07500	Ribulose bisphosphate carboxylase/oxygenase activase 1, chloroplastic	-2.81	
Cc02_g00040	Putative RNA polymerase sigma-B factor	-1.96	
Cc02_g01060	Cytochrome b5 isoform 1	1.98	
Cc10_g04190	Chlorophyll a-b binding protein P4, chloroplastic	-4.20	5.92
Cc05_g12370	Protochlorophyllide reductase, chloroplastic	-2.56	
Cc09_g09030	Chlorophyll a-b binding protein 21, chloroplastic	-3.47	4.22
Cc01_g17500	Photosystem I reaction center subunit IV A, chloroplastic	-2.44	
Cc09_g09010	Chlorophyll a-b binding protein 21, chloroplastic	-6.21	6.86
Cc07_g00260	Chlorophyll a-b binding protein 13, chloroplastic	-7.21	
Cc01_g11570	ATP synthase subunit d, mitochondrial	1.53	
Cc10_g00150	Hypothetical protein	-2.14	
Cc05_g06750	Thylakoid lumenal protein At1g03610, chloroplastic	-2.10	2.90
Cc05_g09650	Chlorophyll a-b binding protein 1, chloroplastic		9.94
Cc02_g17970	Protein TIC 62, chloroplastic		2.78
Cc02_g18220	cytochrome b6f complex subunit (petM), putative		4.12
Cc00_g15710	Ribulose bisphosphate carboxylase small chain SSU11A, chloroplastic		2.75
Cc11_g14950	Peptidyl-prolyl cis-trans isomerase		2.32
Cc01_g18800	Photosystem II reaction center W protein, chloroplastic		3.47
Cc10_g01080	Plastocyanin, chloroplastic		3.24
Cc05_g00840	Oxygen-evolving enhancer protein 2, chloroplastic		2.92

Cc02_g32670	UPF0603 protein At1g54780, chloroplastic						2.87
Cc02_g09420	INVOLVED IN						4.27
Cc10_g06080	ATP synthase delta chain, chloroplastic						2.51
Cc01_g15890	Photosystem I reaction center subunit XI, chloroplastic						3.06
Cc02_g38860	Uncharacterized protein At4g01150, chloroplastic						2.51
Cc02_g31060	Photosystem II reaction center PsbP family protein						2.45
Cc04_g16320	NDH dependent flow 6						3.15
Cc02_g35130	PsbP domain-containing protein 1, chloroplastic						2.05
Cc01_g17770	Photosystem II reaction center PSB28 protein, chloroplastic						3.41
Cc00_g20670	NAD(P)H dehydrogenase 18						2.56
Cc04_g05520	Unknown protein DS12 from 2D-PAGE of leaf, chloroplastic						2.48
Cc01_g08950	Putative NAD(P)H-quinone oxidoreductase subunit N						2.84
Cc02_g33910	Predicted protein (Fragment)						2.26
Cc00_g15710	Ribulose bisphosphate carboxylase small chain SSU11A, chloroplastic						2.75
**Respiration**							
Cc02_g20400	Malate dehydrogenase, glyoxysomal						2.92
Cc06_g10370	NADP-dependent malic enzyme						-1.56
Cc00_g08170	Putative Heat shock protein 90	1.99	3.11		-1.55		
Cc02_g38200	Cysteine synthase, chloroplastic/chromoplastic		-2.25		2.49	-1.78	

Cc05_g14280	Co-chaperone GrpE family protein	1.26	2.76				
Cc01_g04810	Protein of unknown function (DUF3411)		-1.66				
Cc00_g24390	Putative Phospholipase A1-Igamma1, chloroplastic	-4.24	-3.95				
Cc05_g13380	Electron transfer flavoprotein subunit alpha, mitochondrial		1.66				
Cc04_g03450	Pentatricopeptide repeat-containing protein At3g16010		3.83				
Cc02_g27440	Putative Chaperone protein dnaJ 1, mitochondrial		1.62				
Cc07_g14260	Putative Probable mitochondrial 2-oxoglutarate/malate carrier protein		-1.68				3.44
Cc06_g04390	Dual specificity phosphatase Cdc25		-1.67				
Cc01_g10720	RuBisCO large subunit-binding protein subunit alpha, chloroplastic	2.05			-2.16		
Cc06_g12530	20 kDa chaperonin, chloroplastic	1.80			-2.47	2.76	
Cc02_g24520	Malate dehydrogenase, cytoplasmic	5.52					
Cc05_g04630	Aconitate hydratase 2, mitochondrial	-1.76					
Cc00_g27170	Putative Protein of unknown function (DUF607)	-1.86			1.58	-2.82	
Cc06_g01770	Putative Mitochondrial uncoupling protein 3	-1.47					
Cc08_g16670	UPF0160 protein MYG1, mitochondrial	-1.83					
Cc04_g15520	Putative Mitochondrial outer membrane protein porin of 36 kDa	-2.00		-3.50			8.30
Cc01_g05820	Putative Protein grpE				-2.01	1.77	
Cc04_g07800	Calcium sensing receptor, chloroplastic				-2.23	2.61	2.42
Cc06_g11140	Putative Graves disease carrier protein					-1.73	
Cc04_g09230	Pentatricopeptide repeat-containing protein At4g01400, mitochondrial				-3.32		

Cc07_g05140	Hypothetical protein	2.08	
Cc10_g14110	Putative Polynucleotidyl transferase, ribonuclease H-like superfamily protein	1.98	
Cc05_g07470	ADP,ATP carrier protein 1, mitochondrial	6.39	
Cc01_g11570	ATP synthase subunit d, mitochondrial	1.53	
Cc11_g10810	Phosphoenolpyruvate carboxylase 4	-2.09	1.91
Cc03_g07770	Protein of unknown function (DUF185)	2.29	
Cc00_g01650	Ribosomal protein S7, mitochondrial	-3.52	
Cc11_g16560	DNA polymerase alpha 2	3.66	
Cc08_g00320	Putative Calcium uptake protein 1, mitochondrial	1.41	
Cc05_g11180	Phosphoenolpyruvate carboxylase		4.47
Cc09_g01490	Aminomethyltransferase, mitochondrial		3.55
Cc08_g01750	Cytochrome c oxidase subunit 6b-3		4.27
Cc09_g06160	Formate dehydrogenase, mitochondrial		2.20
Cc02_g20400	Malate dehydrogenase, glyoxysomal		2.92
Cc01_g19410	Chalcone-flavanone isomerase family protein		2.18
Cc11_g15490	Alpha-glucan water dikinase, chloroplastic		1.75
Cc06_g14540	DNA-directed RNA polymerase 3, chloroplastic		-1.58
Cc08_g02390	Glycine cleavage system H protein, mitochondrial		2.82

Table S17. Primers used in this study for qRT-PCR.

**Locus**	**Primer Name**	**Sequence (5′–3′)**
LOC113703008	*PP2C51-F*	CCGAGTACGGAAGCTGCAAGAAGT
	PP2C51-R	GCAGCGATAGTGGCAGGGAAACAA
LOC113740436	*LEADC3_F*	CTACGGCTACTGTCAACGATGTGAA
	LEADC3_R	ATGGGTAAAGAATGGCTGTATCCCT
Cc07_g10030	*DH1a-F*	CGGTCACAAGGAGGCTCAA
	DH1a-R	GTATCCAGGAGCTGCAGTAGCA
LOC113735267	*SUS2-F*	ACTCTGCGGCAATG TAAACT
	SUS2-R	AGACTGCCGCGGAGACCAGA
LOC113707992	*PIP2-F*	ATGTCACATACGGCGGAG
	PIP2-R	TTGGGATCAGTGGCAGAG
Cc07_g07560	*XTH6-F*	CGGTCACCGCTTTCTTTTCAGTT
	XTH6-R	CAAGAACTCAAAATCTAGCTCGT
LOC113727829	*GOLS2-F*	ATGGGATCGATGGAAATGAACTT
	GOL2-R	ACTTCCTGCCAAGAATGTCAC
LOC113727514	*CuSOD1-F*	CCCTTGGAGACACAACGAAT
	CuSOD1-R	GGCAGTACCATCTTGACCA
LOC113728228	*APX_{Chl}-F*	CACCTGCTGCTCATTTACG
	APX_{Chl}-R	GACCTTCCCAATGTGTGTG

PP2C51: protein phosphatase 2C 51-like; *LEADC3*: late embryogenesis abundant protein Dc3-like; *DH1a*: Dehydrin DH1a; *SUS2*: sucrose synthase 2-like; *PIP2*: aquaporin PIP2-2-like; *XTH6*: 6 xyloglucan endotransglucosylase/hydrolase protein; *GOLS2*: galactinol synthase 2-like; *CuSOD1*: Superoxide dismutase [Cu-Zn]; *APXChl*: Chloroplast ascorbate peroxidase.
